# Isohexide-Based Tunable Chiral Platforms as Amide- and Thiourea-Chiral Solvating Agents for the NMR Enantiodiscrimination of Derivatized Amino Acids

**DOI:** 10.3390/molecules29061307

**Published:** 2024-03-15

**Authors:** Federica Cefalì, Anna Iuliano, Federica Balzano, Gloria Uccello Barretta, Valerio Zullo, Carlo Baldassari

**Affiliations:** Department of Chemistry and Industrial Chemistry, University of Pisa, Via G. Moruzzi 13, 56124 Pisa, Italy; f.cefali@studenti.unipi.it (F.C.); gloria.uccello.barretta@unipi.it (G.U.B.); valeriozullo@hotmail.it (V.Z.); c.baldassari@studenti.unipi.it (C.B.)

**Keywords:** absolute configuration, chiral auxiliary, enantiomeric purity, chiral pool, NMR spectroscopy

## Abstract

New arylamide- and arylthiourea-based chiral solvating agents (CSAs) were synthesized starting from commercially available isomannide and isosorbide. The two natural isohexides were transformed into the three amino derivatives, having isomannide, isosorbide, and isoidide stereochemistry, then the amino groups were derivatized with 3,5-dimethoxybenzoyl chloride or 3,5-bis(trifluoromethyl)phenyl isothiocyanate to obtain the CSAs. Bis-thiourea derivative containing the 3,5-bis(trifluoromethyl)phenyl moiety with exo–exo stereochemistry was remarkably efficient in the differentiation of NMR signals (NH and acetyl) of enantiomers of *N*-acetyl (*N*-Ac) amino acids in the presence of 1,4-diazabicyclo[2,2,2]octane (DABCO). Nonequivalences in the ranges of 0.104–0.343 ppm and 0.042–0.107 ppm for NH and acetyl groups, respectively, allowed for very accurate enantiomeric excess determination, and a reliable correlation was found between the relative positions of signals of enantiomers and their absolute configuration. Therefore, a complete stereochemical characterization could be performed. Dipolar interactions detected in the ternary mixture CSA/*N*-Ac-valine/DABCO led to the identification of a different interaction model for the two enantiomers, involving the formation of a one-to-one substrate/CSA complex for (*S*)-*N*-Ac-valine and a one-to-two complex for (*R*)-*N*-Ac-valine, as suggested by the complexation stoichiometry.

## 1. Introduction

The growing awareness of the impact of chirality on human life has prompted the research towards accurate, reproducible, and, if possible, direct methods for the determination of the enantiomeric purity of chiral compounds.

Over the years, chromatographic [1,2,3,4,5] and spectroscopic/spectrometric [6,7,8,9,10,11,12,13] methods have been developed and, among the latter, NMR spectroscopy has emerged as one of the most versatile investigation techniques. Beyond its analytical potentialities, the supremacy of NMR spectroscopy is also due to the possibility of easy investigation of the discrimination processes at a molecular level.

NMR methods of enantiodifferentiation are based on the use of chiral auxiliaries to convert the enantiomers into intrinsically anisochronous diastereomers. Chiral auxiliaries for NMR spectroscopy can be grouped in three classes, based on the nature of the interactions stabilizing the diastereomers: chiral solvating agents (CSAs) [11,12], chiral derivatizing agents (CDAs) [12], and chiral lanthanide shift agents (CLSRs) [12]. The first class emerged for ease of use. CSAs commonly interact with the enantiomeric substrates by means of non-covalent interactions, such as dipole–dipole, π–π and hydrogen bond formation, giving rise to couples of transient diastereomers. A notable exception is represented by chiral liquid crystals (CLCs), which offer unprecedented versatility due to the fact that the substrate does not require any specific, directed interaction with the liquid crystal. Consequently, CLCs have the potential to differentiate a wide range of chiral molecules, encompassing organic and inorganic compounds, as well as metal complexes. One peculiarity of CLCs is their capability to differentiate nuclei that are remote from the chiral center, which presents a challenge for the majority of CSAs [13,14].

For the enantiodifferentiation of chiral substrates by any other kind of CSAs, hydrogen bond formation is mandatory for the achievement of the transient diastereomers. Therefore, CSAs endowed with amide and especially thiourea functions should be privileged due to the enhanced acidity and hence hydrogen bond-donating capabilities of these moieties. These functionalities can be easily embedded in chiral platforms by using simple synthetic procedures, which entail the reaction of a chiral amine or diamine with acyl chlorides or isothiocyanates [15,16].

Chiral diamine obtained from precursors belonging to chiral pool can represent an attractive choice for the synthesis of bis-amide and bis-thiourea CSAs, as their natural precursors are readily available in enantiomerically pure forms.

**Figure 1:**
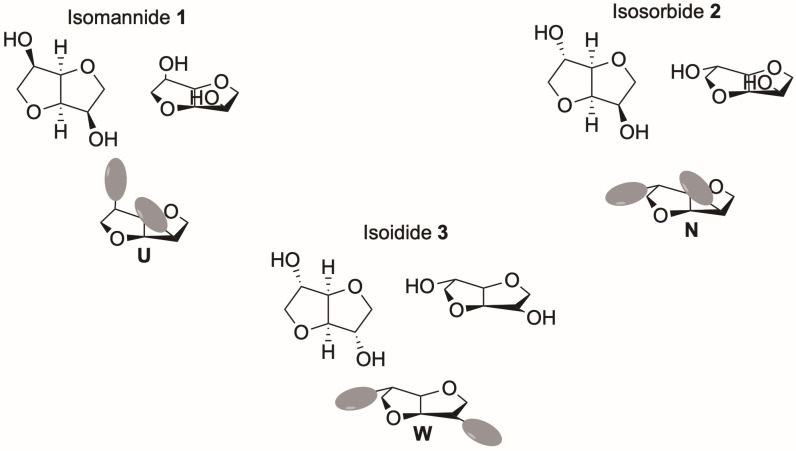
Structures of isohexides and their derivatives.

Isohexides, namely isomannide (**1**, Figure 1) and isosorbide (**2**, Figure 1), which are chiral compounds coming from the chiral pool and endowed with interesting proprieties (cheap, non-toxic and obtained from renewable resources) [17,18,19], and also isoidide (**3**, Figure 1), which can be obtained from isomannide, possess structural features making them good candidates for use as chiral scaffolds for the preparation of chiral auxiliaries for enantioselective processes [20,21,22,23]. Their vaulted structure, endowed with two hydroxyl groups characterized by different relative stereochemistry in different isohexides, allows the creation of U-, N- or W-shaped derivatives (Figure 1) showing interesting enantiorecognition features dependent not only on the nature of the derivatizing moieties but also on the stereochemistry of the final compounds.

**Scheme 1:**
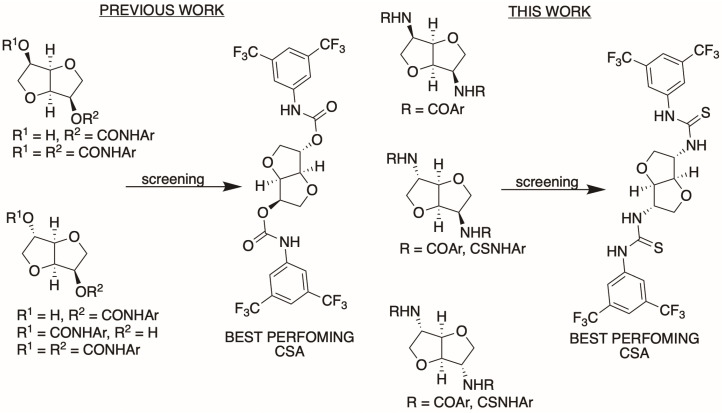
Use of isohexide derivatives as CSAs.

Ionic liquids derived from U-shaped isomannide behaved as chiral tweezers [23], whereas N-shaped aryl carbamates of isosorbide were successfully used as CSAs for the determination of the enantiomeric composition of amino acid derivatives [22] (Scheme 1, left panel). Those results clearly showed that tuning of the structural features allowed well performing CSAs to be obtained and that the presence of two aryl moieties on the isohexide scaffold was mandatory to achieve good enantiodiscrimination.

Starting from these previously reported results, as a part of our ongoing research into the preparation of new, simple, biobased, and efficient CSAs for NMR applications, we turned our attention to the use of amino-derived compounds, such as arylamides or arylthioureas (Scheme 1, right panel). This choice was made while also considering that U-, N-, or W-shaped amino derivatives of isohexides can be easily obtained when starting from commercially available isomannide and isosorbide through stereo-controlled interconversion of the hydroxyl groups to amino groups, followed by the introduction of aryl moieties by amide or thiourea bond formation.

Chiral amides [24,25,26,27,28,29] and especially chiral arylthioureas [30,31,32,33,34,35,36,37,38] are known to exhibit excellent enantiodiscriminating capabilities when employed as CSAs for the NMR enantiodiscrimination of chiral carboxylic acids [30,31,32,33,34,35] and derivatives of amino acids [35,36,37,38].

Herein, we report the synthesis of U-, N- and W-shaped isohexide derived arylamides (Scheme 1) and preliminary screening aimed at finding the most suitable structures for NMR enantiodiscrimination, with the synthesis of arylthioureas having the selected structures, followed by additional fast screening to identify the best performing CSA. The W-shaped arylthiourea molecule emerged as the best performing CSA, and this was finally tested in the enantiodiscrimination of amides of different amino acids under optimized analysis conditions. A complete characterization of the best performing CSA together with the study of the enantiodiscrimination mechanism is also presented.

## 2. Results and Discussion

**Scheme 2:**
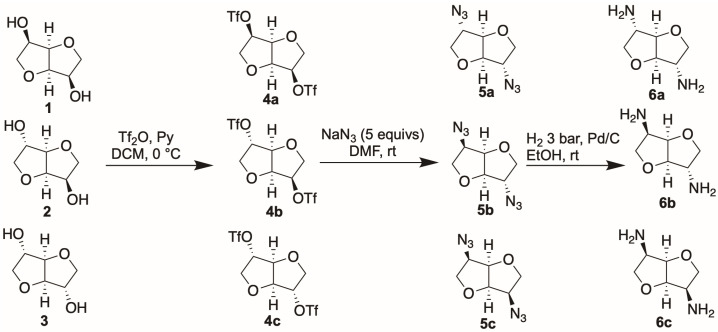
Synthesis of amino derivatives of isohexides **6**.

The synthesis of the amino derivatives of the three isohexides was carried out as described in Scheme 2, starting from commercially available isosorbide and isomannide and from isoidide, which was obtained by Mitsunobu reaction on isomannide with benzoic acid, followed by hydrolysis of the benzoate groups [39].

As reported in Scheme 2, the first step of the synthetic route was the conversion of isohexides **1**–**3** into the corresponding ditriflates. The derivatization was performed by reacting the starting diols **1**–**3** with trifluoromethanesulfonic anhydride in the presence of pyridine at 0 °C. The chemically pure **4** products were obtained in good yields (around 90%), after simple aqueous work-up. Compounds **4** were then converted into diazides **5** via nucleophilic displacement of the triflate groups by sodium azide. As has been well established, the reaction follows a S_N2_ mechanism, thus leading to a complete inversion of configuration at the stereogenic centers bearing the functional groups. As a result, the isomannide-like azide was obtained from the isoidide triflate, and the isomannide triflate was converted into isoidide-like azide, whereas the stereochemistry of the isosorbide core remained unaltered. The reaction was carried out with a high excess of sodium azide in DMF as the solvent at RT, to boost the process toward nucleophilic displacement while minimizing as much as possible the competitive elimination reaction. However, even using these reaction conditions, the formation of the elimination side product was observed, as already reported [39,40]. The extent of the formation of this product strongly depended on the stereochemistry of the triflate. It was observed that when the nucleophile approached from the less hindered convex side of the isohexide, as in the case of endo-triflate, the elimination side reaction did not take place at all, but when the nucleophile approached from the more hindered concave side, as for the exo-triflate, the formation of at least a 30% proportion of elimination product was observed. For this reason, only diazide **5a**, having two exo-azido groups, was obtained as a chemically pure product after aqueous work-up, whereas the other diazides required chromatographic purification. The reaction was totally stereospecific with all substrates, as confirmed by the analysis of the NMR spectra of the three diazides, which showed the presence of only one set of signals for each nucleus in **5b** or for each couple of equivalent nuclei in **5a** and **5c**, so confirming that no epimerization occurred at the stereogenic centers. The diazides were eventually reduced to the corresponding diamines, in good yields, by catalytic hydrogenation.

**Scheme 3:**
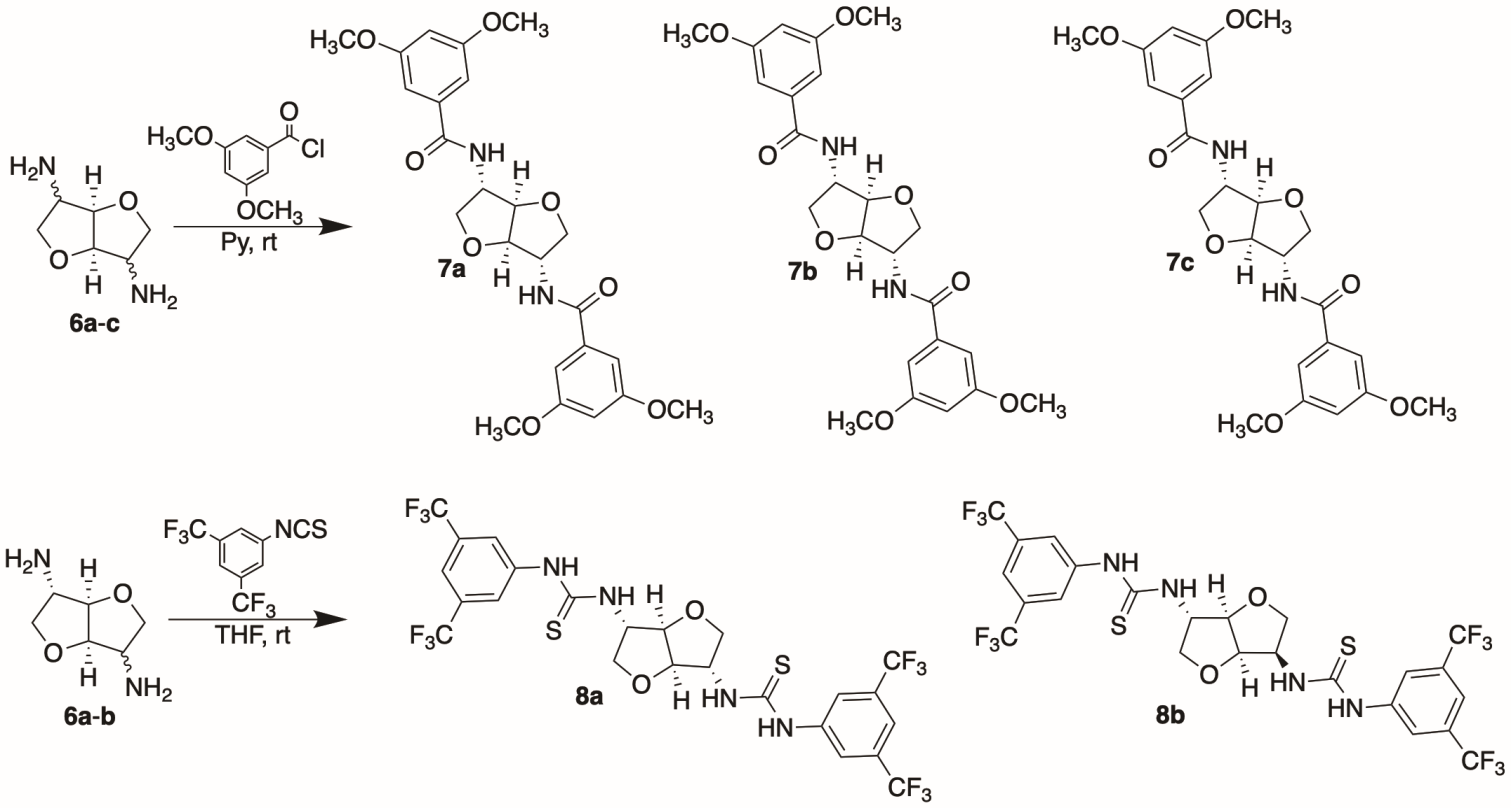
Synthesis of bis-amides **7** and bis–thioureas **8**.

The amino derivatives of isohexides were then converted to arylamides and arylthioureas by reacting diamines **6** with 3,5-dimethoxybenzoyl chloride and 3,5-bis(trifluoromethyl)phenyl isothiocyanate, respectively, under standard reaction conditions (Scheme 3).

**Figure 2:**
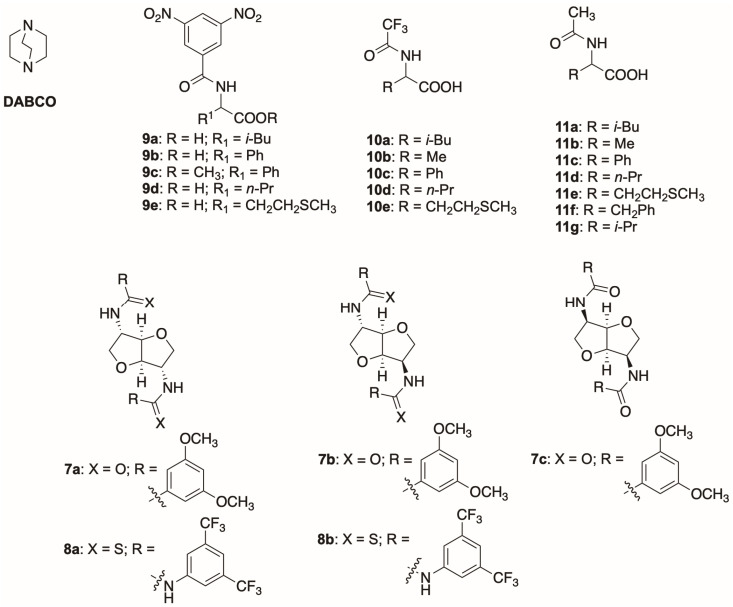
Structures of DABCO, racemic substrates **9a**–**e, 10a**–**e,** and **11a**–**g** and CSAs **7a**–**c** and **8a**–**b**.

The enantiodiscrimination properties of the different amide derivatives **7a**–**c** were assayed in ^1^H NMR experiments towards both racemic ester **9c** and acid **9b** (Figure 2), and the results are presented in Table 1. These substrates were selected because of the presence of the 3,5-dinitrobenzoyl (DNB) group that can not only establish π–π interactions with the electronically complementary aromatic moieties of the isohexide derivatives, which is useful for the enantiodiscrimination, but that also presents some diagnostic signals in a spectral region devoid of proton resonances of the CSAs.

Enantiodiscrimination tests were performed by adding one equivalent of CSA to a 30 mM solution of **9c** in CDCl_3_ as the solvent. In the case of a substrate having underivatized carboxyl functions, 1,4-diazabicyclo[2,2,2]octane (DABCO) was added as solubilizing agent. The enantiodiscrimination efficiency was evaluated by measuring the chemical shift nonequivalence (ΔΔ*δ* = |Δ*δ_R_* − Δ*δ_S_*| = |(*δ_R_* − *δ_f_*) − (*δ_S_* − *δ_f_*)| = |*δ_R_* − *δ_S_*|, ppm; where *δ_R_* and *δ_S_* are the chemical shifts of the (*R*)- and (*S*)-enantiomer of the substrate measured in the mixture and δ*_f_* is the chemical shift of the (*R)-* and (*S*)-enantiomer of the free substrate).

All the diamides were able to induce to some extent nonequivalences of selected protons of **9c** and **9b**, which were slightly higher for the NH proton of both substrates in the presence of **7a** and also for the CH in the case of the mixture containing **7c** and **9b** (Table 1). In that way, some influence of the stereochemistry on the enantiodifferentiating properties of these CSAs was demonstrated. On the basis of this first screening, we concluded that amino derivatives of isohexides can be used to prepare CSAs and that diamide **7a**, having the exo–exo stereochemistry of the arylamide moieties, showed the best enantiodiscrimination properties, mainly towards rac-**9b**, having not derivatized carboxylic function. Endo–endo stereochemistry (**7c**) appeared as the least promising.

Enhanced acidity of thiourea function compared to amide one, together with potentiality of 3,5-bis(trifluoromethyl)phenyl moiety as a booster of enantiodiscriminating capabilities, suggested [25,26,27,28,29,30,31,33] the possibility of changing the aromatic moiety of the CSA preparing thiourea derivatives **8a**–**b** with exo–exo and exo–endo stereochemistry, respectively. Preliminarily, their efficiency was compared using amino acid derivatives with free carboxylic functionalities, on considering the possibility of introducing suitable probes as *N*-3,5-dinitrobenzoyl (*N*-DNB), N-trifluoroacetyl (*N*-TFA) and *N*-acetyl (*N*-Ac) groups for the detection of enantiodifferentiation as in the cases of **9a**–**b**, **10a**, and 11a, respectively.

The first problem we encountered when using **8a**–**b** as CSAs was the high insolubility of **8a** under the initial conditions, which obliged us not only to work with more dilute samples (5 mM), but also to search for a solvent capable of giving homogeneous samples. After accurately screening (Table S1 in Supplementary Materials) for the best conditions for obtaining complete solubility of the chiral solvating agent, conditions were selected as a 5 mM solution of CSA/rac-**9b**/DABCO (1:1:1) in a 3:7 mixture of CD_2_Cl_2_/C_6_D_6_. Although **8b** was highly soluble, the same conditions were used for the sake of comparison. The results reported in Table 2 clearly suggest a higher enantiodiscriminating ability of **8a**–**b** with respect to amide derivatives **7a**–**c**.

In spite of the more dilute conditions (5 mM), in comparison with those conditions selected in the case of CSAs **7a**–**c** (30 mM), higher nonequivalences were measured, which was particularly remarkable in the presence of CSA **8a**. Regarding amino acid derivatives, enantiomers of *N*-Ac ones were those that were most efficiently differentiated, with remarkably high nonequivalences of 0.303 ppm and 0.083 ppm for the NH and acetyl groups, respectively (Table 2).

### 2.1. ^1^H and ^19^F Enantiodiscrimination Experiments

To deal with the enantiodiscriminating versatility of **8a** (Figure 2), we extended our analysis to its 5 mM equimolar mixtures with *N-*DNB (**9d**–**e**, Figure 2), *N*-TFA (**10b**–**e**, Figure 2) and *N*-Ac derivatives (**11b**–**g**, Figure 2) in the presence of one equivalent of DABCO. A CD_2_Cl_2_/C_6_D_6_ (3:7) solvent mixture was once again selected (see Supplementary Materials).

Very high nonequivalences were detected for the NH and acetyl protons of the *N*-Ac derivatives **11a**–**g** (Table 2 and Table 3), with a maximum value of 0.343 ppm for *N*-acetylphenylglycine derivative **11c**. Lower nonequivalences were obtained for *N*-TFA and *N*-DNB derivatives (Table 2 and Table 3).

**Figure 3:**
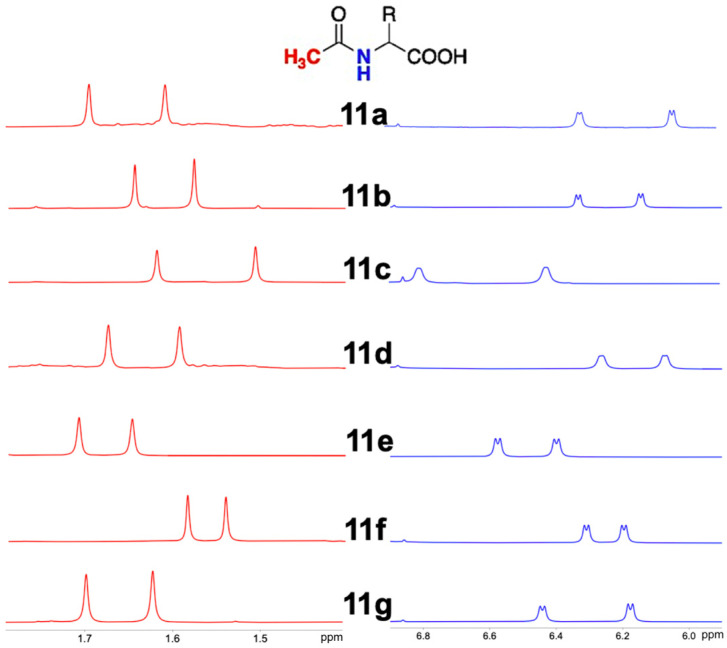
^1^H NMR (600 MHz, 25 °C) spectral regions corresponding to the COCH_3_ (red) and NH (blue) resonances of **11a**–**g** (5 mM) in equimolar mixtures with **8a** and DABCO in CD_2_Cl_2_/C_6_D_6_ (3:7).

Figure 3 shows the relevant spectral regions of the ternary mixtures containing the *N*-acetylamino acids **11b**–**g**. The spectral regions of the ternary mixtures with **9d**–**e** and **10b**–**e** are reported in the Supplementary Materials (Figure S1).

**Figure 4:**
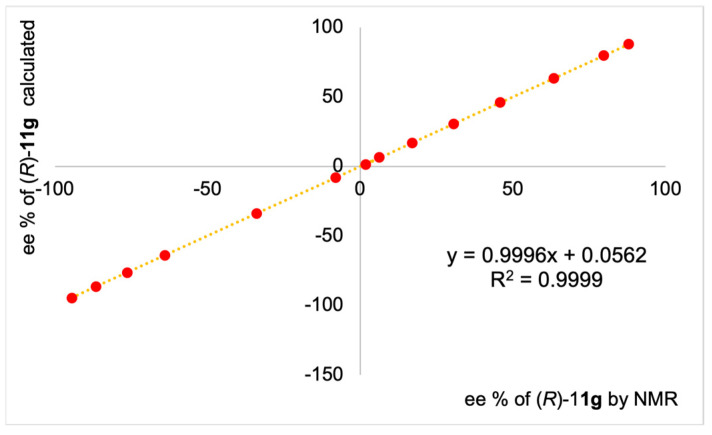
Correlation between the ee% of enantiomerically enriched **11g** (5 mM) in presence of **8a**/DABCO (1:1) in CD_2_Cl_2_/C_6_D_6_ (3:7) calculated from gravimetric data and determined by NMR integration of the NH resonance of the substrate.

Integration of the well separated NH resonances of enantiomeric mixtures of **11a**–**g** allowed us to accurately determine their relative amounts, leading to values of enantiomeric excesses (ee) in very good agreement with gravimetric data (Figure 4, Table S1 in Supplementary Materials), also in samples with very low (6.4%) and very high (−94.7%) ee, as reported in the case of **11g**.

In the development of new chiral solvating agents based on the selected chiral platform, the guiding idea mainly revolves around expanding enantiodiscriminating capabilities by means of the introduction of functional groups with enhanced potential in terms of establishing a tight network of hydrogen bonds, thereby promoting the thermodynamic stabilization of diastereomeric solvates. Therefore, it was imperative to compare the capabilities of the most efficient thiourea-based CSA, i.e., **8a**, with those of the corresponding urea system **12a**. Consequently, we synthesized urea **12a** by reacting diamine **6a** with 3,5-bis(trifluoromethyl)phenyl isocyanate and we assessed its ability to differentiate the proton nuclei of selected enantiomeric substrates, particularly **9a**, **9b**, **10a**, and **11a** (Table 4) For the chiral urea, auxiliary exacerbating solubility issues were found compared to the thiourea counterpart, making it challenging to achieve complete solubilization at a concentration of 5 mM. Therefore, comparative measurements between the two systems were conducted at a concentration of 2 mM, at which **12a** proved to be completely solubilized in the mixtures. In all instances, the urea-based CSA produced significantly lower nonequivalence data compared to the thiourea CSA (Table 4).

### 2.2. ^1^H NMR Configurational Assignment

Only a few CSAs have been so far reported for configurational assignments [31,41,42,43], as CDAs have been preferred in this field since the earliest reports of Mosher in 1963 [44]. As a matter of fact, CDAs may produce higher nonequivalences and originate more conformationally restricted diastereomeric derivatives compared to CSAs. This last feature favors the building of molecular models suited for the rationalization of the correlation between the relative positions of diastereomeric derivatives signals and their absolute configuration. The fixed skeleton of **8a** suggested that it would be worthwhile to explore its use for the configurational assignment of acetyl derivatives of amino acids, in consideration of the remarkable enantiodifferentiation detected in the mixtures **8a**/*N*-Ac-derivative/DABCO. Availability of only one stereoisomer of the CSA required the analysis of enantiomerically enriched samples in order to evaluate the sense of nonequivalence, i.e., the relative positions of enantiomeric signals in enantiomerically enriched samples having known absolute configurations.

**Figure 5:**
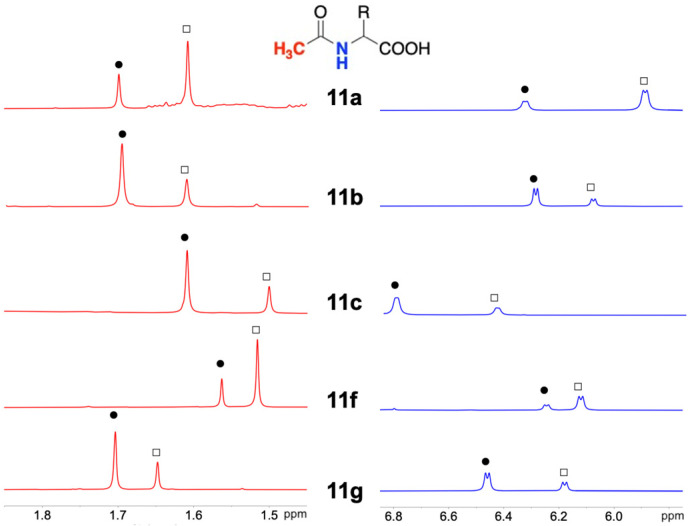
^1^H NMR (600 MHz, 25 °C) spectral regions corresponding to the COCH_3_ (red) and NH (blue) resonances of enantiomerically enriched **11a**–**c**,**f**,**g** in the presence of **8a**/DABCO (1:1) in CD_2_Cl_2_/C_6_D_6_ (3:7). ● and □ indicate (*R*)- and (*S*)-acetylamino acid resonances, respectively.

As shown in Table S2 in Supplementary Materials, for every derivative **11a**–**c**,**f**,**g**, the proton resonances of the NH and Ac groups of the (*R*)-enantiomer were shifted at a higher frequency with respect to those due to the (*S*)-enantiomer in the presence of the CSA (Figure 5).

Therefore, a reproducible correlation between the sense of nonequivalence and the absolute configuration has been obtained, and this correlation can be reliably extended to samples having an unknown absolute configuration, provided that at least a minimum amount of the defect enantiomer is present in the mixture.

### 2.3. Investigation of Chiral Recognition Processes

The chemical bases of the chiral recognition process were investigated in the mixtures containing **8a** and one of each enantiomer of **11g** in the presence of DABCO by 1D or 2D ROESY in order to detect intra- and intermolecular dipolar interactions (Figures S3–S7, S9, and S10 in Supplementary Materials).

The complexation stoichiometries were preliminarily defined using Job’s method in samples having different substrate to CSA ratios while keeping constant the total concentration (Tables S2 and S3 in Supplementary Materials). Graphs reporting the normalized complexation shift of the acetyl protons of the substrate as a function of the molar fraction of **8a** showed a symmetrical bell curve with a well-defined maximum at a 0.5 molar fraction for the diastereomeric complex containing (*S*)-**11g**, according to a 1-to-1 complexation stoichiometry (Figure S2 in Supplementary Materials). Otherwise, a well-defined maximum at the 0.3 molar fraction was obtained in the case of (*R*)-**11g**, demonstrating a 1-to-2 CSA/substrate complexation stoichiometry (Figure S2 in Supplementary Materials).

Very low solubility of the CSA did not allow us to obtain reliable values of the association constants of the two diastereomeric complexes.

Investigation of the stereochemical features of the diastereomeric complexes started from the definition of the conformations of **8a** and **11g** in both ternary mixtures.

**Figure 6:**
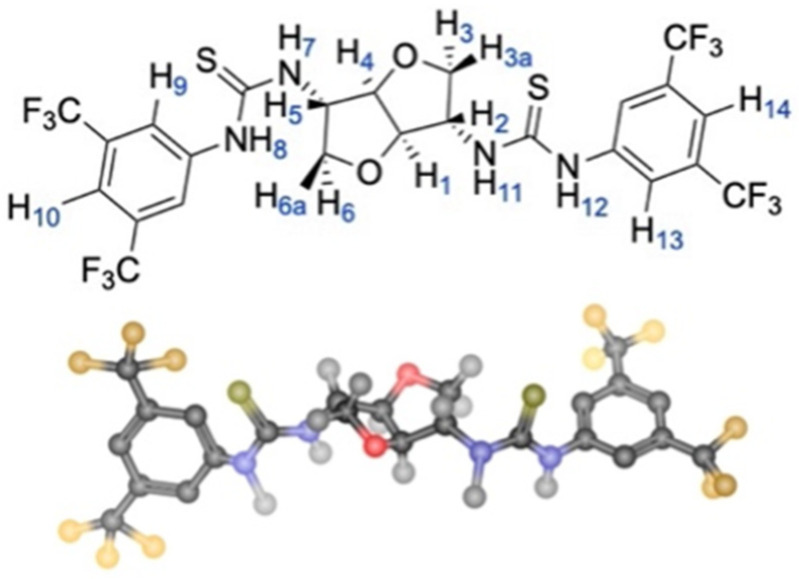
Conformation of **8a** in the ternary mixtures (*R*)- and (*S*)-**11g**/**8a**/DABCO in CD_2_Cl_2_/C_6_D_6_ (3:7) according to NMR data.

Identical intramolecular ROE interactions were detected for **8a** in the two mixtures (Figure S4, Supplementary Materials), indicating that **8a**’s conformation was the same in both solvates. In particular, regarding protons NH-7 and NH-11 (Figure S4 in Supplementary Materials), the intensity of ROE effects at the frequencies of methine protons H_1_ and H_4_ were higher than ROE effects at methylene protons H_3a_/H_6a_. Lower dipolar interactions were detected at H_2_ and H_5_. On this basis, a transoid arrangement of the thiourea protons NH-7/NH-11 and H_2_/H_5_ can be assessed. Intense intra-ROE effects between the amide protons (Figure S5 in Supplementary Materials) supported a cisoid arrangement of these protons in the thiourea fragment (Figure 6) that was further corroborated by the absence of ROE effect between NH-7/NH-11 and protons of 3,5-bis(trifluoromethyl)phenyl groups (Figure S4 in Supplementary Materials).

The conformation of **11g** was also the same in the two mixtures. A transoid arrangement of methine proton H_A_ and amide proton NH-B was suggested by very low-intensity H_A_/NH-B ROE effect compared to the very high-intensity of H_A_/H_D_ ROE (Figures S6 and S7 in Supplementary Materials). The dihedral angle defined by the fragment H_A_-C-N-H_B_ was accordingto data, as a J_HAHB_ of 8.0 Hz and 8.5 Hz was measured for (*R*)- and (*S*)-**11g**, respectively, corresponding [45] to very similar values of the dihedral angles of 147.1° and 150.5° for (*R*)- and (*S*)-**11g**, respectively. The conformation of **11g** in mixture is shown in Figure S8 in Supplementary Materials.

The analysis of intermolecular ROE effects allowed us to impose proximity constraints that were significantly differentiated in the two mixtures.

In particular, very different intermolecular dipolar interactions were originated by H_1_ and H_4_ of **8a**, which were the sole protons of the CSA producing ROE effects with the protons of enantiomeric substrates (Figure S9 in Supplementary Materials). Therefore, both (*R*)-**11g** and (*S*)-**11g** interacted with the convex surface of the CSA, including protons H_1_ and H_4_ of the bicycle and the NH protons of the thiourea moieties. However, H_1_ and H_4_ showed a more intense ROE at the H_A_ proton of (*S*)-**11g** and a less intense effect at its acetyl group (Figure S9 in Supplementary Materials). The reverse was found for the mixture containing (*R*)-**11g**, with the major ROE effect observed at the acetyl protons (Figure S9 in Supplementary Materials).

**Figure 7:**
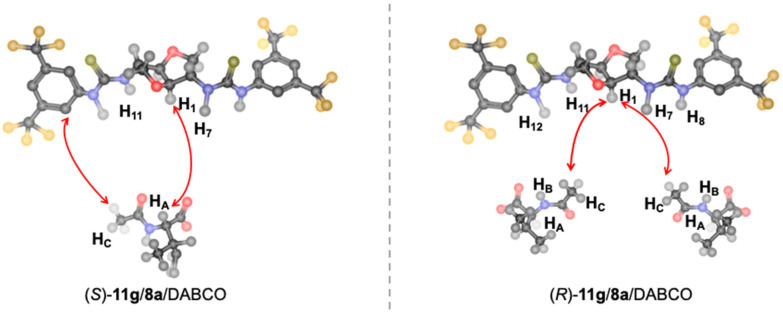
3D schematic representation of diastereomeric complexes (*S*)-**11g**/**8a**/DABCO and (*R*)-**11g**/**8a**/DABCO. The arrows indicate the more relevant ROE effects detected in the corresponding diastereomeric complexes.

Therefore, the (*S*)-enantiomer interacted with the chiral auxiliary, placing the carbonyl functions (acetyl and carboxylate) close to NH-7 and NH-11 groups in a spatial arrangement allowing to place the most sterically demanding group (isopropyl), away from the interaction site, as shown in Figure 7. Accordingly, the acetyl group of (*S*)-**11g** was in spatial proximity to *ortho*-protons of the phenyl moiety of **8a** (Figure S10 in Supplementary Materials). This interaction model was according to the 1-to-1 complexation stoichiometry.

Due to the 1-to-2 CSA to substrate complexation stoichiometry in the mixture containing (*R*)-**11g** and the spatial proximity of the methyl group of the acetyl of (*R*)-**11g** and H_1_ and H_4_ protons of **8a** (Figure S10 in Supplementary Materials), placing its carbonyl functional group far away from the CSA surface, a different interaction model can be hypothesized in which the main stabilizing interaction must necessarily involve the carboxylate group of two amino acid units with each one interacting with one bis-thiourea moiety as depicted in Figure 7.

Interestingly, DABCO protons produced intense ROE contacts with protons of the CSA and protons of each amino acid derivative enantiomer (Figure S4 in Supplementary Materials), supporting its role in the stabilization of the two diastereomeric solvates, which goes beyond its solubilizing effect. DABCO acts as a bridge between the polar groups of the two components.

The mediating role of DABCO became evident through a comparison of its diffusion coefficient (D, m^2^s^−1^) measured by diffusion-ordered spectroscopy experiments in the pure state and its binary mixture containing the chiral auxiliary, along with ternary systems that also included the two enantiomers of the acetyl derivative of valine **11g**. Starting from the initially high value of pure DABCO (D = 21.2 × 10^−10^ m^2^s^−1^), as expected for its small molecular size, the chiral auxiliary itself initiated attractive interactions, resulting in a reduction of the diffusion coefficient of DABCO by 5 units (D = 16.3 × 10^−10^ m^2^s^−1^). An even more pronounced decrease in the diffusion coefficient of DABCO was observed in mixtures (*R*)-**11g**/CSA/DABCO and (*S*)-**11g**/CSA/DABCO, reaching values of 6.7 × 10^−10^ m^2^s^−1^ and 7.4 × 10^−10^ m^2^s^−1^, respectively, which highlights the cooperative function of DABCO in the formation of the two diastereomeric solvates.

Therefore, according to interaction models described above, strongly differentiated chemical environments are felt by the acetyl and amide protons of the two enantiomers leading to their highly differentiated chemical shifts.

## 3. Materials and Methods

### 3.1. Materials

All syntheses of sensitive compounds were realized under dry Argon in flame-dried lab glassware. Reactants and reagents, if not specified, were commercially available and used as received For the synthesis of sensitive compounds, dry CH_2_Cl_2_ and THF obtained using a MB-SPS solvent purification system were used. Pyridine was distilled under inert atmosphere over CaH_2_, and 3,5-bis(trifluoromethyl)phenyl isothiocyanate was distilled under vacuum.

### 3.2. Methods

Analytical thin layer chromatography (TLC) was performed on ALUGRAM Xtra G/UV254 plates (Macherey-Nagel GmbH & Co.KG, Duren, Germany) and detection of compounds was performed with a UV lamp and permanganate or sulfuric vanillin solution. Melting points were measured on a Buchi Melting Point B-545 instrument (BUCHI Italia s.r.l, Cornaredo, Italy), optical rotations were measured on a 1 dm cell at the sodium D line on an Anton Paar MCP 300 Polarimeter or on an Jasco Polarimeter. NMR characterization of the bis-amides **7a**–**c** and corresponding intermediates and the enantiodiscrimination experiments with **7a**–**c** were carried out on a spectrometer operating at 500 or 400 MHz, 126, or 101 MHz for ^1^H and ^13^C nuclei, respectively; the NMR characterization of bis-thioureas **8a**–**b** and *N*-acetylamino acids and the enantiodiscrimination experiments with **8a**–**b** were carried out using a spectrometer operating at 600 MHz, 150 MHz, and 564 MHz for ^1^H, ^13^C, and ^19^F nuclei, respectively. The samples were analyzed in a CDCl_3_, DMSO-d_6_, or methanol-d_4_ solution. ^1^H and ^13^C chemical shifts were referenced to tetramethyl silane (TMS) as the secondary reference standard; ^19^F chemical shifts were referenceed against CFCl_3_ and trifluoro-toluene (**8a**–**8b**) as the external standard with temperature control (25 ± 0.1 and 21 ± 0.1 °C for the spectrometer operating at 600 and 400 or 500 MHz, respectively). A 1 s relaxation delay and 200 increments of 4 transients, each with 2*K*-points, were employed for gCOSY (gradient Correlation SpectroscopY) and TOCSY (Total COrrelation SpectroscopY) experiments. The mixing time for TOCSY maps was 80 ms. The 2D-ROESY (Rotating-frame Overhauser Enhancement SpectroscopY) experiments were carried out with a relaxion time of 1 s, a mixing time of 0.3 s, and 128 increments of 8 transients, with 2*K*-points. For 1D-ROESY spectra, a selective inversion pulse was employed with transients ranging from 512 to 1024, a relaxion delay of 1 s, and a mixing time of 0.5 s. The gHSQC (gradient Heteronuclear Single Quantum Coherence) and the gHMBC (gradient Heteronuclear Multiple Bond Correlation) spectra were recorded with a relaxion time of 1.2 s and 128–200 increments with 16–32 transients, each with 2*K*-points. The gHMBC experiments were optimized for a long-range coupling constant of 8 Hz. For DOSY (Diffusion-Ordered Spectroscopy) experiments, a stimulated echo sequence with self-compensating gradient schemes and 64 K data points was used. Typically, g was varied in 20 steps (2–32 transients each), and Δ and δ were optimized to obtain an approximately 90–95% decrease in resonance intensity at the largest gradient amplitude. Baselines of arrayed spectra were corrected prior to processing the data. After data acquisition, each FID was apodized with 1.0 Hz line broadening and then Fourier transformed. DOSY macro was used for data processing. The assignments of ^1^H NMR and ^13^C NMR chemical shifts for **8a**–**b**, reported below, are shown in Figures SI32, SI33, SI34, and SI35 in Supplementary Materials; the NMR data were described using the following abbreviations: s—singlet, d—doublet, dt—double doublet, ddd—double double douplet, t—triplet, q—quartet, and m—multiplet.

### 3.3. General Procedures for the Synthesis of the bis-Amides ***7a**–**c***

To a solution of diamine (1.0 mmol) in dry pyridine (5 mL), 3,5-dimethoxybenzoyl chloride (421 mg, 2.1 mmol) was added. The suspension was stirred for 6 h at room temperature and the reaction was monitored by TLC analysis (CH_2_Cl_2_/Acetone/HCOOH 85:15:1). Then, water (10 mL) was added and the mixture was extracted with CH_2_Cl_2_ (3 × 10 mL). The organic phases were combined and washed sequentially with 10% aqueous HCl (10 mL), H_2_O (10 mL), saturated NaHCO_3_ (10 mL), and again with H_2_O (10 mL). The organic phase was dried over anhydrous Na_2_SO_4_. The solvent was removed under reduced pressure to give the crude product.

#### 3.3.1. *N*,*N*′-((3*S*,3a*R*,6*S*,6a*R*)-Hexahydrofuro[3,2-b]furan-3,6-diyl)bis(3,5-dimethoxybenzamide), (**7a**)

The product was purified by flash chromatography (silica gel, CH_2_Cl_2_/Acetone/HCOOH 85:15:1) to give 189 mg (0.4 mmol, 39%) of a pure white solid. Mp = 75–77 °C. ${\left[\alpha \right]}_{D}^{20}\;$= +50.2 (c = 0.55; Acetone); ^1^H NMR (500 MHz, CDCl_3_, 21 °C, TMS, ppm) δ: 6.84 (d, *J* = 2.3 Hz, 4H), 6.57 (t, *J* = 2.3 Hz, 2H), 6.17 (d, *J* = 7.3 Hz, 2H), 4.71 (s, 2H), 4.62 (ddd, *J* = 6.9 Hz, *J* = 4.6 Hz, *J* = 2.0 Hz, 2H), 4.10–4.04 (m, 2H), 3.92 (dd, *J* = 9.9 Hz, *J* = 2.0 Hz, 2H), 3.80 (s, 12H). ^13^C{^1^H} NMR (101 MHz, CDCl_3_, 21 °C, TMS, ppm) δ: 167.1, 161.1, 136.0, 105.0, 104.0, 86.4, 72.5, 57.1, and 55.7.

#### 3.3.2. *N,N′*-((3*R*,3a*R*,6*S*,6a*R*)-Hexahydrofuro[3,2-b]furan-3,6-diyl)bis(3,5-dimethoxybenzamide), (**7b**)

The product was purified by flash chromatography (silica gel, CH_2_Cl_2_/Acetone/HCOOH 85:15:1) to give 142 mg (0.3 mmol, 32%) of a white solid. Mp = 192–193 °C. ${\left[\alpha \right]}_{D}^{25}$ = +51.2 (c = 0.46; CHCl_3_). ^1^H NMR (500 MHz, CDCl_3_, 21 °C, TMS, ppm) δ: 6.90 (d, *J* = 2.2 Hz, 2H), 6.85 (d, *J* = 2.2 Hz, 2H), 6.64 (d, *J* = 7.2 Hz, 1H), 6.58 (q, *J* = 2.5 Hz, 2H),), 6.19 (d, *J* = 7.3 Hz, 1H), 4.67 (m, 4H), 4.30 (dd, *J* = 8.8 Hz, *J* = 7.2 Hz, 1H), 4.10 (dd, *J* = 9.8 Hz, *J* = 4.7 Hz, 1H), 4.00–3.94 (m, 1H), 3.82 (s, 12H), 3.51 (dd, *J* = 16.0 Hz, *J* = 8.5 Hz, 1H). ^13^C{^1^H} NMR (126 MHz, CDCl_3_, 21 °C, TMS, ppm) δ 167.4, 167.1, 161.1, 161.0, 136.1, 136.0, 105.1, 105.0, 103.9, 103.7, 87.0, 81.6, 73.5, 71.7, 57.9, 55.7, and 53.3.

#### 3.3.3. *N,N′*-((3*R*,3a*R*,6*R*,6a*R*)-Hexahydrofuro[3,2-b]furan-3,6-diyl)bis(3,5-dimethoxybenzamide), (**7c**)

The product was purified by flash chromatography (silica gel, CH_2_Cl_2_/Acetone 9:1) to give 142 mg (0.3 mmol, 30%) of a white foamy solid. Mp = 168 °C. ${\left[\alpha \right]}_{D}^{25}$ = +119.4 (c = 0.69; CHCl_3_). ^1^H NMR (400 MHz, CDCl_3_, 21 °C, TMS, ppm) δ: 6.90 (d, *J* = 2.3 Hz, 4H), 6.71 (d, *J* = 7.1 Hz, 2H), 6.58 (t, *J* = 2.3 Hz, 2H), 4.78–4.67 (m, 4H), 4.40 (t, *J* = 7.9 Hz, 2H), 3.82 (s, 12H), 3.52–3.46 (m, 2H). ^13^C{^1^H} NMR (126 MHz, CDCl_3_, 21 °C, TMS, ppm) δ: 167.4, 161.0, 131.9, 105.2, 103.8, 82.0, 72.6, 55.7, and 54.0.

### 3.4. General Procedures for the Synthesis of the bis-Thioureas ***8a**–**b***

To a solution of diamine (154.0 mg, 1.1 mmol) in dry THF (10 mL), 3,5-bis(trifluoromethyl)phenyl isothiocyanate 370 μL (1.9 mmol) was added. The mixture was stirred overnight at room temperature and the reaction was monitored by TLC analysis (CHCl_3_/MeOH 9:1). The solvent was removed under reduced pressure to obtain the crude product.

#### 3.4.1. 1,1′-((3*S*,3a*R*,6*S*,6a*R*)-Hexahydrofuro[3,2-b]furan-3,6-diyl)bis(3-(3,5-bis(trifluoromethyl)phenyl)thiourea), (**8a**)

The solid was recrystallized by Acetone-Hexane obtaining 511 mg (0.8 mmol, 70%) of a white powder. Mp = 173–175 °C. ${\left[\alpha \right]}_{D}^{20}$ = −22.9 (c = 0.35; MeOH). ^1^H NMR (600 MHz, [D_6_]DMSO, TMS, 25 °C, ppm) δ: 9.94 (s, 2H), 8.57 (s, 2H), 8.22 (s, 4H), 7.76 (s, 2H), 4.69 (s, 2H), 4.64 (s, 2H), 4.00 (dd, *J* = 9.8 Hz, *J* = 4.8 Hz, 2H), 3.84 (d, *J* = 9.8 Hz, 2H). ^13^C{^1^H} NMR (150 MHz, [D_6_]DMSO, TMS, 25 °C, ppm) δ: 180.6 (*C*=S), 141.6 (*C*-Ar), 130.0 (q, *J* =33.0 Hz, *C*-CF_3_), 123.7 (q, *J* = 272.9 Hz, *C*F_3_), 122.2 (*C*-Ar), 116.3 (*C*-Ar), 85.5 (*C*H), 71.3 (*C*H_2_), 60.3 (*C*H). ^19^F NMR (564 MHz, [D_6_]DMSO, trifluoro-toluene, ppm) δ: −0.47.

#### 3.4.2. 1,1′-((3*R*,3a*S*,6*S*,6a*R*)-Hexahydrofuro[3,2-b]furan-3,6-diyl)bis(3-(3,5-bis(trifluoromethyl)phenyl)thiourea), (**8b**)

The product was purified by chromatography (silica gel, CHCl_3_/Acetone 8:2) to give 479.1 mg (0.7 mmol, 66%) of a yellow powder. Mp = 92–97 °C. ${\left[\alpha \right]}_{D}^{20}$ = + 297 (c = 0.01; EtOH). ^1^H NMR (600 MHz, [D6]DMSO, TMS, 25 °C, ppm) δ: 10.45 (s, 1H), 9.98 (s, 1H), 8.57 (s, 1H), 8.29 (s, 2H), 8.22 (s, 2H), 8.11 (d, *J* = 7.2 Hz, 1H), 7.74 (s), 4.80 (q, *J* = 7.3 Hz, 1H), 4.73 (m, 2H), 4.64 (d, *J* = 4.0 Hz, 1H), 4.13 (t, *J* = 8.0 Hz, 1H), 4.03 (dd, *J* = 9.7 Hz, *J* = 4.7 Hz, 1H), 3.92 (m, 1H), 3.47 (t, *J* = 8.7 Hz, 1H). ^13^C{^1^H} NMR (150 MHz, [D_6_]DMSO, TMS, 25 °C, ppm) δ: 181.2 (*C*=S), 181.0 (*C*=S), 142.2 (*C*-Ar), 130.1 (m, *C*-CF_3_), 123.7 (q, J = 271.4 Hz, *C*F_3_), 122.7 (*C*-Ar), 122.1 (*C*-Ar), 116.8 (*C*-Ar), 86.7 (*C*H), 81.2 (*C*H), 73.1 (*C*H_2_), 70.3 (*C*H_2_), 61.7 (*C*H), 57.5 (*C*H). ^19^F NMR (564 MHz, [D_6_]DMSO, trifluoro-toluene, ppm) δ: −0.46 and −0.49.

### 3.5. 1,1′-((3S,3aR,6S,6aR)-Hexahydrofuro[3,2-b]furan-3,6-diyl)bis(3-(3,5-bis(trifluoromethyl)phenyl)urea), (***12a***)

To a solution of diamine (280 mg, 1.94 mmol) in dry THF (18 mL), 3,5-bis(trifluoromethyl)phenyl isocyanate (740 μL, 2.2 mmol) was added. The mixture was stirred overnight at room temperature and the reaction was monitored by TLC analysis (CHCl_3_/MeOH 9:1). The solvent was removed under reduced pressure to obtain the product (1.74 mmol, 90%). ^1^H NMR (600 MHz, [D_6_]DMSO, TMS, 25 °C, ppm) δ: 9.07 (s, 2H), 8.16 (s, 4H), 7.54 (s, 2H), 6.89 (d, *J* = 6.8 Hz, 2H), 4.50 (s, 2H), 4.08 (m, 2H), 3.89 (dd, *J* = 9.5 Hz, *J* = 5.1 Hz, 2H), 3.69 (dd, *J* = 9.5 Hz, *J* = 1.8 Hz, 2H). ^13^C{^1^H} NMR (150 MHz, [D_6_]DMSO, TMS, 25 °C, ppm) δ: 154.9 (*C*=O), 141.6 (*C*-Ar), 131.2 (q, *J* =32.8 Hz, *C*-CF_3_), 123.8 (q, *J* = 273.0 Hz, *C*F_3_), 118.1 (*C*-Ar), 114.3 (*C*-Ar), 86.6 (*C*H), 71.2 (*C*H_2_), 56.8 (*C*H).

## 4. Conclusions

New U-, N-, and W-shaped arylamides and arylthioureas CSAs **7a**–**c** and **8a**–**b** were easily synthesized starting with commercially available isomannide and isosorbide. The stereospecific interconversion of the hydroxyls of the two natural isohexides allowed us to obtain the three amino derivatives having isomannide, isosorbide, and isoidide stereochemistry, which were easily transformed into the corresponding arylamides, and arylthioureas. In view of the biological relevance of amino acids, their *N*-DNB, *N*-TFA, and *N*-Ac derivatives were probed as chiral substrates in the enantiodiscrimination experiments, highlighting the finding that bis-thiourea CSA **8a** with 3,5-bis(trifluoromethyl)phenyl moieties in an exo–exo stereochemistry was endowed with remarkable enantiodiscriminating power towards *N*-acetylamino acids, surpassing that of the corresponding urea derivative **12a**. The nature of the probe signals (doublets for NH and singlets for Ac) of the chiral substrates allowed us to also attain accurate determinations of enantiomeric compositions in diluted mixtures with very high or very low enantiomeric excesses. Importantly, **8a** constitutes one of the few cases of the use of CSAs for configurational assessments. It is likely that the flexible lateral arms of the CSA in an open conformation can act in synergy to grab a single substrate unit. Alternatively, they can act independently while grabbing two of them in response to steric-repulsive effects. In that way, an efficient enantiomeric differentiation can be attained. As a matter of fact, alternative exo–endo stereochemistry, in which thiourea moieties cannot cooperate in the interaction with the chiral substrates, produces lower enantiomer differentiations in the NMR spectra. Therefore, anisohexide skeleton constitutes a versatile chiral platform for the design of new chiral auxiliaries bearing different kinds of functional groups to be applied to the enantiodiscrimination of different classes of chiral substrates.

## Figures and Tables

**Table 1:** ^1^H NMR (500 MHz, CDCl_3_, 25 °C) nonequivalences (ΔΔ*δ*, ppm) of selected protons of racemic **9b**–**c** (30 mM) in equimolar mixtures with **7a**–**c**.

		ΔΔ*δ*				
CSA	Substrate	NH	CH	*para*-DNB	*ortho*-DNB	CO_2_Me
**7a**	**9c**	0.013	0.002	0	0.002	0.003
**7b**	**9c**	0	0.006	0.001	0.002	0
**7c**	**9c**	0.008	0.005	0	0.002	0
**7a**	**9b** **^1^**	0.013	0.007	0.005	0.013	
**7b**	**9b** **^1^**	0	0.003	0	0.001	
**7c**	**9b** **^1^**	0	0.013	0	0	

^1^ In the presence of 1 equivalent of DABCO.

**Table 2:** ^1^H NMR (600 MHz, 25 °C) nonequivalences (ΔΔ*δ*, ppm) of selected protons of **9a**–**b**, **10a**, and **11a** (5 mM) in equimolar mixtures with **8a**–**b** and DABCO in CD_2_Cl_2_/C_6_D_6_ (3:7).

		ΔΔ*δ*				
CSA	Substrate	NH	*para*-DNB	*ortho*-DNB	CF_3_	Ac
**8a**	**9a**	0	0	0.077		
**8b**	**9a**	0	0	0.038		
**8a**	**9b**	0.109	0	0.019		
**8b**	**9b**	0	0	0.035		
**8a**	**10a**	0.053			0.010	
**8b**	**10a**	0			0.020	
**8a**	**11a**	0.303				0.083
**8b**	**11a**	0.056				0.040

**Table 3:** ^1^H (600 MHz, 25 °C) and ^19^F (564 MHz, 25 °C) NMR nonequivalences (ΔΔ*δ*, ppm) for **9d**–**e**, **10b**–**e**, and **11b**–**g** (5 mM) in equimolar mixtures with **8a** and DABCO in CD_2_Cl_2_/C_6_D_6_ (3:7).

		ΔΔ*δ*			
Substrate	NH	*para*-DNB	*ortho*-DNB	CF_3_	Ac
**9d**	0	0	0.029		
**9e**	0	0.021	0.027		
**10b**	0.039			0.016	
**10c**	0.023			0.016	
**10d**	0.045			0.013	
**10e**	0			0.010	
**11b**	0.184				0.066
**11c**	0.343				0.107
**11d**	0.189				0.078
**11e**	0.159				0.069
**11f**	0.104				0.042
**11g**	0.257				0.076

**Table 4:** ^1^H NMR (600 MHz, 25 °C) nonequivalences (ΔΔ*δ*, ppm) of selected protons of racemic **9a**-**b**, **10a**, and **11a** (2 mM) in equimolar mixtures with **8a** or **12a** and DABCO in CD_2_Cl_2_/C_6_D_6_ 3:7.

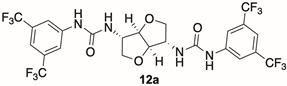						
		ΔΔ*δ*				
CSA	Substrate	NH	*para*-DNB	*ortho*-DNB	Ac	CF_3_
**8a**	**9a**	0.107	0.003	0.044		
**12a**	**9a**	0.040	0	0.018		
**8a**	**9b**	0.086	0.008	0.021		0
**12a**	**9b**	0.016	0	0		
**8a**	**10a**	0.055				0.009
**12a**	**10a**	0				0
**8a**	**11a**	0.419			0.079	
**12a**	**11a**	0.163			0.016	

## Data Availability

Data are contained within the article and Supplementary Materials.

## Supplementary Materials

The following supporting information can be downloaded at https://www.mdpi.com/article/10.3390/molecules29061307/s1. Figure SI1, ^1^H NMR spectrum of diphenyl (3*S*,3a*R*,6*S*,6a*R*)-hexahydrofuro[3,2-b]furan-3,6-dicarboxylate; Figure SI2, ^13^C{^1^H} spectrum of diphenyl (3*S*,3a*R*,6*S*,6a*R*)-hexahydrofuro[3,2-b]furan-3,6-dicarboxylate; Figure SI3, ^1^H NMR spectrum of **3**; Figure SI4, ^13^C{^1^H} NMR spectrum of **3**; Figure SI5, ^1^H NMR spectrum of **4a**; Figure SI6, ^13^C{^1^H} NMR spectrum of **4a**; Figure SI7, ^19^F NMR spectrum of **4a**; Figure SI8, ^1^H NMR spectrum of **4b**; Figure SI9, ^13^C{^1^H} NMR spectrum of **4b**; Figure SI10, ^19^F NMR spectrum of **4b**; Figure SI11, ^1^H NMR spectrum of **4c**; Figure SI12, ^13^C{^1^H} NMR spectrum of **4c**; Figure SI13, ^19^F NMR spectrum of **4c**; Figure SI14, ^1^H NMR spectrum of **5a**; Figure SI15, ^13^C{^1^H} NMR spectrum of **5a**; Figure SI16, ^1^H NMR spectrum of **5b**; Figure SI17, ^13^C{^1^H} NMR spectrum of **5b**; Figure SI18, ^1^H NMR spectrum of **5c**; Figure SI19 ^13^C{^1^H} NMR spectrum of **5c**; Figure SI20, ^1^H NMR spectrum of **6a**; Figure SI21, ^13^C{^1^H} NMR spectrum of **6a**; Figure SI22, ^1^H NMR spectrum of **6b**; Figure SI23, ^13^C{^1^H} NMR spectrum of **6b**; Figure SI24, ^1^H NMR spectrum of **6c**; Figure SI25, ^13^C{^1^H} NMR spectrum of **6c**; Figure SI26, ^1^H NMR spectrum of **7a**, Figure SI27, ^13^C{^1^H} NMR spectrum of 7a; Figure SI28, ^1^H NMR spectrum of **7b**; Figure SI29, ^13^C{^1^H} NMR spectrum of **7b**; Figure SI30, ^1^H NMR spectrum of **7c**; Figure SI31, ^13^C{^1^H} NMR spectrum of **7c**; Figure SI32, ^1^H NMR spectrum of **8a**; Figure SI33, ^13^C{^1^H} NMR spectrum of **8a**, Figure SI34, ^1^H NMR spectrum of **8b**; Figure SI35, ^13^C{^1^H} NMR spectrum of **8b**; Table SI1, Nonequivalence for racemic **11a** in equimolar mixtures **11a**/**8a**/DABCO in different solvents; Figure S1, Nonequivalences of **9c**–**d** and **10b**–**e** in equimolar mixtures with **8a** and DABCO; Figure S2, Complexation stoichiometry of (R)-11g/8a/DABCO (left) and (S)-11g/8a/DABCO (right) based on the 1H resonances of the acetyl group of 11g; Table S1, Enantiomeric purity determination; Table S2, Complexation shift of **11a**–**c**,**11f**, **11g** in equimolar mixtures with **8a** and DABCO; Table S3, Chemical shift of Ac protons of (*S*)-**11g** in the mixture (*S*)-**11g/8a**/DABCO; Table S4, Chemical shift of Ac protons of (*R*)-**11g** in the mixture (*R*)-**11g/8a**/DABCO; Figure S3, ^1^H NMR spectra of the equimolar (5 mM) mixtures (*R*)-**11g**/**8a**/DABCO (*S*)-**11g**/ **8a**/DABCO; Figure S4, 2D ROESY maps of the equimolar (5 mM) mixtures (*R*)-**11g**/**8a**/DABCO (*S*)-**11g**/ **8a**/DABCO; Figure S5, 1D ROESY spectra of NH-8/NH-12 of (*R*)-**11g** (5 mM) in the equimolar (*R*)-**11g**/DABCO/**8a**; Figure S6, 1D ROESY spectra of H_A_ of (*R*)-**11g** (5 mM) in the mixture (*R*)-**11g**/DABCO/**8a**; Figure S7, 1D ROESY spectra of the proton H_B_ of (*R*)-**11g** (5 mM) in the mixture (*R*)-**11g**/DABCO/**8a**; Figure S8, 3D representation of (*R*)-**11g**; Figure S9, 1D ROESY spectra of H_1_ and H_4_ of (*R*)-/(*S*)-**11g** (5 mM) in the mixture (*R*)-/(*S*)-**11g**/DABCO/**8a**; Figure S10, 1D ROESY spectra of H_c_ of (*R*)-/(*S*)-**11g** (5 mM) in the mixture (*R*)-/(*S*)-**11g**/DABCO/**8a**. Refs [46,47,48,49,50,51] are cited in Supplementary Materials.
