# Supplementary material for: Isohexide-Based Tunable Chiral Platforms as Amide- and Thiourea-Chiral Solvating Agents for the NMR Enantiodiscrimination of Derivatized Amino Acids
Source: Molecules. 2024 Mar 15;29(6):1307. doi: 10.3390/molecules29061307 (PMC10974320; doi:10.3390/molecules29061307)
Supplement: Supplementary file 1 [file molecules-29-01307-s001.zip › molecules-2905499-supplementary.pdf]

Supplementary Materials

Isohexide-based Tunable Chiral Platforms as Amide and Thiourea Chiral Solvating Agents for the NMR Enantiodiscrimination of Derivatized Amino Acids.

Federica Cefalì,[a] Anna Iuliano,*[a] Federica Balzano,*[a] Gloria Uccello-Barretta,[a] Valerio Zullo[a] and Carlo Baldassari[a]

[a] Dipartimento di Chimica e Chimica Industriale, Via Giuseppe Moruzzi 13, 56124, Pisa, Italy.

Table of contents

Synthesis of isoidide
Diphenyl (3*S*,3a*R*,6*S*,6a*R*)-hexahydrofuro[3,2-b]furan-3,6-dicarboxylate
(3*R*,3*aS*,6*R*,6*aS*)-2,3,3*a*,5,6,6*a*-hexahydrofuro[3,2-b]furan-3,6-diol

General procedure for the synthesis of compounds 4
(3*R*,3a*S*,6*R*,6a*S*)-hexahydrofuro[3,2-b]furan-3,6-diylbis(trifluoromethanesulfonate)
(3*R*,3a*S*,6*S*,6a*S*)-hexahydrofuro[3,2-b]furan-3,6-diylbis(trifluoromethanesulfonate)
(3*S*,3aS,6*S*,6a*S*)-hexahydrofuro[3,2-b]furan-3,6-diylbis(trifluoromethanesulfonate)

General procedure for the synthesis of compounds 5
(3*S*,3a*R*,6*S*,6a*R*)-3,6-diazidohexahydrofuro[3,2-b]furan
(3*R*,3a*R*,6*S*,6a*R*)-3,6-diazidohexahydrofuro[3,2-b]furan
(3*R*,3a*R*,6*R*,6a*R*)-3,6-diazidohexahydrofuro[3,2-b]furan

General procedure for the synthesis of compounds 6
(3*S*,3a*R*,6*S*,6a*R*)-hexahydrofuro[3,2-b]furan-3,6-diamine
(3*R*,3a*R*,6*S*,6a*R*)-hexahydrofuro[3,2-b]furan-3,6-diamine
(3*R*,3a*R*,6*R*,6a*R*)-hexahydrofuro[3,2-b]furan-3,6-diamine

Synthesis of N-acetylamino acids

^{1}H and ^{13}C{^{1}H} NMR spectra
Figure SI1: ^{1}H NMR spectrum of diphenyl (3*S*,3a*R*,6*S*,6a*R*)-hexahydrofuro[3,2-b]furan-3,6-dicarboxylate.
Figure SI2: ^{13}C{^{1}H} NMR spectrum of diphenyl (3*S*,3a*R*,6*S*,6a*R*)-hexahydrofuro[3,2-b]furan-3,6-dicarboxylate.
Figure SI3: ^{1}H NMR spectrum of **3**
Figure SI4: ^{13}C{^{1}H} NMR spectrum of **3**
Figure SI5: ^{1}H NMR spectrum of **4a**
Figure SI6: ^{13}C{^{1}H} NMR spectrum of **4a**
Figure SI7: ^{19}F NMR spectrum of **4a**
Figure SI8: ^{1}H NMR spectrum of **4b**
Figure SI9: ^{13}C{^{1}H} NMR spectrum of **4b**
Figure SI10: ^{19}F NMR spectrum of **4b**
Figure SI11: ^{1}H NMR spectrum of **4c**
Figure SI12: ^{13}C{^{1}H} NMR spectrum of **4c**
Figure SI13. ^{19}F NMR spectrum of **4c**
Figure SI14: ^{1}H NMR spectrum of **5a**
Figure SI15: ^{13}C{^{1}H} NMR spectrum of **5a**
Figure SI16: ^{1}H NMR spectrum of **5b**
Figure SI17: ^{13}C{^{1}H} NMR spectrum of **5b**
Figure SI18: ^{1}H NMR spectrum of **5c**

Figure SI19 $^{13}C\{^{1}H\}$ NMR spectrum of **5c**
Figure SI20: ^{1}H NMR spectrum of **6a**
Figure SI21: $^{13}C\{^{1}H\}$ NMR spectrum of **6a**
Figure SI22: ^{1}H NMR spectrum of **6b**
Figure SI23 $^{13}C\{^{1}H\}$ NMR spectrum of **6b**
Figure SI24. ^{1}H NMR spectrum of **6c**
Figure SI25: $^{13}C\{^{1}H\}$ NMR spectrum of **6c**
Figure SI26: ^{1}H NMR spectrum of **7a**
Figure SI27: $^{13}C\{^{1}H\}$ NMR spectrum of **7a**
Figure SI28: ^{1}H NMR spectrum of **7b**
Figure SI29: $^{13}C\{^{1}H\}$ NMR spectrum of **7b**
Figure SI30: ^{1}H NMR spectrum of **7c**
Figure SI31:$^{13}C\{^{1}H\}$ NMR spectrum of **7c**
Figure SI32: ^{1}H NMR spectrum of **8a**
Figure SI33: $^{13}C\{^{1}H\}$ NMR spectrum of **8a**
Figure SI34: ^{1}H NMR spectrum of **8b**
Figure SI35: $^{13}C\{^{1}H\}$ NMR spectrum of **8b**

Optimization of the experimental condition using in NMR enantiodiscrimination experiments

Table SI1. Nonequivalence for racemic **11a** in equimolar mixtures **11a**/**8a**/DABCO in different solvents.

^{1}H and ^{19}F Enantiodiscrimination Experiments

Figure S1: Nonequivalences of **9d**-**e** and **10b**-**e** in equimolar mixtures with **8a** and DABCO
Table S1. Enantiomeric purity determination

^{1}H NMR configurational assignment

Table S2. Complexation shift of **11a**-**c**,**11f**, **11g** in equimolar mixtures with **8a** and DABCO

Investigation recognition processes

Table S3. Chemical shift of Ac protons of (*S*)-**11g** in the equimolar mixture (*S*)-**11g**/**8a**/DABCO
Table S4. Chemical shift of Ac protons of (*R*)-**11g** in the equimolar mixture (*R*)-**11g**/**8a**/DABCO
Figure S3. ^{1}H NMR spectra of the equimolar (5mM) mixtures (*R*)-**11g**/**8a**/DABCO and (*S*)-**11g**/ **8a**/DABCO
Figure S4. 2D ROESY maps of the equimolar (5mM) mixtures (*R*)-**11g**/**8a**/DABCO and (*S*)-**11g**/ **8a**/DABCO
Figure S5. 1D ROESY spectrum of NH-8/NH-12 of (*R*)-**11g** (5mM) in the equimolar mixture (*R*)-**11g**/DABCO/**8a**.
Figure S6. 1D ROESY spectrum of H_A of (*R*)-**11g** (5mM) in the equimolar mixture (*R*)-**11g**/DABCO/**8a**.
Figure S7. 1D ROESY spectrum of the proton H_B of (*R*)-**11g** (5mM) in the equimolar mixture (*R*)-**11g**/DABCO/**8a**.
Figure S8. 3D representation of (*R*)-**11g**.
Figure S9. 1D ROESY spectra of H_1 and H_4 of (*R*)-/(*S*)-**11g** (5mM) in the equimolar mixture (*R*)-/(*S*)-**11g**/DABCO/**8a**.
Figure S10. 1D ROESY spectra of H_c of (*R*)-/(*S*)-**11g** (5mM) in the equimolar mixture (*R*)-/(*S*)-**11g**/DABCO/**8a**.

Synthesis of isoidide 3[1]

Diphenyl (3*S*,3a*R*,6*S*,6a*R*)-hexahydrofuro[3,2-b]furan-3,6-dicarboxylate.

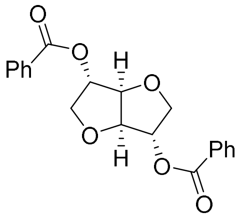

Isomannide (736 mg, 5.0 mmol) and triphenylphosphine (2.91 g, 11.1 mmol) were dissolved in dry THF (20 mL), then a solution of benzoic acid (1.36 g, 11.2 mmol) and DEAD (5.0 mL of a 40% toluene solution, 10.9 mmol) in dry THF (20 mL) was dropwise added. The mixture was stirred at room temperature and the reaction was monitored by TLC analysis (Hexane:Ethyl acetate 8:2). After 6h triphenylphosphine (263.6 mg, 1.0 mmol), benzoic acid (124 mg, 1.0 mmol) of and DEAD (450 µL of 40% solution in toluene, 1.0 mmol) were added. The reaction mixture was stirred overnight at room temperature and total conversion was observed. The solvent was removed under reduced pressure and the crude product was purified by flash chromatography (silica gel, Hexane: Ethyl acetate 8:2) to give 1.6190 g (4.5 mmol, 90%) of a white solid. Mp = 106-108 °C. $[\alpha]_D^{20}$ = +141.6 (c = 1.13; $CHCl_3$). ^1H NMR (500 MHz, $CDCl_3$, TMS, 21 °C, ppm) δ: 8.03 (d, *J* = 8.4 Hz, 4H), 7.57 (t, *J* = 7.5 Hz, 2H), 7.44 (t, *J* = 7.8 Hz, 4H), 5.50 (s, 2H), 4.87 (s, 2H), 4.17 – 4.07 (m, 4H). $^{13}C\{^1H\}$ NMR (126 MHz, $CDCl_3$, TMS, 21 °C, ppm) δ: 165.6, 133.6, 129.9, 129.5, 128.6, 85.7, 78.1, 72.8.

(3*R*,3*aS*,6*R*,6*aS*)-2,3,3*a*,5,6,6*a*-hexahydrofuro[3,2-b]furan-3,6-diol, 3.

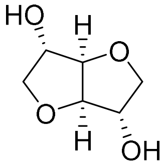

Isohexide dibenzoate (501 mg, 1.4 mmol) was dissolved in MeOH (20 mL) and the mixture was warmed at 55-60°C for 1h. After total dissolution 100µL (0.5 mmol) of 5M MeONa solution in MeOH were added. The mixture was stirred for 24h at room temperature and the reaction was monitored by TLC analysis (CH_2Cl_2:MeOH 9:1). The solution was neutralized adding about 1 g of DOWEX® G26 hydrogen form, then the resin was filtered off, the solution was diluted with 20 mL of CH_2Cl_2 and was extracted with distilled water (3x5 mL). The aqueous phases were combined and the solvent was removed under reduced pressure to give a slight yellow oil. The oil was dried over P_2O_5 overnight to give 204 mg (1.4 mmol, quantitative yield) of a white solid. Mp = 43-45 °C. $[\alpha]_D^{25}$ = +18.5 (c = 2.01; H_2O). ^1H NMR (400 MHz, D_2O, TMS, 21

°C, ppm) δ: 4.50 (s, 2H), 4.22 (d, *J* = 3.3 Hz, 2H), 3.76 (d, *J* = 10.4 Hz, 2H), 3.69 (dd, *J* = 10.4 Hz, *J* = 3.3 Hz, 2H). $^{13}C\{^1H\}$ NMR (126 MHz, D_2O, TMS, 21 °C, ppm) δ: 86.5, 74.8, 73.8.

General procedure for the synthesis of compounds 4.[2]

The isohexide (5.0 mmol) was dissolved in dry CH_2Cl_2 (25 mL) then dry pyridine (820 µL, 10.2 mmol) and trifluoromethanesulfonic anhydride (1.7 mL, 10.1 mmol) were slowly added at 0 °C. The mixture was stirred for 6h at room temperature and the reaction was monitored by TLC analysis (Hexane:Ethyl acetate 1:1). When total conversion was observed the reaction was quenched with 15 mL of cold distilled water. The phases were separated and the aqueous phase was washed with CH_2Cl_2 (3x 10 mL); the combined organic phases were extracted with 10% aqueous HCl (3x 5 mL), water (3x 5 mL) and dried over anhydrous Na_2SO_4. The solvent was removed under reduced pressure to give the product.

(3*R*,3a*S*,6*R*,6a*S*)-hexahydrofuro[3,2-b]furan-3,6-diylbis(trifluoromethanesulfonate), 4a

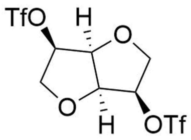

1.85 g (4.5 mmol, 90%) of a light pink foamy solid. mp = 61-62°C. $[\alpha]_D^{20}$= = +102.1 (c = 0.84; $CHCl_3$). ^1H NMR (401 MHz, $CDCl_3$, TMS, 21 °C, ppm) δ: 5.28 – 5.20 (m, 2H), 4.83 – 4.75 (m, 2H), 4.18 – 4.11 (m, 4H). $^{13}C\{^1H\}$ NMR (126 MHz, $CDCl_3$, TMS, 21 °C, ppm) δ: 118.6 (q, *J* = 319.4 Hz, CF_3), 83.5, 80.4, 70.9. ^{19}F NMR (378 MHz, $CDCl_3$, $CFCl_3$, 21 °C, ppm) δ: -74.9.

(3*R*,3a*S*,6*S*,6a*S*)-hexahydrofuro[3,2-b]furan-3,6-diylbis(trifluoromethanesulfonate), 4b

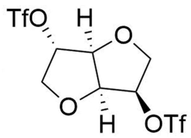

1.64 g (4.0 mmol, 80%) of the pure product as a light pink foamy solid. Mp = 113-114 °C. $[\alpha]_D^{20}$= +57.0 (c = 0.51; $CHCl_3$). ^1H NMR (500 MHz, $CDCl_3$, TMS, 21 °C, ppm) δ: 5.36 (d, *J* = 3.1 Hz, 1H), 5.26 (m, 1H), 5.02 (t, *J* = 5.4 Hz, 1H), 4.69 (d, *J* = 5.1 Hz, 1H), 4.31 (d, *J* = 11.6 Hz, 1H), 4.16 – 4.08 (d, *J* = 11.6 Hz, 2H), 3.92 (dd, *J* = 11.6 Hz, *J* = 4.6 Hz, 1H). $^{13}C\{^1H\}$ NMR (126 MHz, $CDCl_3$, TMS, 21 °C, ppm) δ 118.6 (q, *J* = 319.6 Hz, *C*F_3), 118.6 (q, *J* = 319.6 Hz, *C*F_3), 88.3, 86.0, 85.0, 81.1, 73.3, 71.5. ^{19}F NMR (471 MHz, $CDCl_3$, $CFCl_3$, 21 °C, ppm) δ: -74.6, -75.0

(3S,3aS,6S,6aS)-hexahydrofuro[3,2-b]furan-3,6-diylbis(trifluoromethanesulfonate), 4c

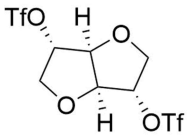

1.83 g (4.3 mmol, 89%) of a brown solid. mp = 64 °C, $[\alpha]_D^{25}$= +45.5 (c = 0.71, $CHCl_3$). ^1H NMR (400 MHz, $CDCl_3$, TMS, 21 °C, ppm) δ: 5.32 (d, *J* = 3.4 Hz, 2H), 4.91 (s, 2H), 4.22 (d, *J* = 12.0 Hz, 2H), 4.00 (dd, *J* = 12.0 Hz, *J* = 3.4 Hz, 2H). $^{13}C\{^1H\}$ NMR (126 MHz, $CDCl_3$, TMS, 21 °C, ppm) δ: 118.5 (q, *J* = 319.9 Hz, CF_3), 87.9, 85.1, 72.5. ^{19}F NMR (471 MHz, $CDCl_3$, $CFCl_3$, 21 °C, ppm) δ: -74.5.

General procedure for the synthesis of compounds 5[3-5]

Ditriflate **4** (5.0 mmol) was dissolved in DMF (5 mL), then NaN_3 (1.3 g, 20.0 mmol) was added to the solution. The mixture was stirred overnight at room temperature and the reaction was monitored by TLC analysis (Hexane:Acetone 8:2). When the reaction was complete 15 mL of Et_2O were added and the salts were removed by filtration. The filtrate was diluted with water (10 mL) and the aqueous phase was extracted with Et_2O (2x 10 mL). The organic phases were combined and dried over anhydrous Na_2SO_4. The solvent was removed under reduced pressure to obtain the product.

(3*S*,3a*R*,6*S*,6a*R*)-3,6-diazidohexahydrofuro[3,2-b]furan, 5a

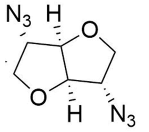

981 mg (5.0 mmol, quantitative yield) of a colorless oil. $[\alpha]_D^{20}$= +90.3 (c = 2.00; $CHCl_3$). ^1H NMR (500 MHz, $CDCl_3$, TMS, 21 °C, ppm) δ: 4.61 (s, 2H), 4.06 (dd, *J* = 4.0 Hz, *J* = 1.6 Hz, 2H), 3.93 (dd, *J* = 10.2 Hz, *J* = 1.6 Hz, 2H), 3.88 (dd, *J* = 10.2 Hz, *J* = 4.0 Hz, 2H). $^{13}C\{^1H\}$ NMR (126 MHz, $CDCl_3$, TMS, 21 °C, ppm) δ: 86.1, 71.9, 65.8.

(3*R*,3a*R*,6*S*,6a*R*)-3,6-diazidohexahydrofuro[3,2-b]furan, 5b

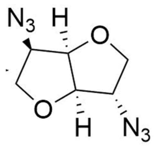

The crude was purified by flash chromatography (silica gel, Hexane:Acetone 8:2) to give 383 mg (1,9 mmol, 39%) of a colorless oil. $[\alpha]_D^{20}$= +156.5 (c = 0.55; $CHCl_3$). ^1H NMR (500 MHz, $CDCl_3$, TMS, 21 °C, ppm) δ: 4.82 – 4.78 (m, 1H), 4.50 (dt, *J* = 4.3 Hz, *J* = 0.9 Hz, 1H), 4.09 – 4.00 (m, 3H), 3.96 – 3.88 (m, 2H), 3.74 – 3.64 (m, 1H). $^{13}C\{^1H\}$ NMR (126 MHz, $CDCl_3$, TMS, 21 °C, ppm) δ: 87.1, 83.0, 72.9, 70.3, 66.2, 62.3.

(3*R*,3a*R*,6*R*,6a*R*)-3,6-diazidohexahydrofuro[3,2-b]furan, 5c

The crude product was purified by flash chromatography (silica gel, Hexane:Acetone 8:2)

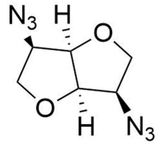

to give 500 mg (2.5 mmol, 51%) of a yellow oil. $[\alpha]_D^{20}$= +321.8 (c = 1.52; $CHCl_3$). ^1H NMR (400 MHz, $CDCl_3$, TMS, 21 °C, ppm) δ: 4.72 – 4.66 (m, 2H), 4.08 (dd, *J* = 8.7 Hz, *J* = 6.8 Hz, 2H), 3.94 – 3.86 (m, 2H), 3.80 (dd, *J* = 8.7 Hz, *J* = 8.5 Hz, 2H). $^{13}C\{^1H\}$ NMR (126 MHz, $CDCl_3$, TMS, 21 °C, ppm) δ: 83.4, 70.7, 62.4.

General procedure for the synthesis of compounds 6[3,6]

Diazide **5** (2.0 mmol) was dissolved in degassed EtOH (2 mL). The solution was placed in an autoclave and 10% Palladium on charcoal (106.0 mg, 0.1 mmol) was added. The autoclave was loaded with H_2 (3 bar) and the mixture was stirred for 24h at room temperature. The autoclave was discharged, the catalyst was filtered off and the solvent was removed under reduced pressure to give the pure product.

(3*S*,3a*R*,6*S*,6a*R*)-hexahydrofuro[3,2-b]furan-3,6-diamine, 6a

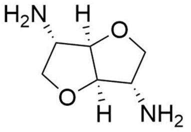

216 mg (1.5 mmol, 75%) of a white hygroscopic solid. mp = 75-77 °C. $[\alpha]_D^{20}$ = +24.2 (c = 0.12; DMSO). ^1H NMR (500 MHz, D_2O, TMS, 21 °C, ppm) δ: 4.38 (s, 2H), 3.75 (dd, *J* = 9.6 Hz, *J* = 4.5 Hz, 2H), 3.54 (dd, *J* = 9.6 Hz, *J* = 2.0 Hz, 2H), 3.34 (dd, *J* = 4.5 Hz, *J* = 2.0 Hz, 2H). $^{13}C\{^1H\}$ NMR (126 MHz, D_2O, TMS, 21 °C, ppm) δ: 87.9, 74.1, 56.8.

(3*R*,3a*R*,6*S*,6a*R*)-hexahydrofuro[3,2-b]furan-3,6-diamine, 6b

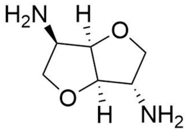

222 mg (1.5 mmol, 77%) of a colorless oil. $[\alpha]_D^{25}$ = +72.0 (c = 0.3; MeOH). ^1H NMR (400 MHz, [D4]Methanol, TMS, 21 °C, ppm) δ: 5.74 (t, *J* = 4.3 Hz, 1H), 5.62 (d, *J* = 4.0 Hz, 1H), 5.16 (m, 2H), 4.96 (dd, *J* = 9.3 Hz, *J* = 2.1 Hz, 1H), 4.69 – 4.62 (m, 2H), 4.52 (dd, *J* = 9.9 Hz, *J* = 8.2 Hz, 1H) . $^{13}C\{^1H\}$ NMR (126 MHz, [D4]Methanol, TMS, 21 °C, ppm) δ: 89.5, 82.2 , 75.1, 72.1, 58.9, 55.3.

(3R,3aR,6R,6aR)-hexahydrofuro[3,2-b]furan-3,6-diamine, 6c

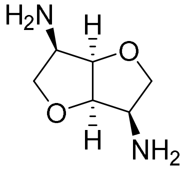

288 mg (2.0 mmol, quantitative yield) of a white gelatinous solid. $[\alpha]_D^{25}$ = +79.1 (c = 0.75; MeOH). ^1H NMR (400 MHz, [D4]Methanol, TMS, 21 °C, ppm) δ: 4.39 – 4.30 (m, 2H), 3.98 (td, *J* = 8.0 Hz, *J* = 2.8 Hz, 2H), 3.42 (m, 2H), 3.23 (td, *J* = 8.0 Hz, *J* = 2.8 Hz, 2H). $^{13}C\{^1H\}$ NMR (126 MHz, [D4]Methanol, TMS, 21 °C, ppm) δ: 83.4, 73.3, 56.1.

Synthesis of *N*-acetylamino acids 11a-c,f,g

Procedure A.[7] Acetic anhydride (2.7 equiv) was dropwise added to a solution of the amino acid (1 equiv, 1.2 M) in methanol (10 mL). The mixture was stirred under reflux for 6 hours and then was cooled to room temperature. The solvent was removed under reduced pressure to give the crude product that was kept overnight at – 18 °C.

(*S*)-*N*-acetylleucine, 11a. The crude product was triturated with $CHCl_3$, giving a white solid (1.16 g, 6.7 mmol, 56%). ^{1}H NMR (600 MHz, [D$_6$]DMSO, TMS, 25 °C, ppm) δ: 8.05 (d, *J* = 7.9 Hz, 1H), 5.16 (ddd, *J* = 9.5 Hz, *J* = 7.9 Hz, *J* = 5.5 Hz, 1H), 1.80 (s, 3H), 1.60 (m, 1H), 1.46 (m, 2H), 0.86 (d, *J* = 6.7 Hz, 3H), 0.81 (d, *J* = 6.7 Hz, 3H). ^{13}C{^{1}H} NMR (151 MHz, [D$_6$]DMSO, TMS, 25 °C, ppm) δ: 174.7, 169.7, 50.6, 24.8, 23.3, 22.76, 21.75.

(*R*)-*N*-acetylleucine, 11a. The crude product was triturated with $CHCl_3$, giving a white solid (1.72 g, 9.9 mmol, 82%).

(*R*)-*N*-acetylalanine, 11b. The crude product was triturated with $CHCl_3$ giving a white solid (1.43 g, 11.0 mmol, 90%). ^{1}H NMR (600 MHz, [D$_6$]DMSO, TMS, 25 °C, ppm) δ: 8.11 (d, *J* = 7.3 Hz, 1H), 4.14 (dq, *J* = 7.4 Hz, *J* = 7.3 Hz, 1H), 1.80 (s, 3H), 1.22 (d, *J* = 7.4 Hz, 3H).^{13}C{^{1}H} NMR (151 MHz, [D$_6$]DMSO, TMS, 25 °C, ppm) δ: 174.7, 169.5, 47.9, 22.8, 17.6.

(*S*)-*N*-acetylalanine, 11b. The crude product was triturated with $CHCl_3$, giving a white solid (0.92 g, 7.0 mmol, 58%).

(*S*)-*N*-acetylphenylalanine, 11f. Cold deionized water was added to the crude product to give a white crystal solid (1.07 g, 5.2 mmol, 43%). ^{1}H NMR (600 MHz, [D$_6$]DMSO, TMS, 25 °C, ppm) δ: 8.15 (d, J = 8.1 Hz, 1H), 7.25 (t, J = 7.2 Hz, 2H), 7.20 (d, J = 7.2 Hz, 2H), 7.18 (t, J = 7.2 Hz, 1H), 4.37 (ddd, *J* = 9.5 Hz, *J* = 8.1 Hz, *J* = 4.9 Hz, 1H), 3.01 (dd, *J* = 13.8 Hz, *J* = 4.9 Hz, 1H), 2.80 (dd, *J* = 13.8 Hz, *J* = 9.5 Hz, 1H), 1.75 (s, 3H).^{13}C{^{1}H} NMR (151 MHz, [D$_6$]DMSO, TMS, 25 °C, ppm) δ 172.9, 169.0,137.5, 128.8, 127.9, 126.2, 53.3, 36.5, 22.1.

(*S*)-*N*-acetylvaline, 11g. The crude product was triturated with CH_2Cl_2, giving a white solid (1.31 g, 8.3 mmol, 68%). ^{1}H NMR (600 MHz, [D$_6$]DMSO, TMS, 25 °C, ppm) δ: 7.95 (d, *J* = 8.5 Hz, 1H), 4.10 (dd, *J* = 8.5 Hz, *J* = 5.8 Hz, 1H), 2.00 (m, 1H), 1.85 (s, 3H), 0.85 (d, *J* = 7.0 Hz, 6H). ^{13}C{^{1}H} NMR (151 MHz, [D$_6$]DMSO, TMS, 25 °C, ppm) δ: 173.2, 169.6, 57.2, 29.8, 22.3, 19.2, 18.1.

(*R*)-*N*-acetylvaline, 11g. The product was obtained as a with solid (1.06 g, 6.68 mmol, 55%).

Procedure B.[8] Acetic anhydride (1.2 equiv.) was added to a suspension of the amino acid (12 mmol) in 5% $NaHCO_3$ (33.7 mL) at 0 °C. The mixture was stirred at room temperature for 4 hours. The reaction was monitored by TLC analysis (ethanol: acetic acid 1 M = 9:1) and was stopped when conversion appeared complete. The mixture was acidified to pH 2-3 with 6 M HCl and manteined at 0 °C overnight. The crude product was filtered and washed with cold water to give the chemically pure product.

(*R*)-*N*-acetylalanine, 11b. White solid (2.14 g, 10.3 mmol, 85%).

(*S*)-*N*-acetylphenylglycine (11c). White solid (1.25 g, 6.5 mmol, 54%).^{1}H NMR (600 MHz, [D$_6$]DMSO, TMS, 25 °C, ppm) δ: 8.60 (d, *J* = 7.5 Hz, 1H), 7.38-7.27 (m, 5H), 5.31 (d, *J* = 7.5 Hz, 1H), 1.89 (s, 3H). ^{13}C{^{1}H} NMR (151 MHz, [D$_6$]DMSO, TMS, 25 °C, ppm) δ: 172.4, 169.6, 137.8, 128.9, 128.3, 128.1, 56.7, 22.7.

(*R*)-*N*-acetylphenylglycine, 11c. Needle-like white solid (0.92 g, 4.8 mmol, 39%).

(*S*)-*N*-acetylphenylalanine, 11f. A white needle-liked solid (1.96 g, 9.45 mmol, 78%).

^{1}H and ^{13}C{^{1}H} NMR spectra

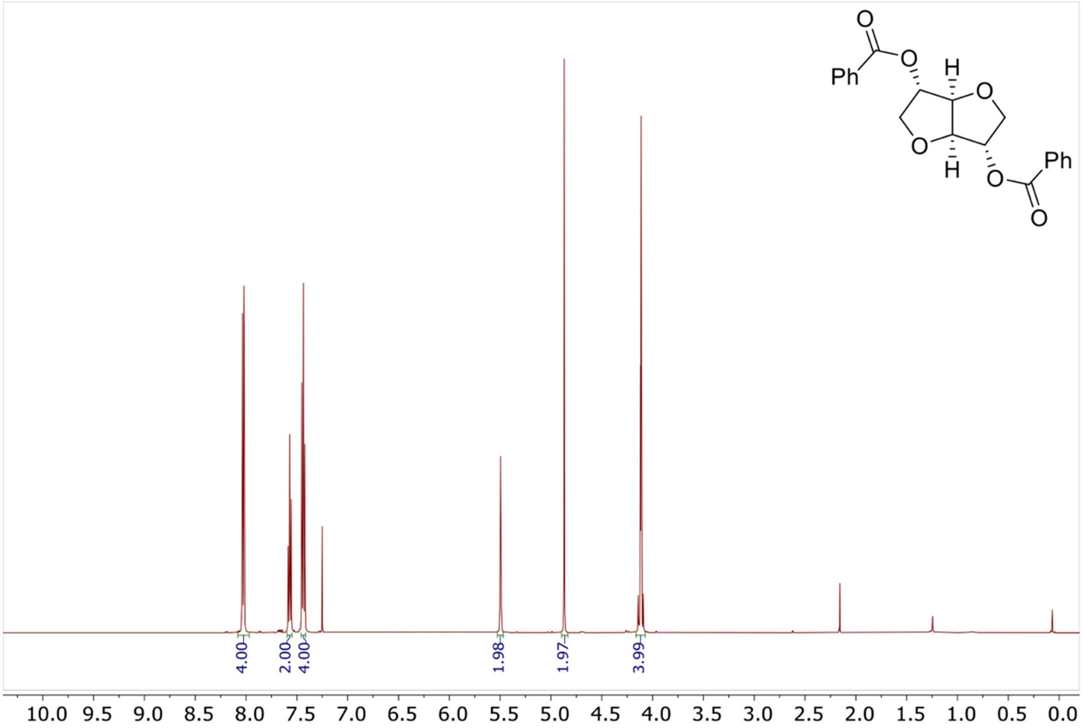

Figure SI1: ^{1}H NMR (500 MHz, [D$_4$]methanol, TMS, 21 °C, ppm) spectrum of diphenyl (3*S*,3a*R*,6*S*,6a*R*)-hexahydrofuro[3,2-b]furan-3,6-dicarboxylate.

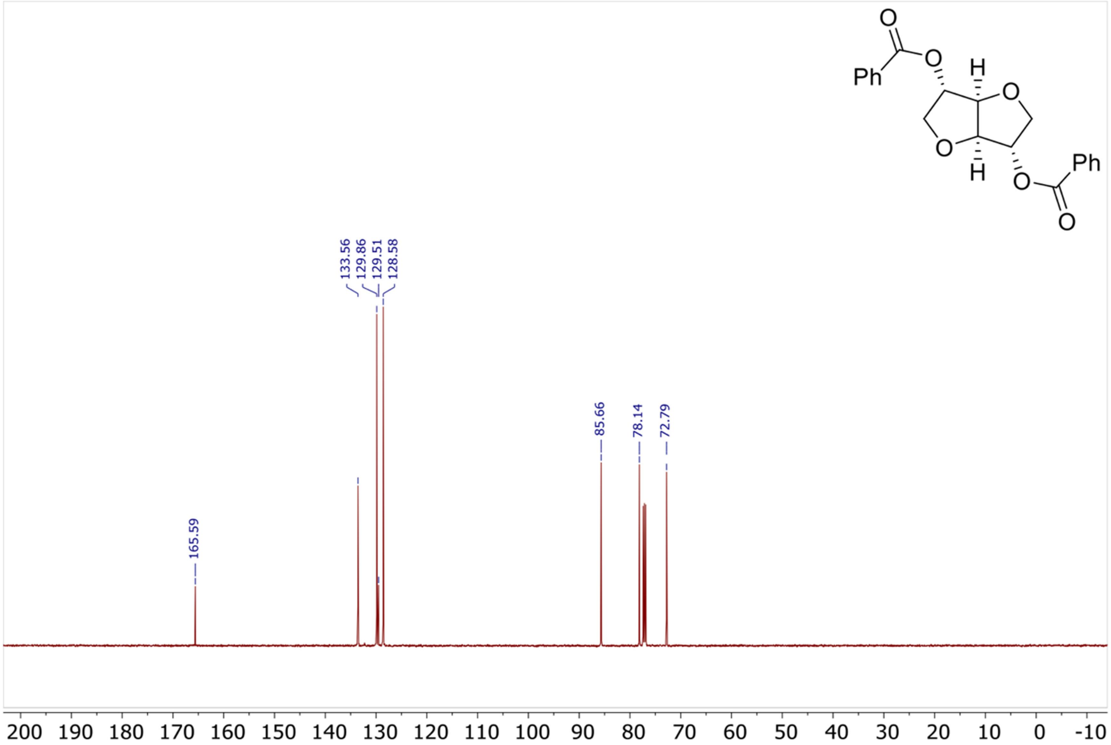

Figure SI2: $^{13}C\{^{1}H\}$ NMR (126 MHz, D_2O, TMS, 21 °C, ppm) spectrum of diphenyl (3*S*,3a*R*,6*S*,6a*R*)-hexahydrofuro[3,2-b]furan-3,6-dicarboxylate.

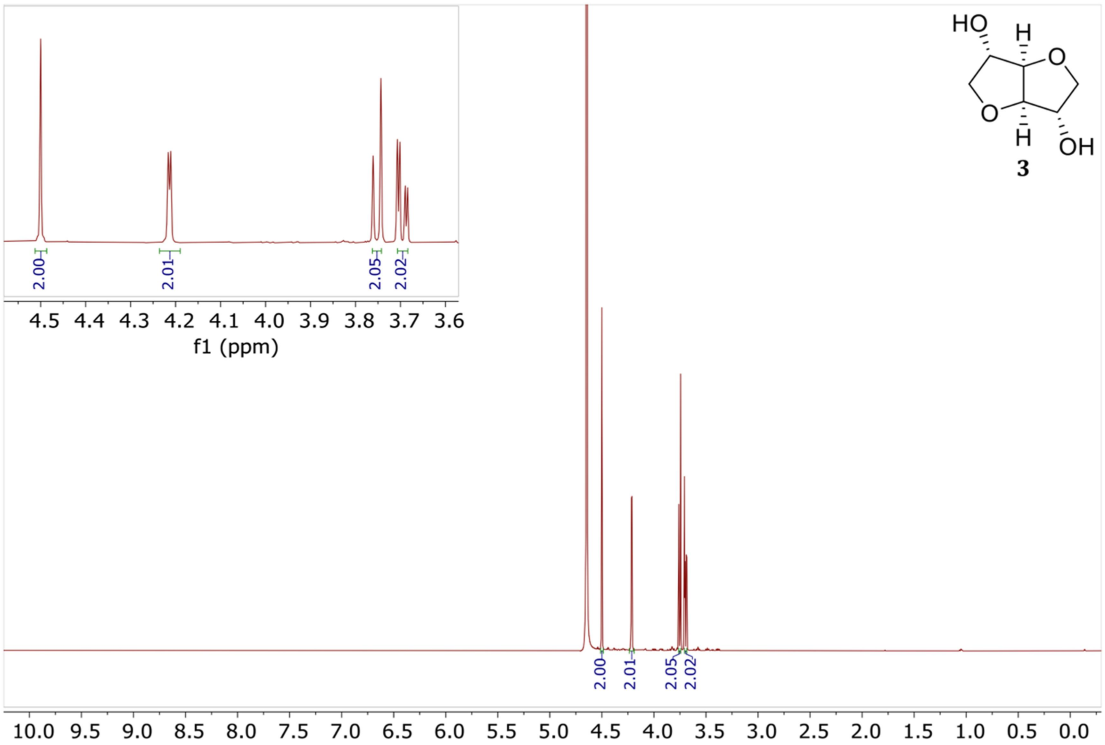

Figure SI3: ^{1}H NMR (400 MHz, D_20, TMS, 21 °C, ppm) spectrum of compound **3.**

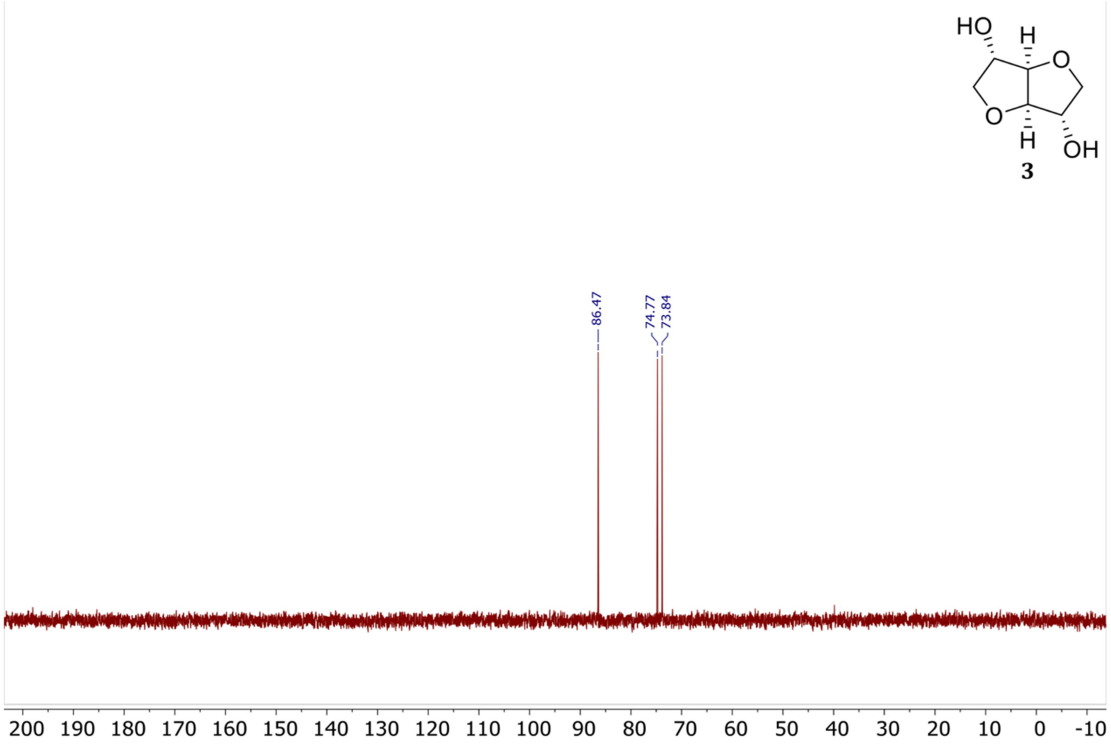

Figure SI4: $^{13}C\{^{1}H\}$ NMR (126 MHz, D_2O, TMS, 21 °C, ppm) spectrum of **3**.

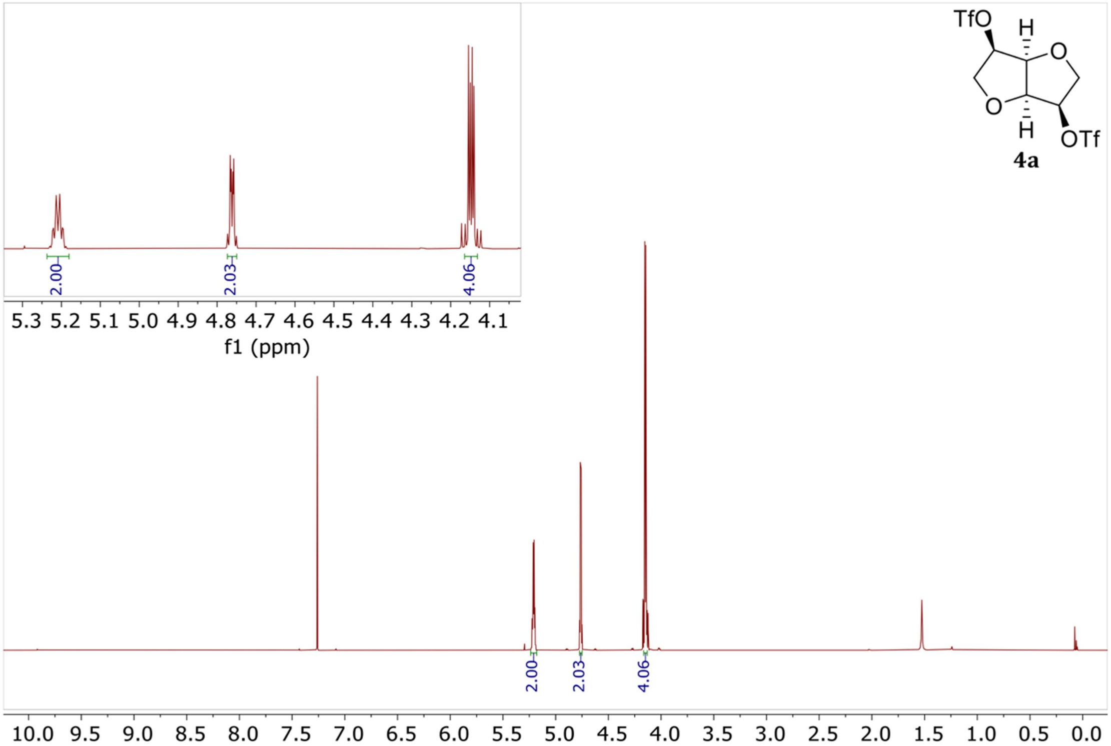

Figure SI5: ^{1}H NMR (500 MHz, $CDCl_3$, TMS, 21 °C, ppm) spectrum of **4a**.

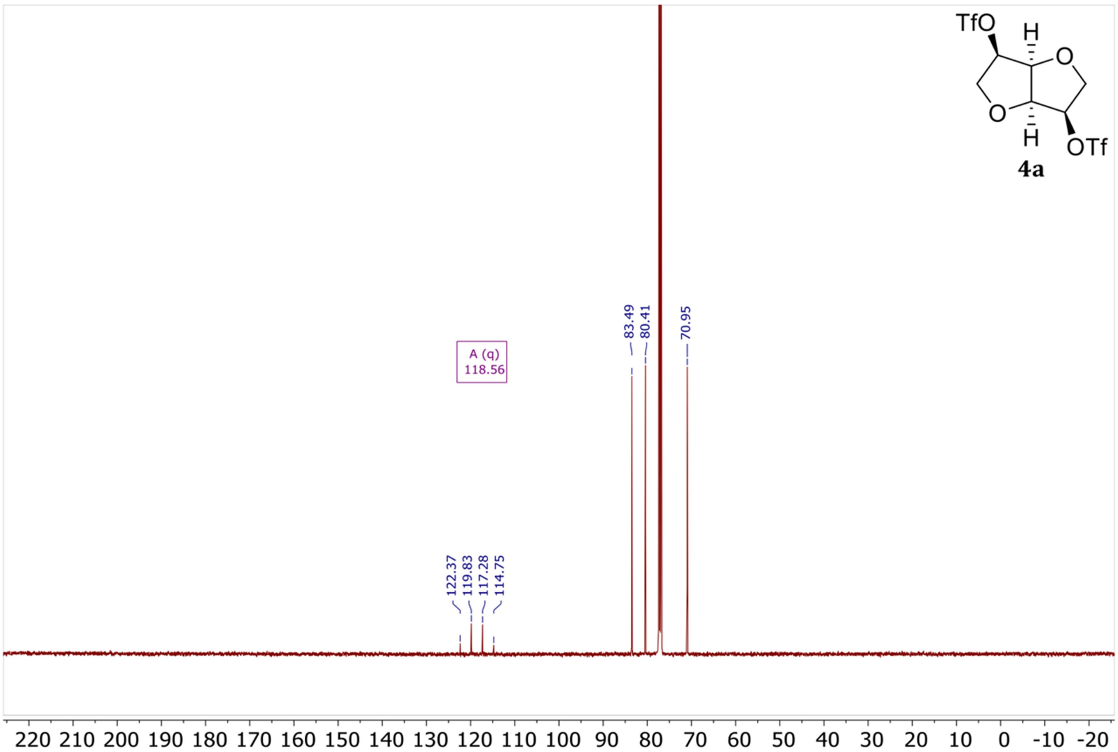

Figure SI6: $^{13}C\{^{1}H\}$ NMR (126 MHz, D_2O, TMS, 21 °C, ppm) spectrum of **4a**.

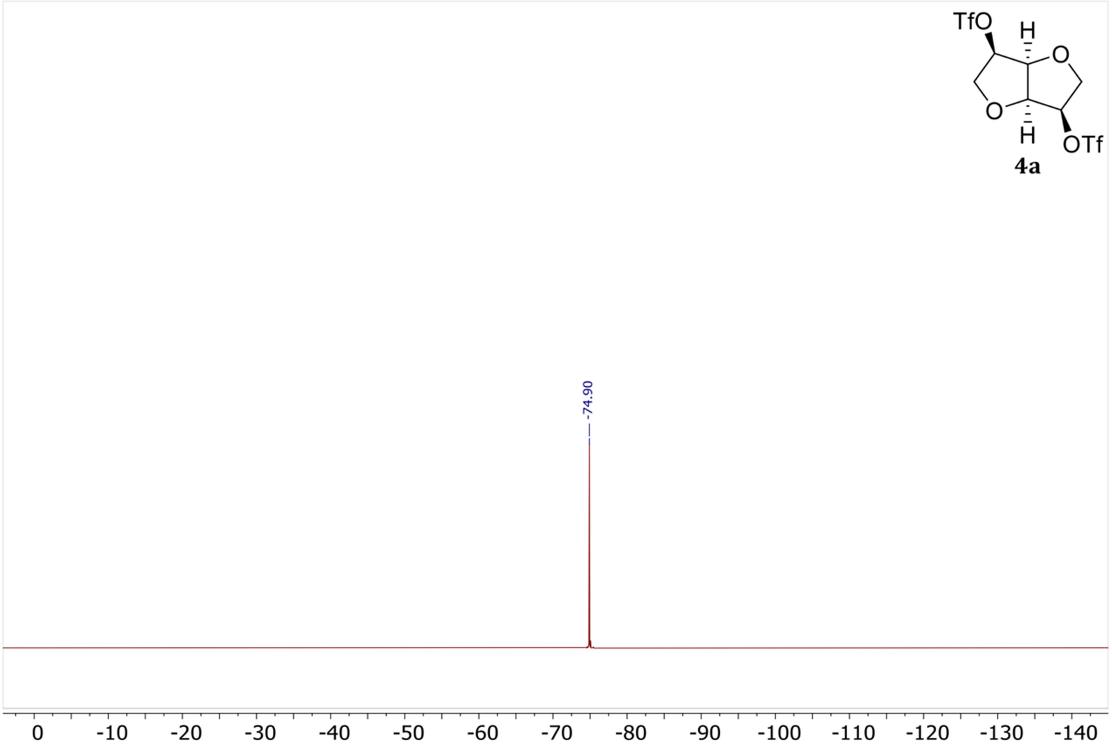

Figure SI7: ^{19}F NMR (378 MHz, $CDCl_3$, $CFCl_3$, 21 °C, ppm) spectrum of **4a**.

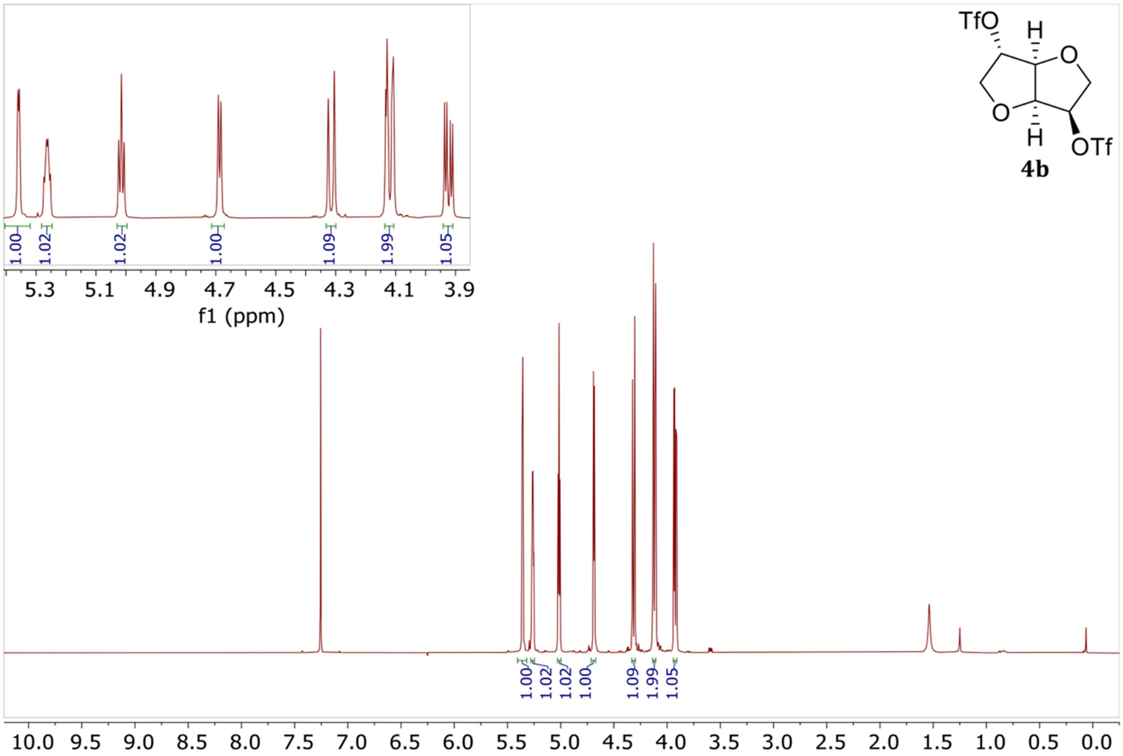

Figure SI8: ^{1}H NMR (500 MHz, $CDCl_3$, TMS, 21 °C, ppm) spectrum of **4b**.

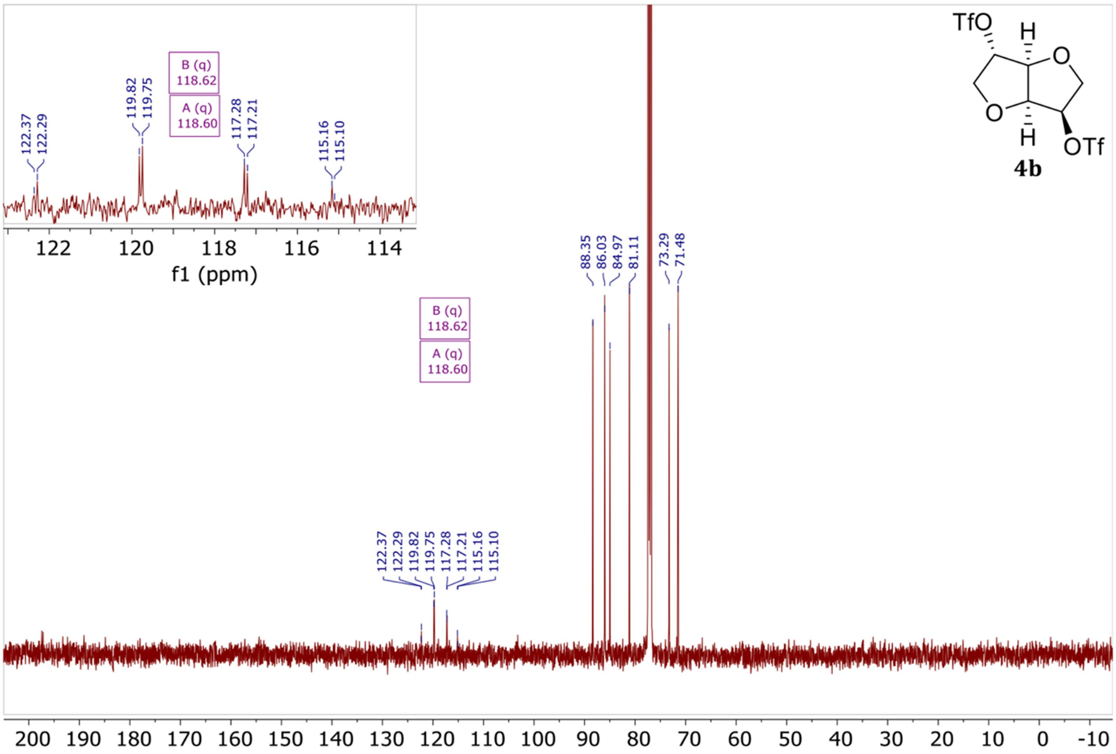

Figure SI9: $^{13}C\{^{1}H\}$ NMR (126 MHz, $CDCl_3$, TMS, 21 °C, ppm) spectrum of **4b**.

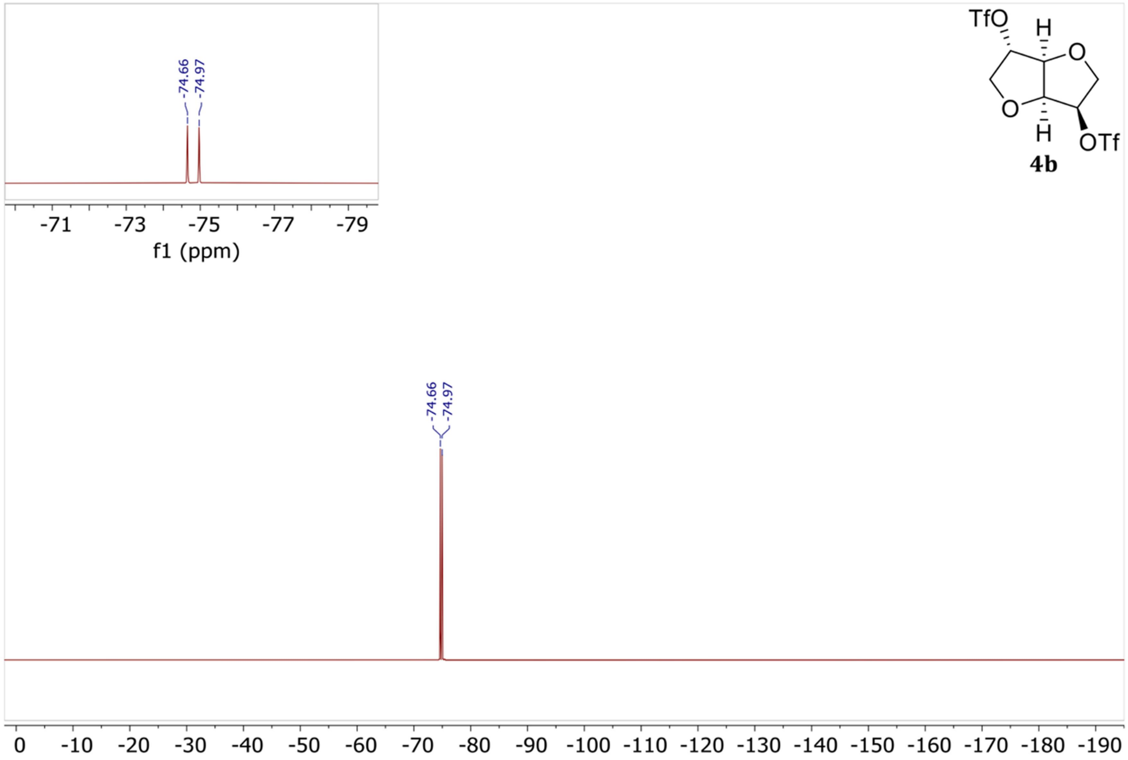

Figure SI10: ^{19}F NMR (378 MHz, $CDCl_3$, $CFCl_3$, 21 °C, ppm) spectrum of **4b**.

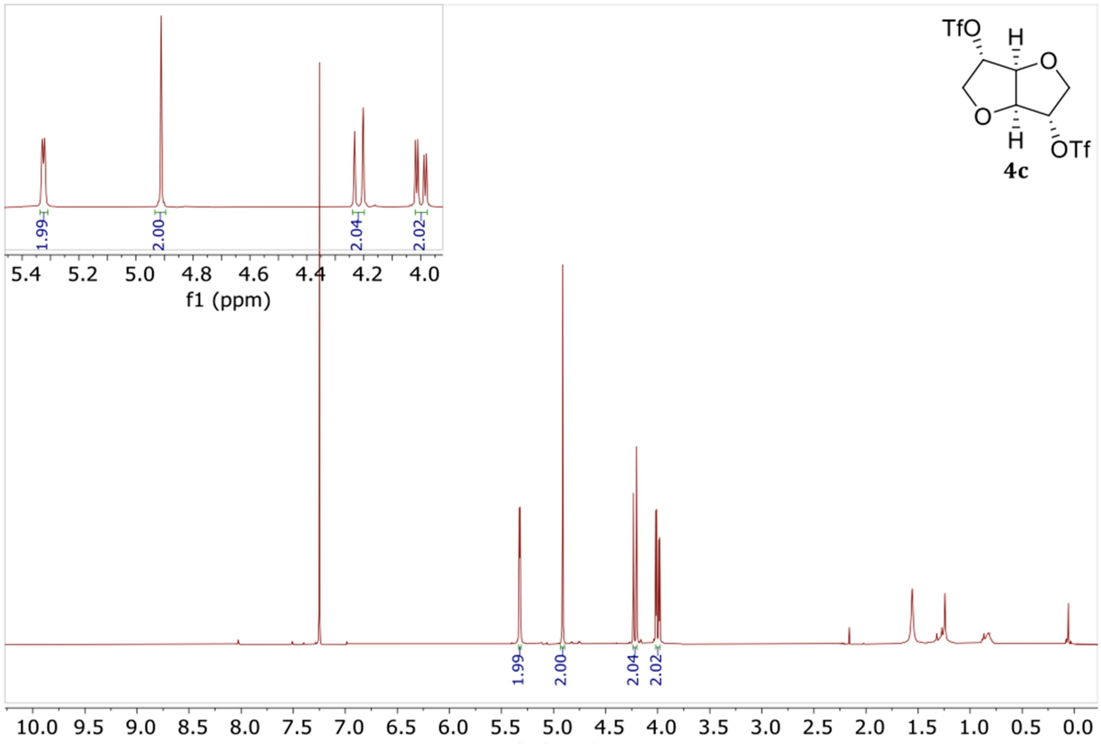

Figure SI11: ^{1}H NMR (500 MHz, $CDCl_3$, TMS, 21 °C, ppm) spectrum of **4c**.

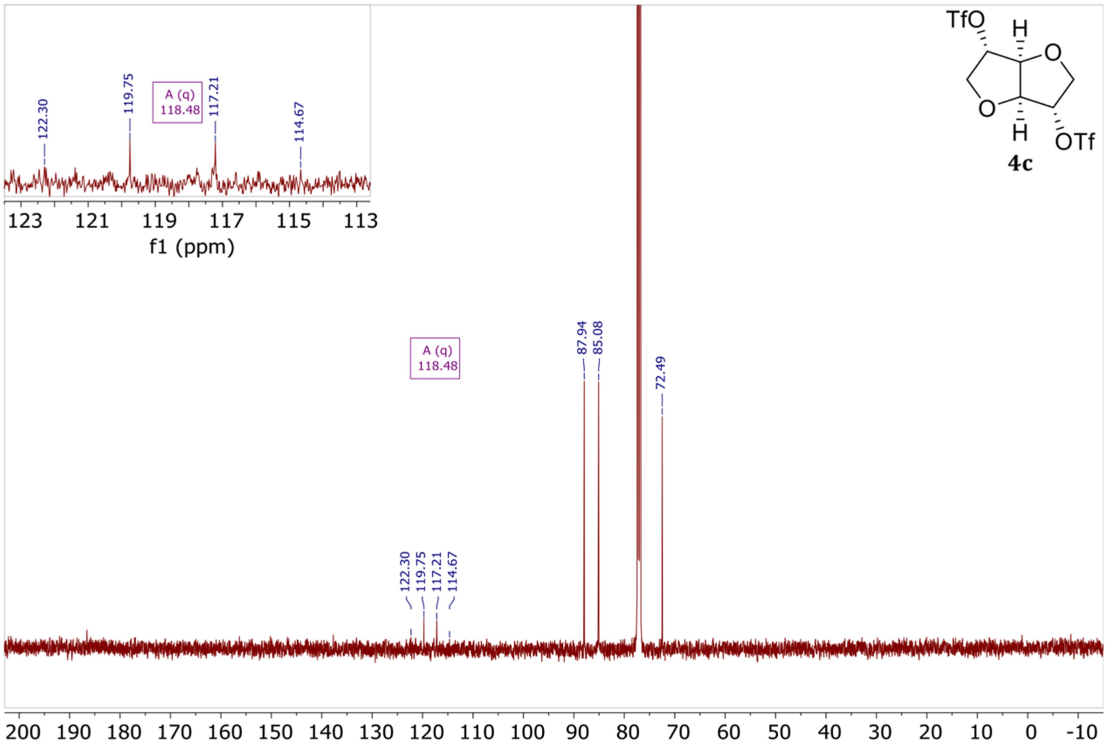

Figure SI12: $^{13}C\{^{1}H\}$ NMR (126 MHz, $CDCl_3$, TMS, 21 °C, ppm) spectrum of **4c.**

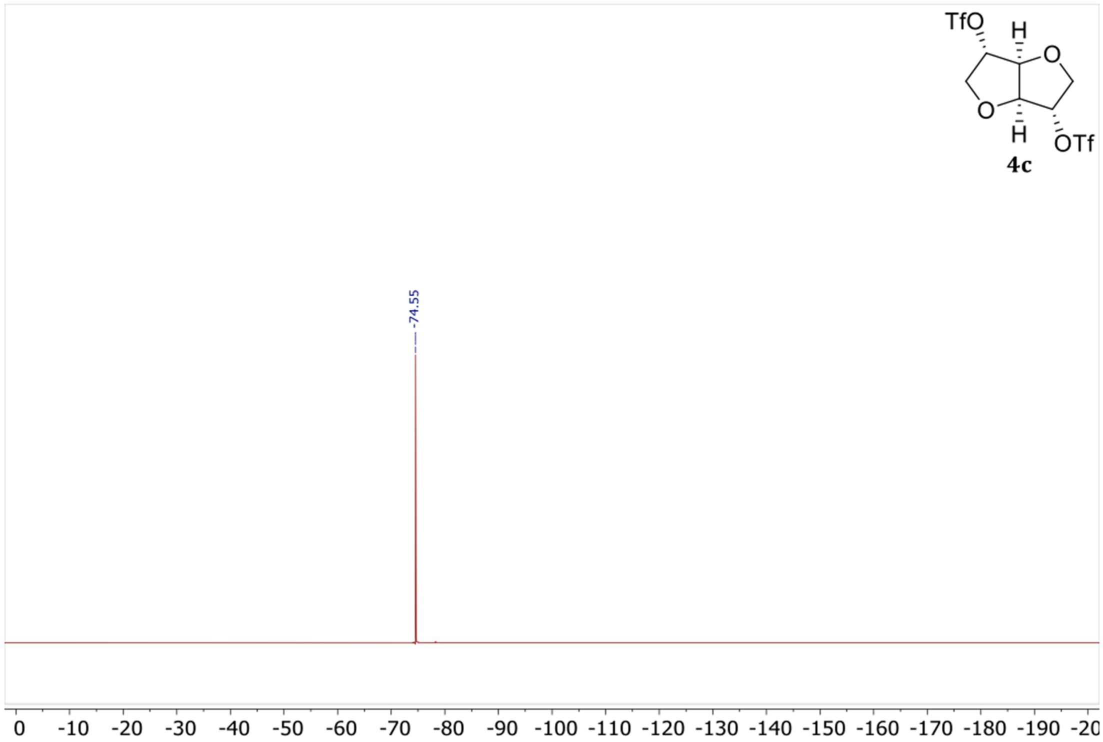

Figure SI13: ^{19}F NMR (378 MHz, $CDCl_3$, $CFCl_3$, 21 °C, ppm) spectrum of **4c**.

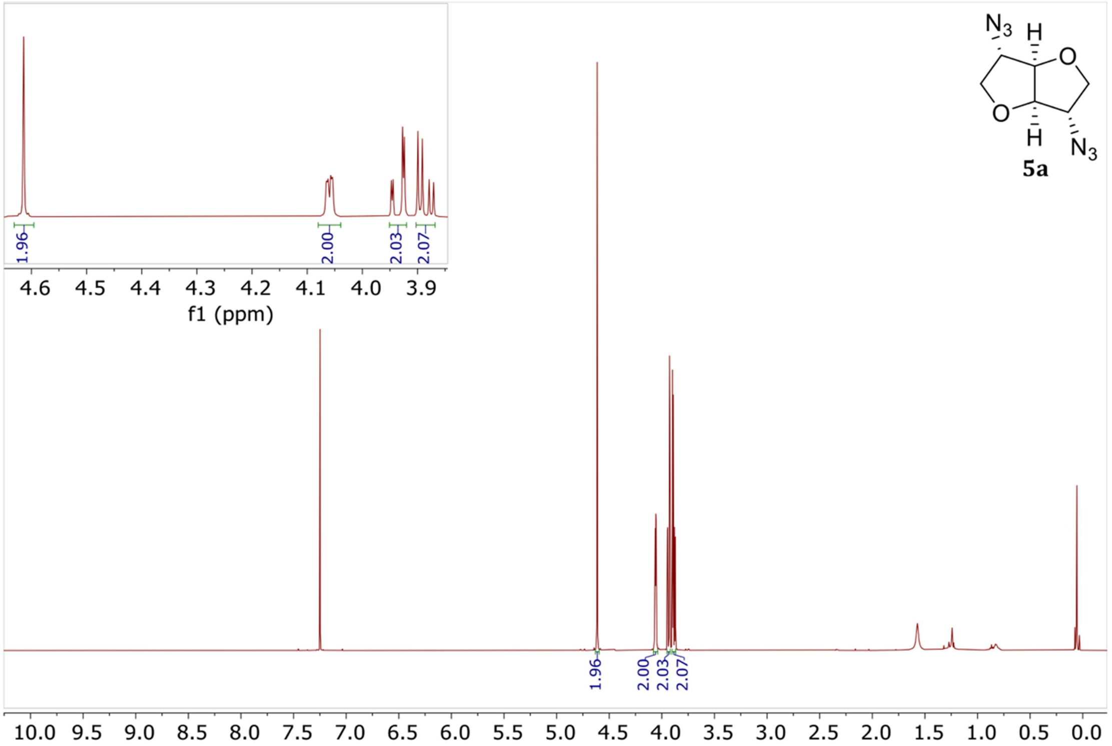

Figure SI14: ^{1}H NMR (500 MHz, $CDCl_3$, TMS, 21 °C, ppm) spectrum of **5a**.

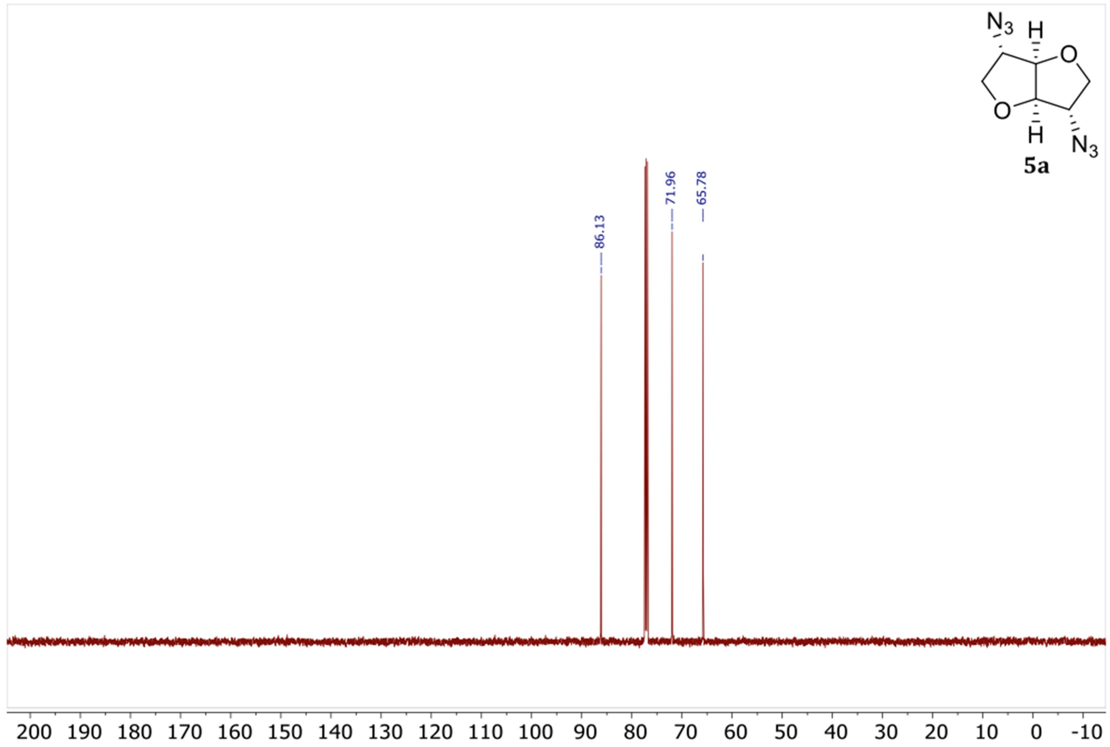

Figure SI15: $^{13}C\{^{1}H\}$ NMR (126 MHz, $CDCl_3$, TMS, 21 °C, ppm) spectrum of **5a**.

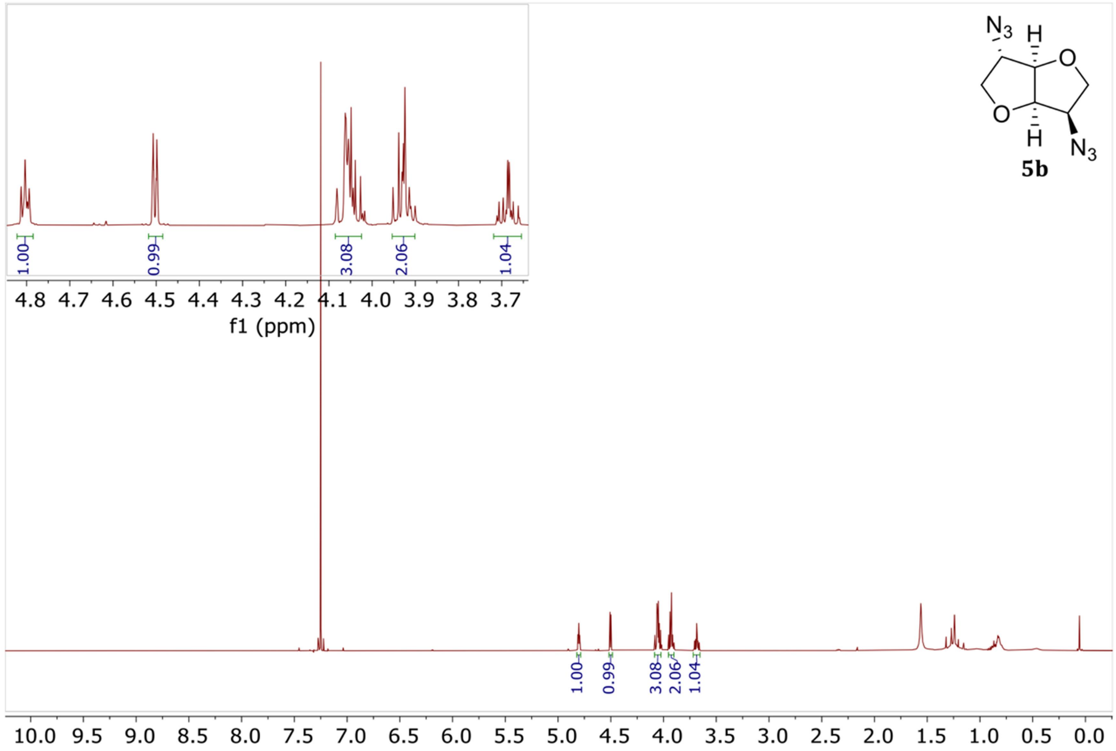

Figure SI16: ^{1}H NMR (500 MHz, $CDCl_3$, TMS, 21 °C, ppm) spectrum of **5b.**

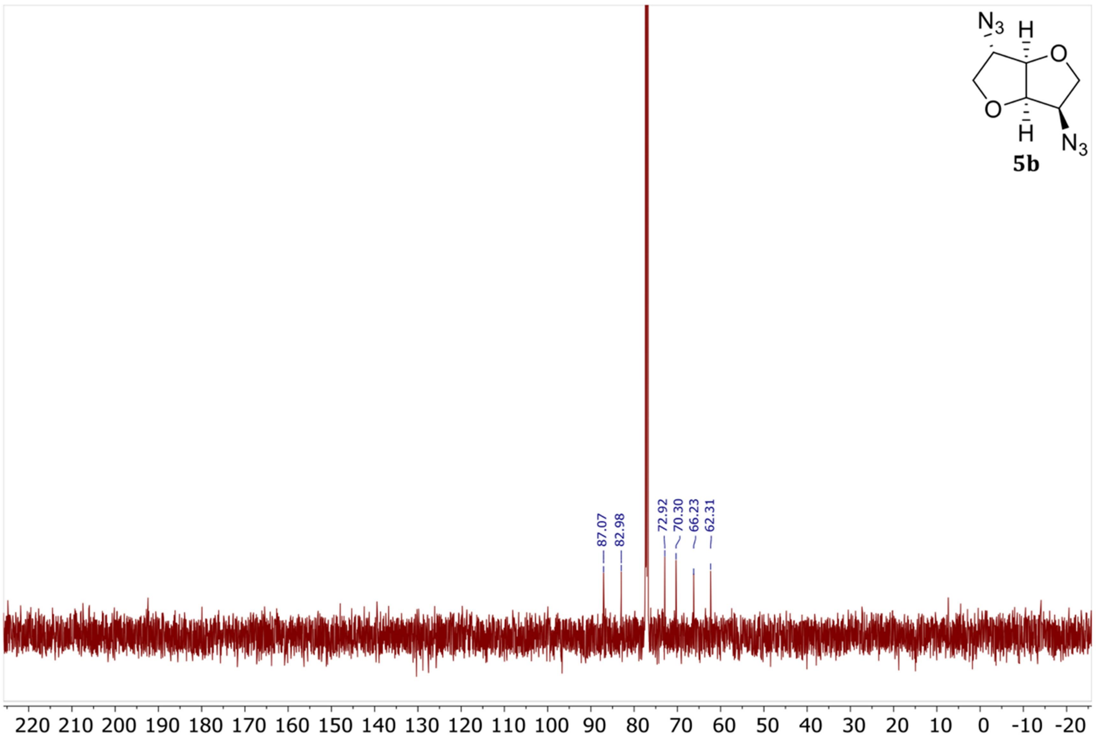

Figure SI17: $^{13}C\{^1H\}$ NMR (126 MHz, $CDCl_3$, TMS, 21 °C, ppm) spectrum of **5b**.

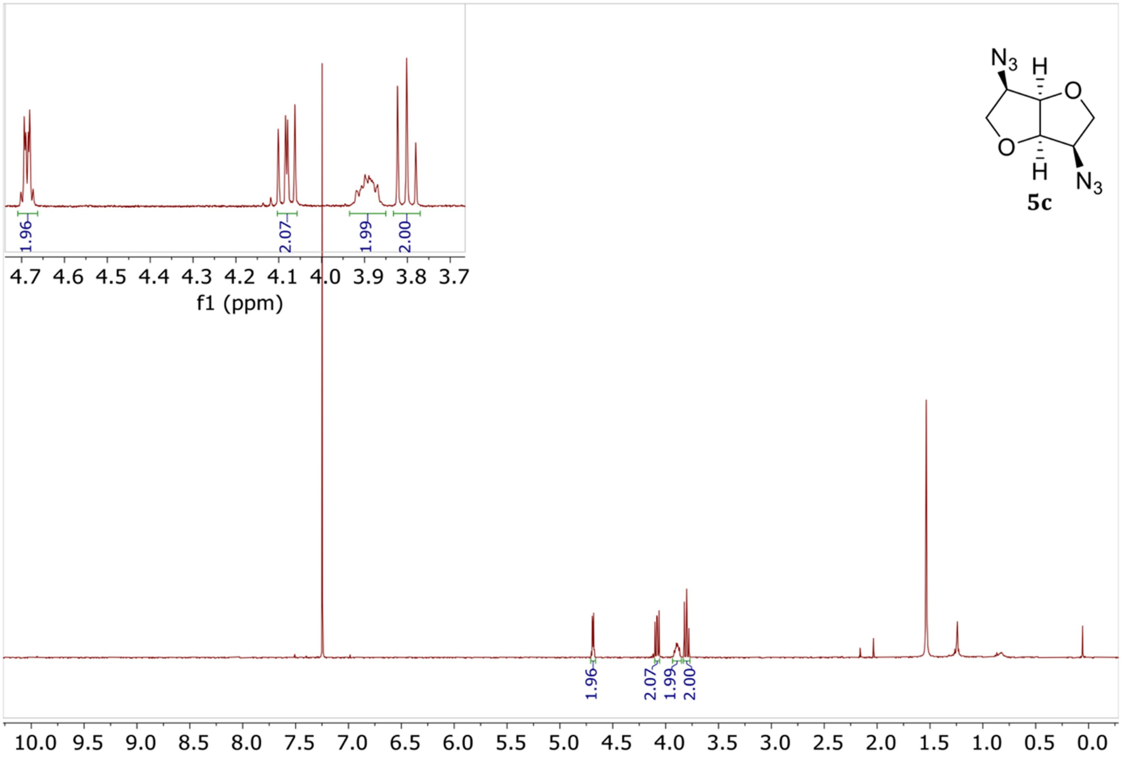

Figure SI18: ^1H NMR (400 MHz, $CDCl_3$, TMS, 21 °C, ppm) spectrum of **5c.**

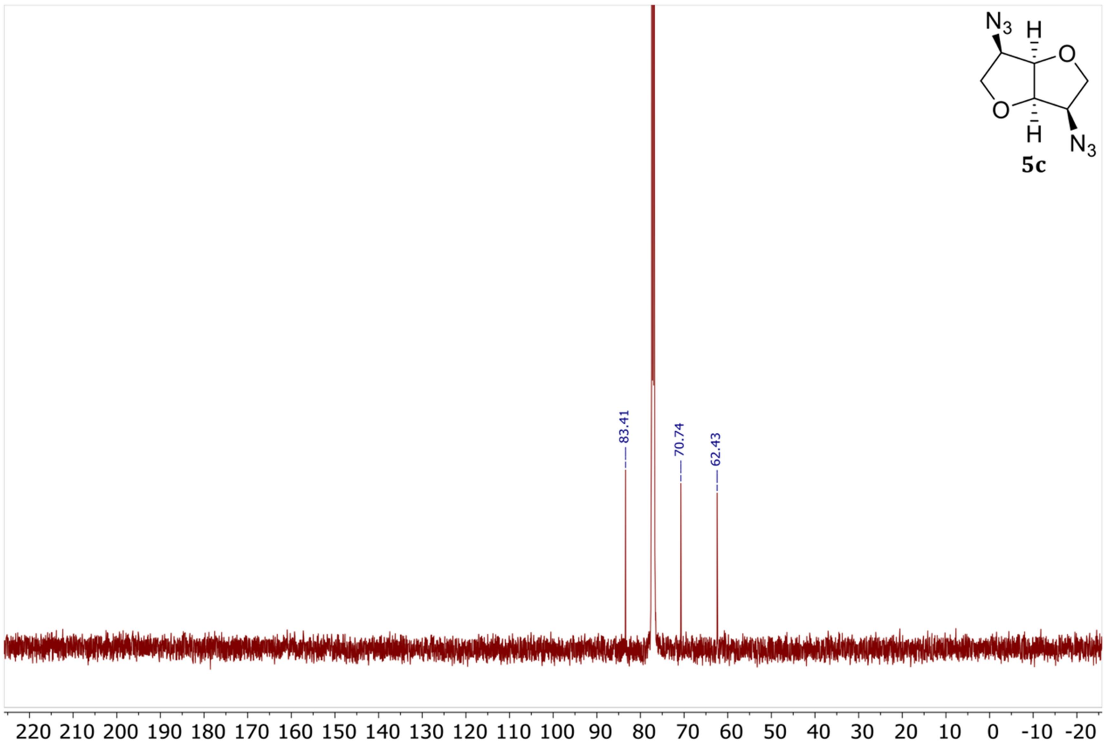

Figure SI19: $^{13}C\{^1H\}$ NMR (126 MHz, $CDCl_3$, TMS, 21 °C, ppm) spectrum of **5c**.

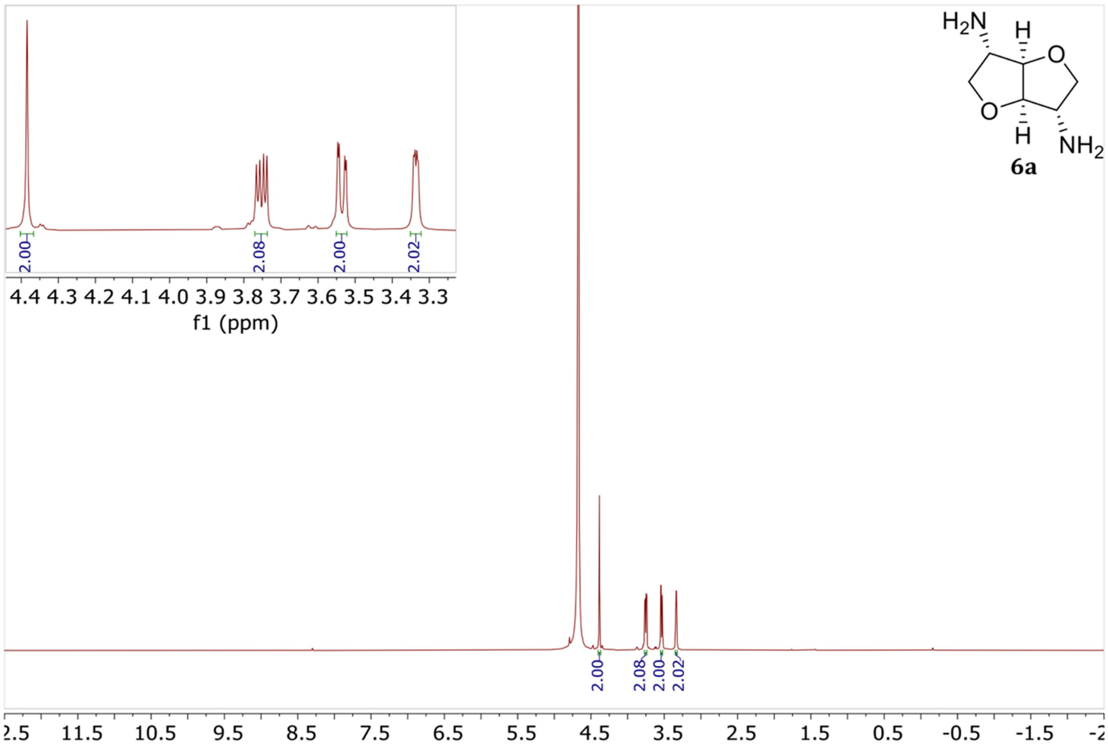

Figure SI20: ^1H NMR (400 MHz, D_2O, TMS, 21 °C, ppm) spectrum of **6a.**

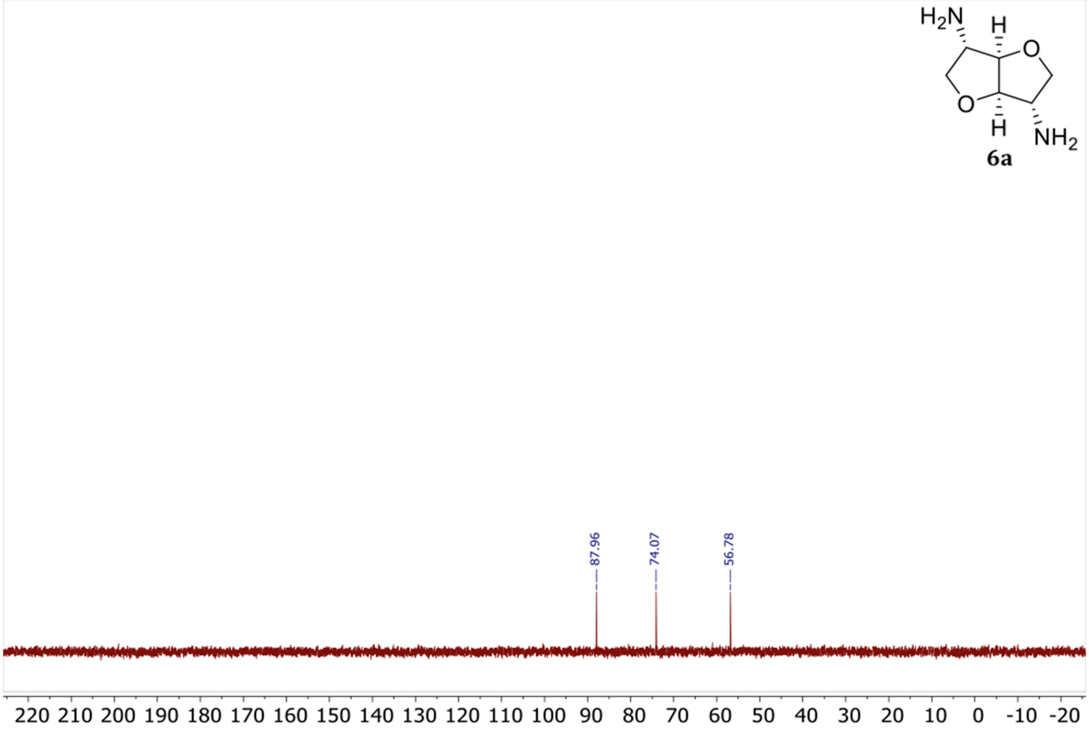

Figure SI21: $^{13}C\{^1H\}$ NMR (126 MHz, D_2O, TMS, 21 °C, ppm) spectrum of **6a**.

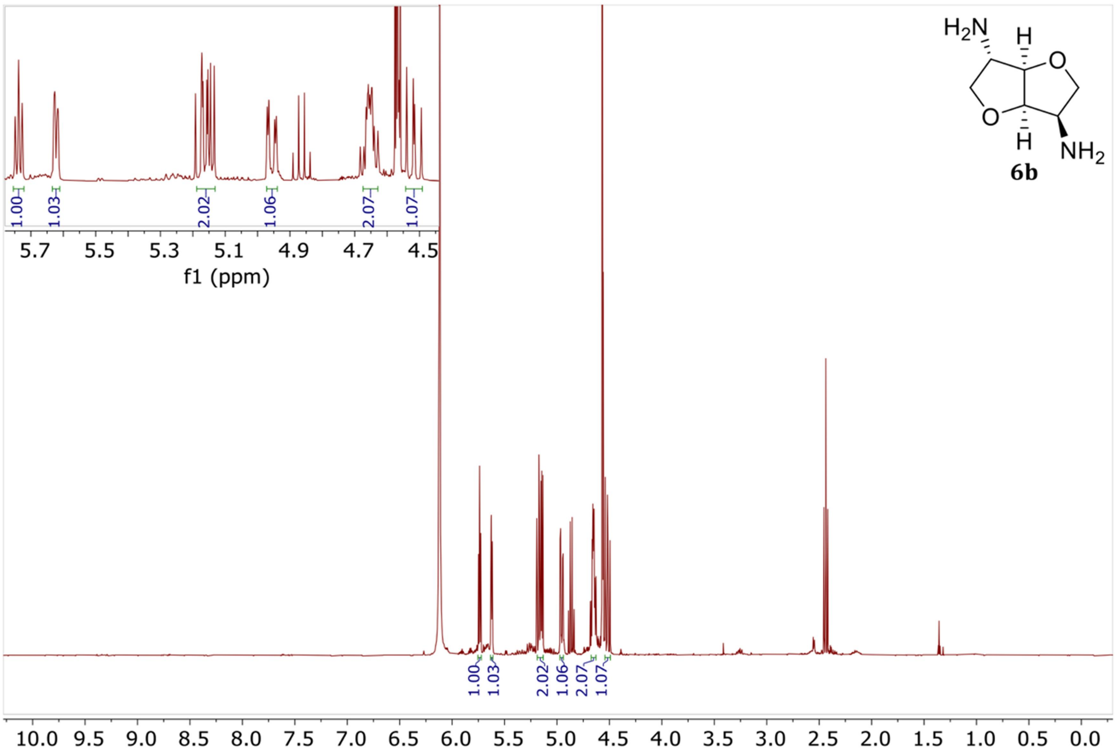

Figure SI22: ^1H NMR (400 MHz, [D_4]methanol, TMS, 21 °C, ppm) spectrum of **6b.**

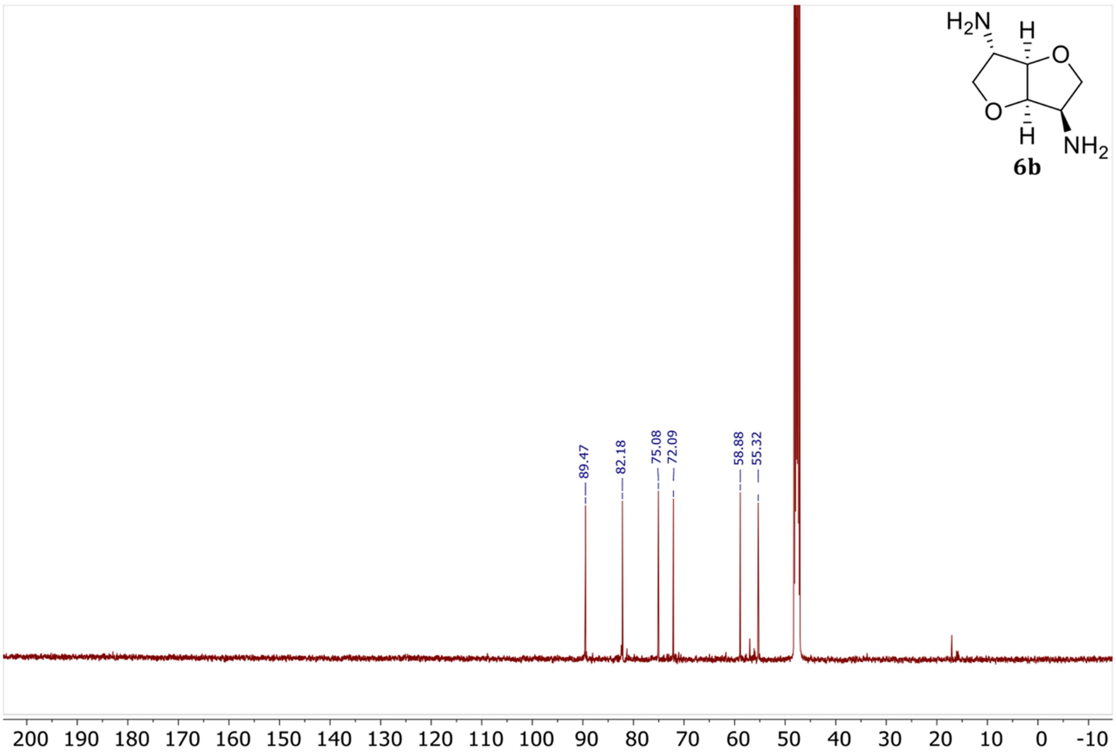

Figure SI23: $^{13}C\{^{1}H\}$ NMR (126 MHz, [D_4]methanol, TMS, 21 °C, ppm) spectrum of **6b**.

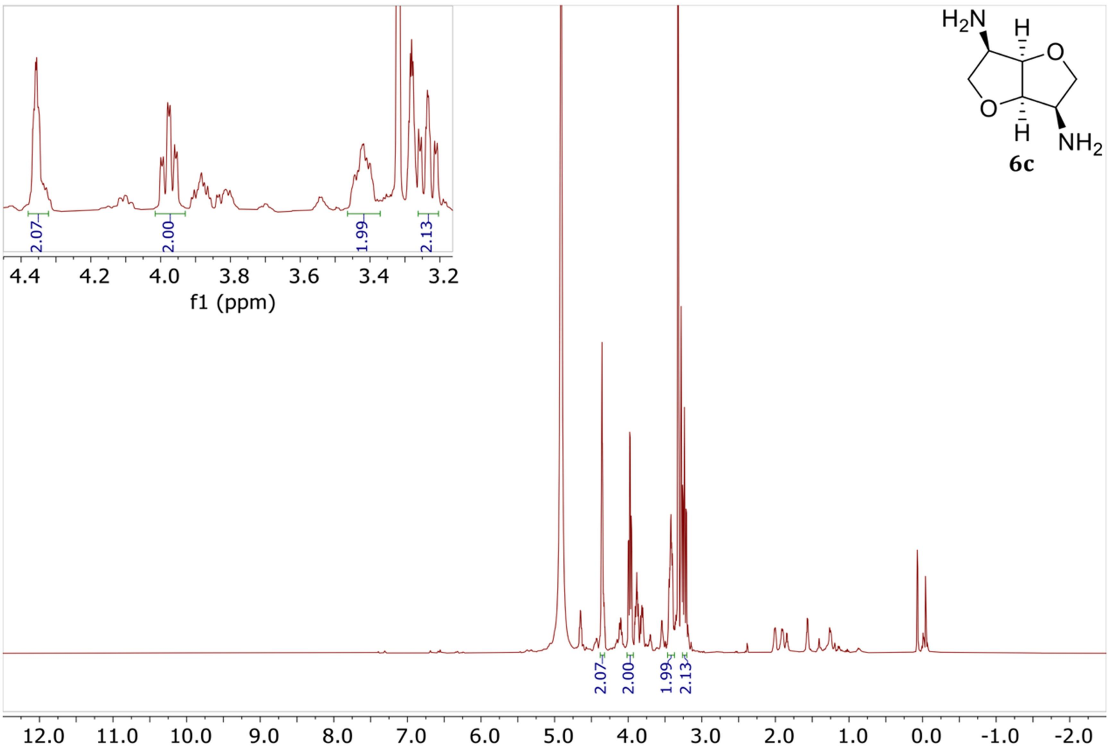

Figure SI24:^{1}H NMR (400 MHz, [D_4]methanol, TMS, 21 °C, ppm) spectrum of **6c.**

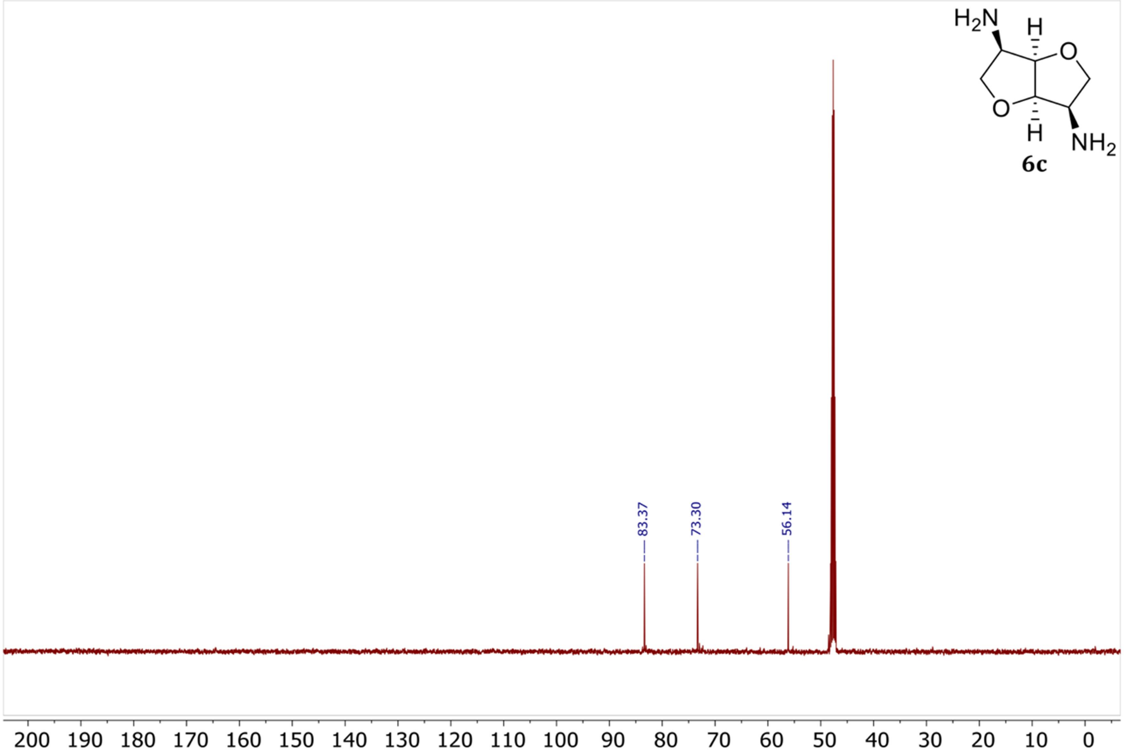

Figure SI25: $^{13}C\{^{1}H\}$ NMR (126 MHz, [D$_4$]methanol, TMS, 21 °C, ppm) spectrum of **6c**.

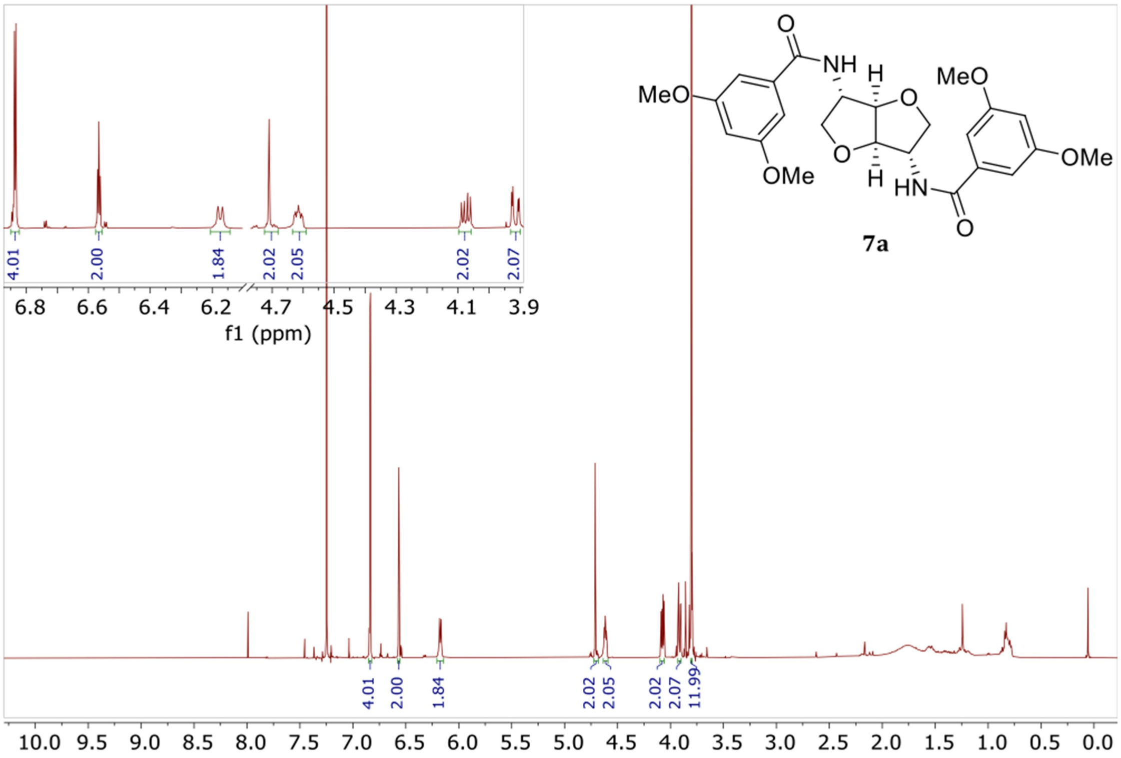

Figure SI26: ^{1}H NMR (500 MHz, $CDCl_3$, TMS, 21 °C, ppm) spectrum of **7a.**

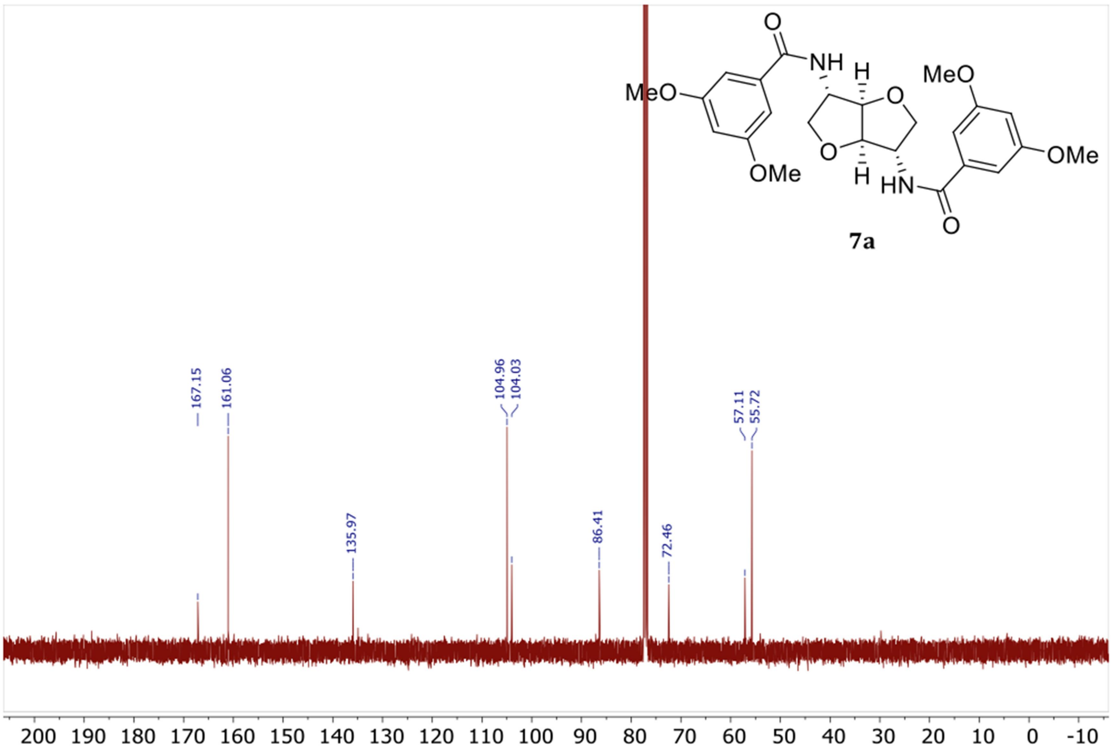

Figure SI27: $^{13}C\{^1H\}$ NMR (101 MHz, $CDCl_3$, TMS, 21 °C, ppm) spectrum of **7a**.

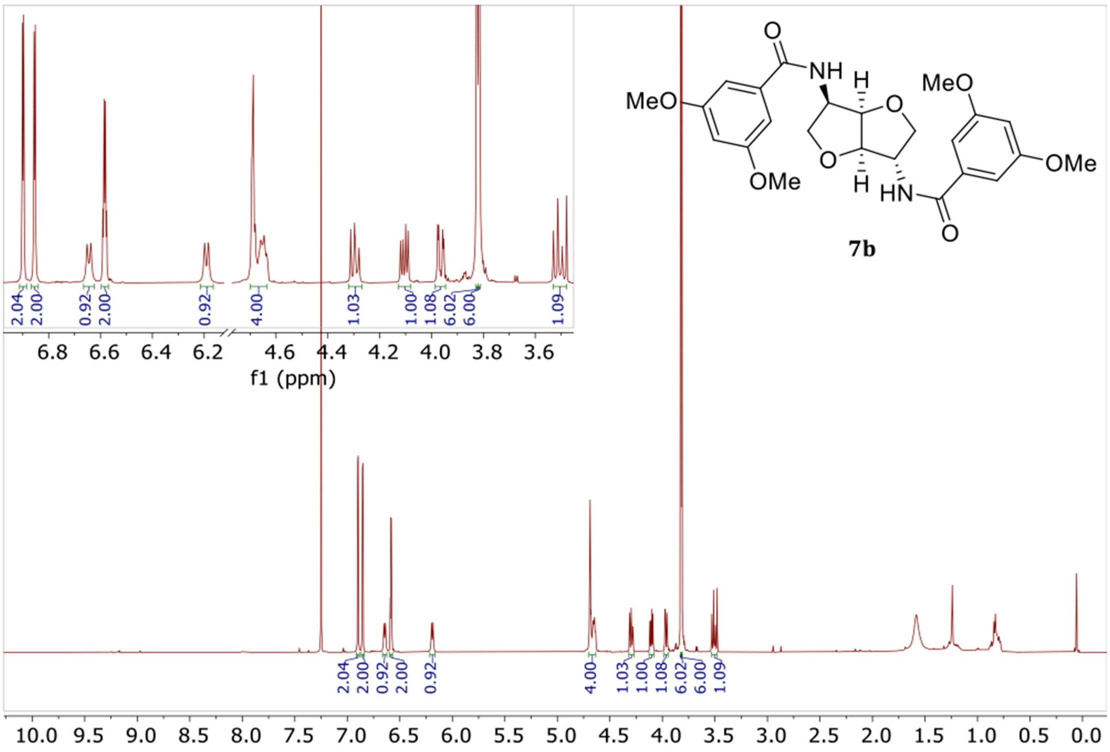

Figure SI28: ^1H NMR (400 MHz, [D_4]methanol, TMS, 21 °C, ppm) spectrum of **7b.**

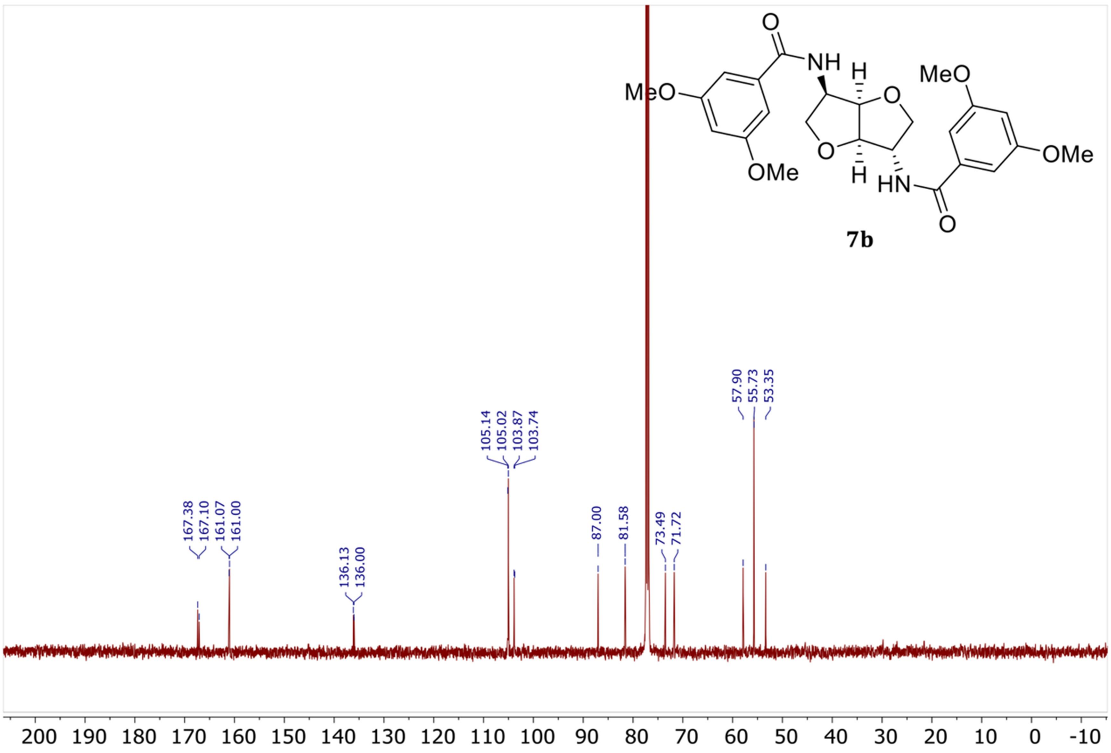

Figure SI29: $^{13}C\{^1H\}$ NMR (126 MHz, [D4]methanol, TMS, 21 °C, ppm) spectrum of **7b**.

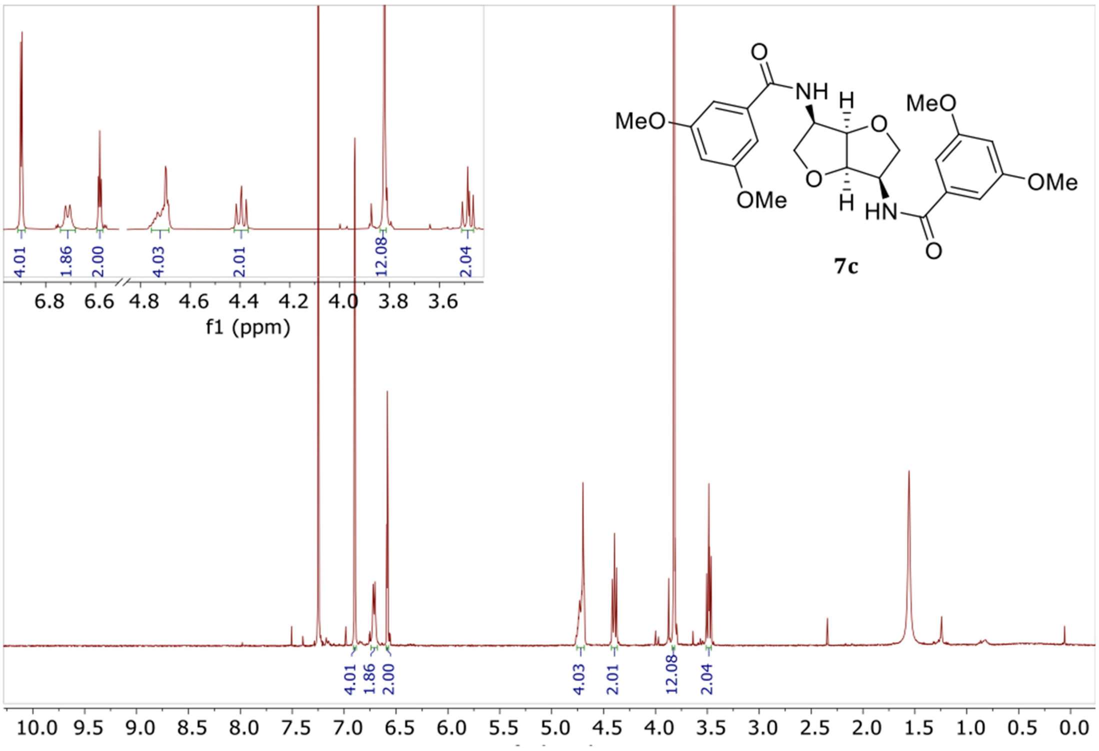

Figure SI30:^1H NMR (400 MHz, [D4]methanol, TMS, 21 °C, ppm) spectrum of **7c.**

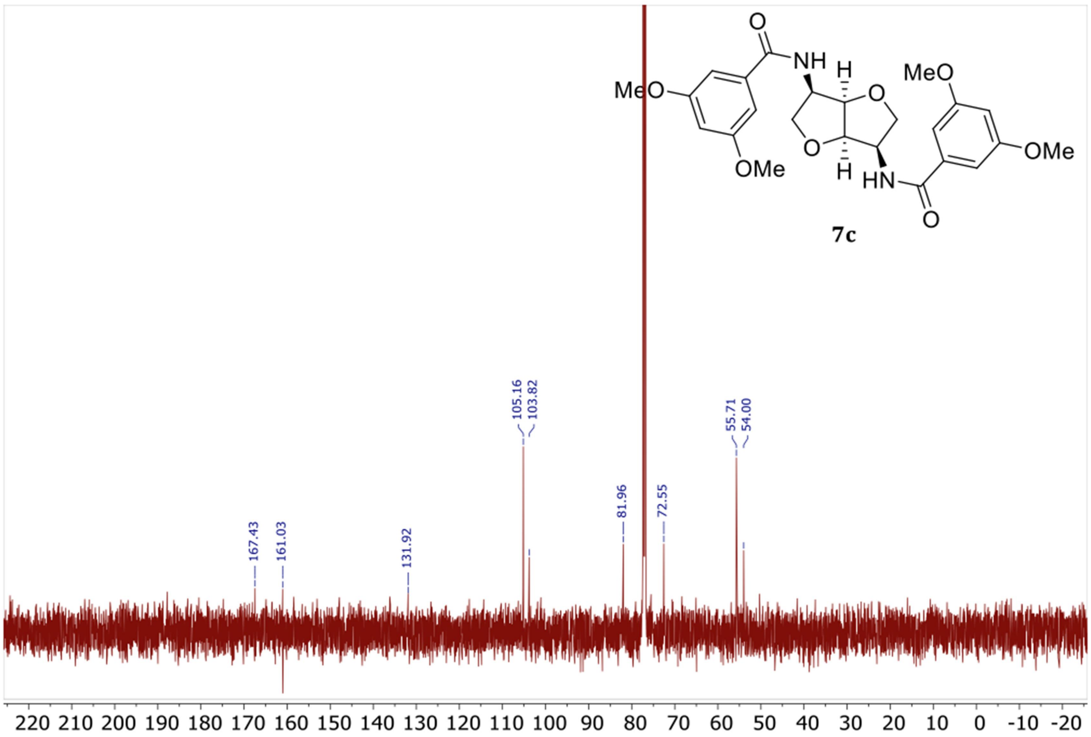

Figure SI31: $^{13}C\{^{1}H\}$ NMR (126 MHz, [D$_4$]methanol, TMS, 21 °C, ppm) spectrum of **7c**.

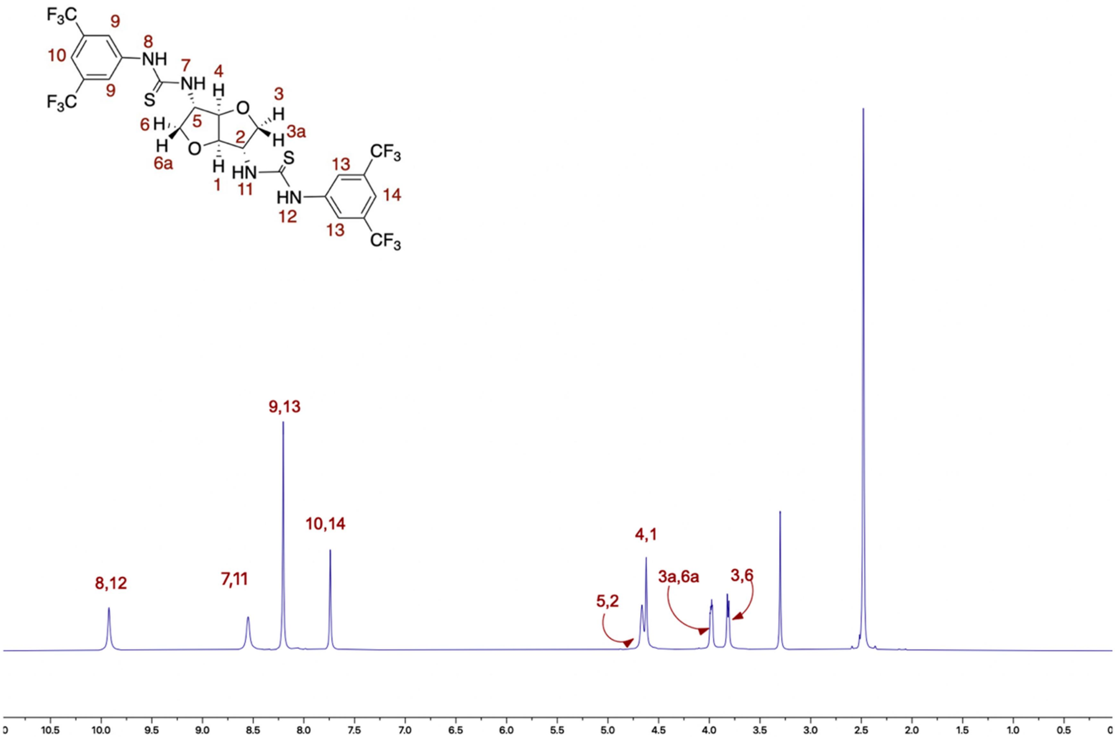

Figure SI32: ^{1}H NMR (600 MHz, [D$_6$]DMSO, TMS, 25 °C, ppm) spectrum of **8a**.

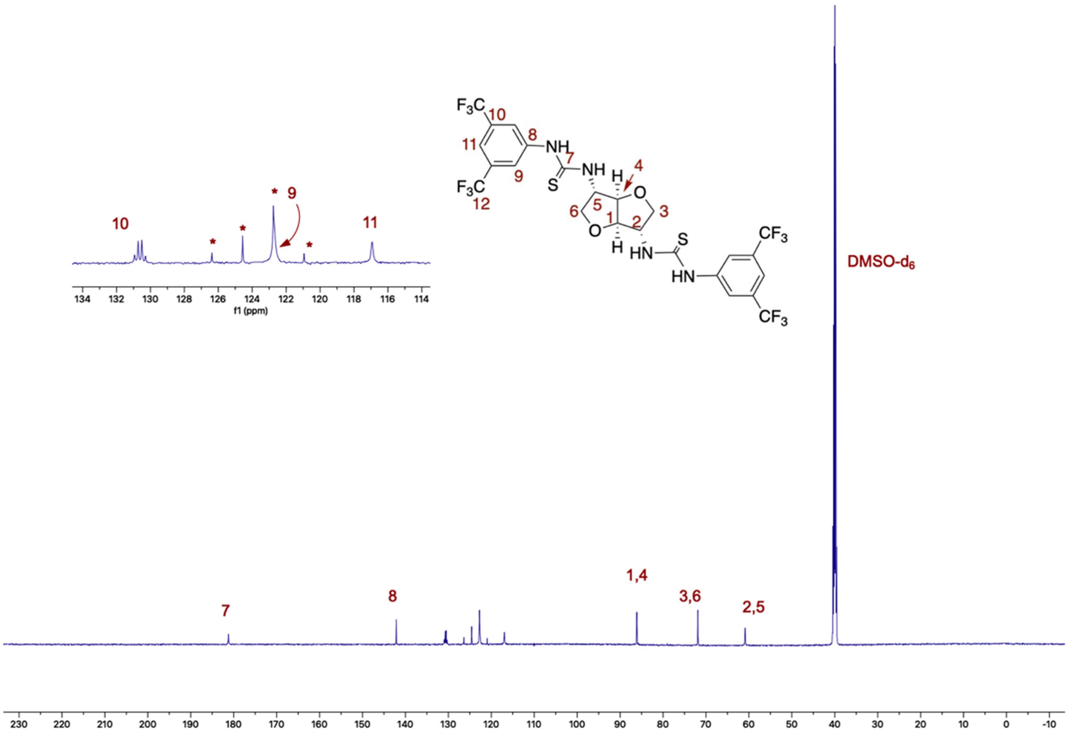

Figure SI33: $^{13}C\{^{1}H\}$ NMR (154 MHz, [D$_6$]DMSO, TMS, 25 °C, ppm) spectrum of **8a**.

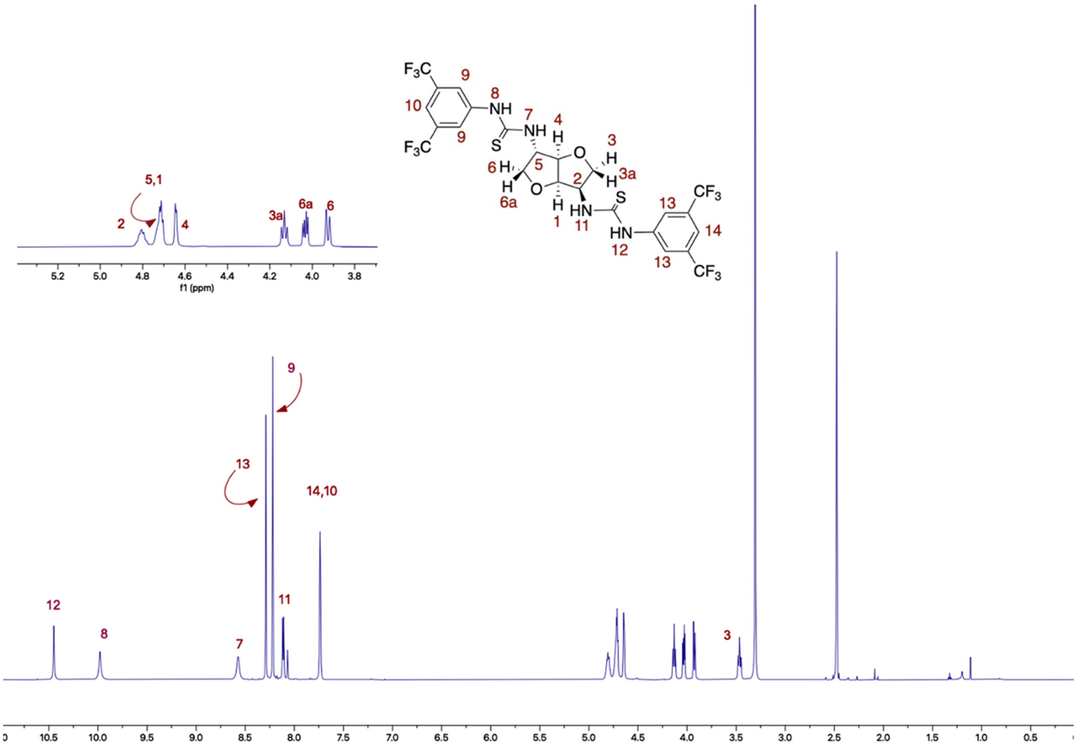

Figure SI34: ^{1}H NMR (600 MHz, [D$_6$]DMSO, TMS, 25 °C, ppm) spectrum of **8b**.

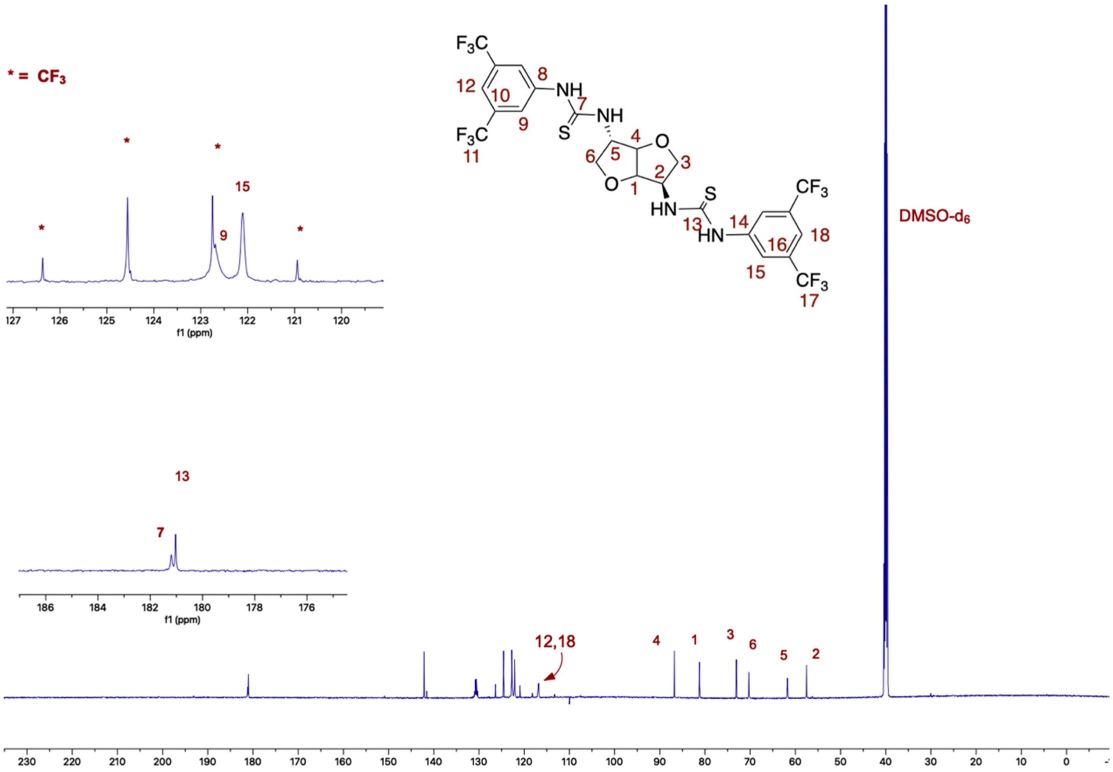

Figure SI35: $^{13}C\{^1H\}$ NMR (154 MHz, [D6]DMSO, TMS, 25 °C, ppm) spectrum of **8b**.

Optimization of the experimental conditions using in NMR enantiodiscrimination experiments.

In the preliminary part of the work, we have focused on the optimization of the experimental conditions about the enantiodiscrimination experiments using *N*-acetylleucine (**11a**) as chiral substrate. The choice of the solvent is influenced by the solubility of **8a**, which is limited in $CDCl_3$.

In a first attempt a 15 mM racemic **11a** in the presence of 1 equiv of **8a** and 1 equiv of DABCO in $CDCl_3$ was analyzed. Although the ^1H NMR chemical shift differentiation of the NH proton of the two enantiomers was satisfactory (0.055 ppm, Entry 1, Table I), there was a suspension in the sample, even in more diluted solutions (from 15 mM to 5 mM). Against the expectation, the nonequivalence was increased by four times decreasing the total concentration by a third (from 0.055 ppm to 0.195 ppm, Entry 2, Table I), despite the lack of homogeneity of the NMR sample.

To completely dissolve **8a**, [D_6]DMSO was employed as an alternative and uncommon solvent, but the competition between the polar solvent and the bis-thiourea totally hampered the discrimination phenomena (Entry 3, Table I).

For this motif, we returned on less polar solvents, like CD_2Cl_2 (Table I, entries 4-5) and C_6D_6 (Table I, entries 6-7). CD_2Cl_2 was the only solvent capable of completely dissolving the chiral auxiliary, while C_6D_6 was the best solvent about the discriminating ability of **8a** (0.308 and 0.080 ppm for the NH and acetyl protons of the 5 mM sample; Table 3, entry 7). CD_2Cl_2 and C_6D_6 have complementary solvating properties and for this reason we considered it appropriate to test three different binary mixtures of them: CD_2Cl_2/C_6D_6 (1:9), CD_2Cl_2/C_6D_6 (1:1) and CD_2Cl_2/C_6D_6 (3:7) (Table I, entries 8-10). The best nonequivalences and baseline separations were obtained in the third mixture (0.304 ppm and 0.083 ppm for NH and acetyl protons, respectively; Table I, entry 9) and all three samples were homogeneous at a 5 mM total concentration.

Table SI1. ^{1}H (600 MHz, 25 °C) nonequivalences ($\Delta\Delta\delta = |\delta_R - \delta_S|$, ppm) of selected protons of racemic **11a** in ternary mixture with 1 equiv of **8a** and 1 equiv of DABCO.

Entry	Solvent	Concentration (mM)	ΔΔδ	
			NH	Ac
**1**	$CDCl_3$	15	0.055	0.008
**2**	$CDCl_3$	5	0.195	0.002
**3**	[D_6]DMSO	15	0	0
**4**	CD_2Cl_2	10	0.154	0.010
**5**	CD_2Cl_2	5	0.173	0.010
**6**	C_6D_6	10	0.266	0.067
**7**	C_6D_6	5	0.308	0.080
**8**	CD_2Cl_2/C_6D_6 (1:9)	5	0.293	0.091
**9**	CD_2Cl_2/C_6D_6 (3:7)	5	0.304	0.083
**10**	CD_2Cl_2/C_6D_6 (1:1)	5	0.243	0.051

Considering the results obtained, ternary mixtures substrate/DABCO/**8a** (1:1:1) 5 mM in CD_2Cl_2/C_6D_6 3:7 were selected as standard condition for the preparation of the samples for the enantiodiscrimination experiments.

^{1}H and ^{19}F Enantiodiscrimination Experiments.

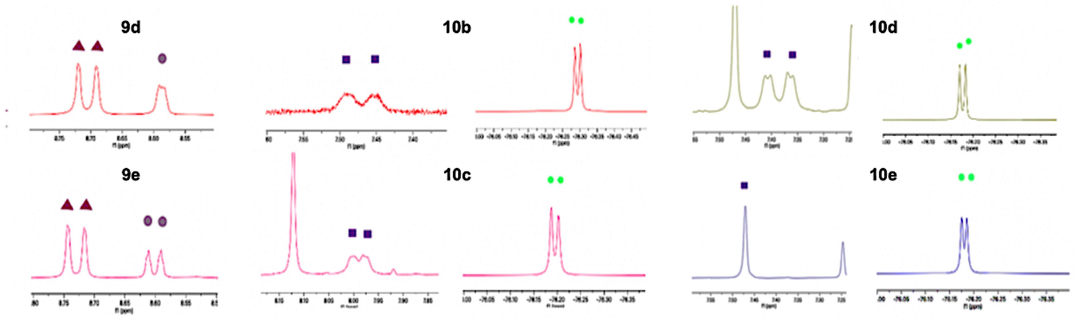

Figure S1: ^{1}H NMR (600 MHz, 25 °C) spectral regions corresponding to the *ortho*-DNB (▲) and *para*-DNB (●) resonances of **9d**-**e** (5mM) in equimolar mixture (5 mM) with **8a** and DABCO in CD_2Cl_2/C_6D_6 (3:7); ^{1}H NMR (600 MHz, 25 °C) and ^{19}F NMR (564 MHz, 25 °C) spectral regions corresponding to the NH (■) and CF_3CO (●) resonances of **10b**-**e** (5 mM) in equimolar mixture with **8a** and DABCO in CD_2Cl_2/C_6D_6 (3:7). NH resonances of **10b** were extracted by 1D TOCSY experiments carried out by selective perturbation of CH-α protons.

Table S1. Comparison between the ee (%) of enantiomerically enriched **11g** (5 mM) in presence of **8a**/DABCO (1:1) in CD_2Cl_2/C_6D_6 3:7 calculated from gravimetric data and determined by NMR integration of the NH group resonance of the substrate.

Actual ee (%)	ee by NMR (%)
87.9	87.9
79.7	79.7
63.4	63.4
45.9	45.9
30.5	30.5
16.9	16.9
1.70	1.30
6.20	6.4
-8.0	-8.2
-34.0	-33.9
-64.0	-64.1
-76.4	-76.4
-86.5	-86.5
-94.5	-94.7

^{1}H NMR configurational assignment.

Table S2. Complexation shifts ($\Delta\delta = \delta_{mix} - \delta_{free}$, ppm, 600 MHz, 25 °C) for NH and CH_3CO protons of **11a**-**c**,**f**,**g** (5 mM) in presence of 1 equiv of both **8a** and DABCO in CD_2Cl_2/C_6D_6 3:7.

**sub**	$\Delta\Delta\delta$		**sub**	$\Delta\Delta\delta$	
	NH	CH_3CO		NH	CH_3CO
**11a**	+ 0.368 (*R*)	+ 0.058 (*R*)	**11f**	+ 0.149 (*R*)	- 0.009 (*R*)
	- 0.028 (*S*)	- 0.034 (*S*)		+ 0.036 (*S*)	- 0.056 (*S*)
**11b**	+ 0.086 (*R*)	+ 0.025 (*R*)	**11g**	+ 0.306 (*R*)	+ 0.048 (*R*)
	- 0.105 (*S*)	- 0.039 (*S*)		+ 0.025 (*S*)	- 0.008 (*S*)
**11c**	- 0.144 (*R*)	- 0.022 (*R*)			
	- 0.478 (*S*)	- 0.130 (*S*)			

Investigation recognition processes.

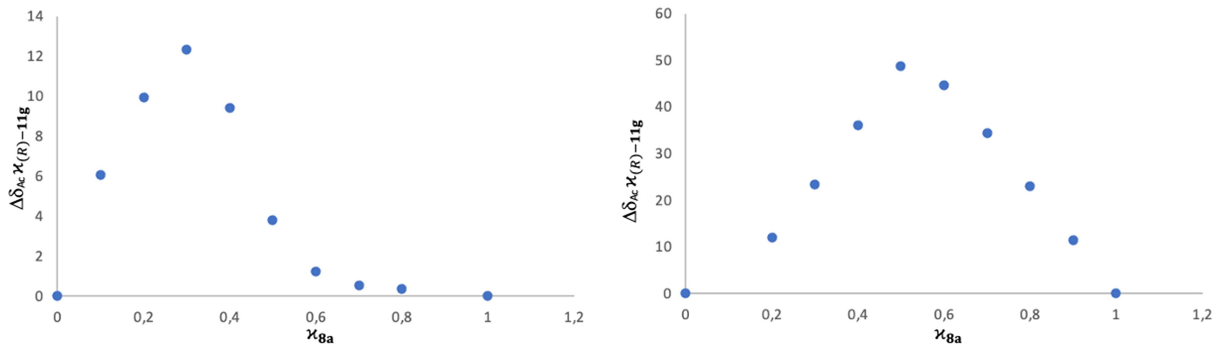

Figure S2: Complexation stoichiometry of (*R*)-**11g**/**8a**/DABCO (left) and (*S*)-**11g**/**8a**/DABCO (right) based on the ^{1}H resonances of the acetyl group of **11g**.

Table S3. ^{1}H NMR (600 MHz, 25 °C, CD_2Cl_2/C_6D_6 3:7,) chemical shifts (Hz) of the acetyl protons of (*S*)-**11g** (5 mM) in the mixture (*S*)-**11g**/**8a**/DABCO.

δ_{free}	δ_{mix}	$\Delta\delta_{Ac} = \delta_{mixture}$-$\delta_{free}$	$\chi_{(S)-7}$	$\Delta\delta_{Ac}\chi_{(S)-7}$
2741.67	2740.00	1.67	0.9	1.50
2741.67	2708.33	33.34	0.7	23.34
2741.67	2681.67	60.00	0.6	36.00
2741.67	2650.00	91.67	0.5	45.84
2741.67	2630.00	111.67	0.4	44.67
2741.67	2626.67	115.00	0.3	34.50
2741.67	2626.67	115.00	0.2	23.00
2741.67	2626.5	115.17	0.1	11.52

Table S4. ^{1}H NMR (600 MHz, 25 °C, CD_2Cl_2/C_6D_6 3:7) chemical shifts (Hz) of the acetyl protons of (*R*)-**11g** (5 mM) in the mixture (*R*)-**11g**/**8a**/DABCO.

δ_{free}	δ_{mix}	$\Delta\delta_{Ac} = \delta_{mixture}$-$\delta_{free}$	$\chi_{(R)-7}$	$\Delta\delta_{Ac}\chi_{(R)-7}$
986.38	993.13	6.75	0.9	6.08
986.38	998.80	12.42	0.8	9.94
986.38	1004.03	17.65	0.7	12.36
986.38	1002.07	15.69	0.6	9.41
986.38	994.01	7.63	0.5	3.82
986.38	989.43	3.05	0.4	1.22
986.38	988.12	1.74	0.3	0.52
986.38	988.12	1.74	0.2	0.35

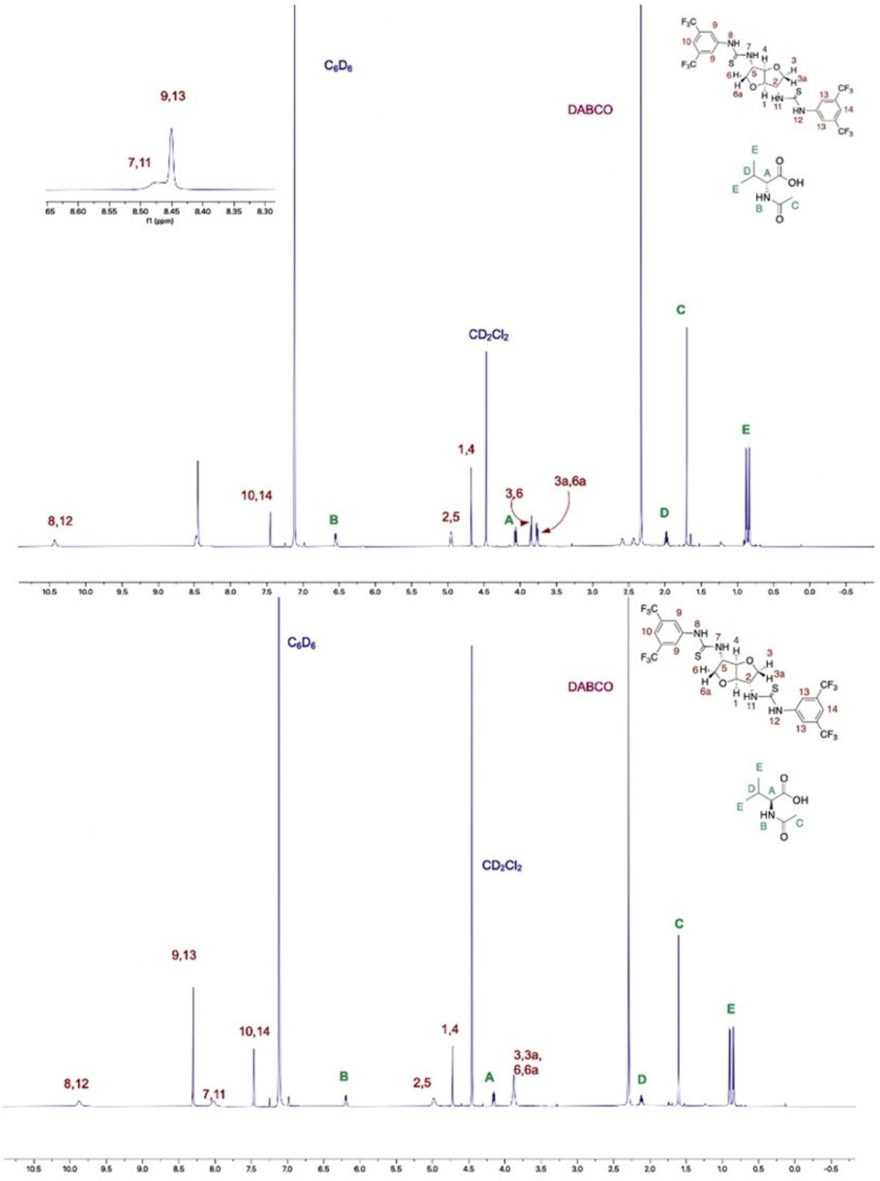

Figure S3: ^{1}H NMR (600 MHz, 25 °C, CD_2Cl_2/C_6D_6 3:7, ppm) spectra of the equimolar (5mM) mixtures (*R*)-**11g**/**8a**/DABCO (top) and (*S*)-**11g**/**8a**/DABCO (bottom).

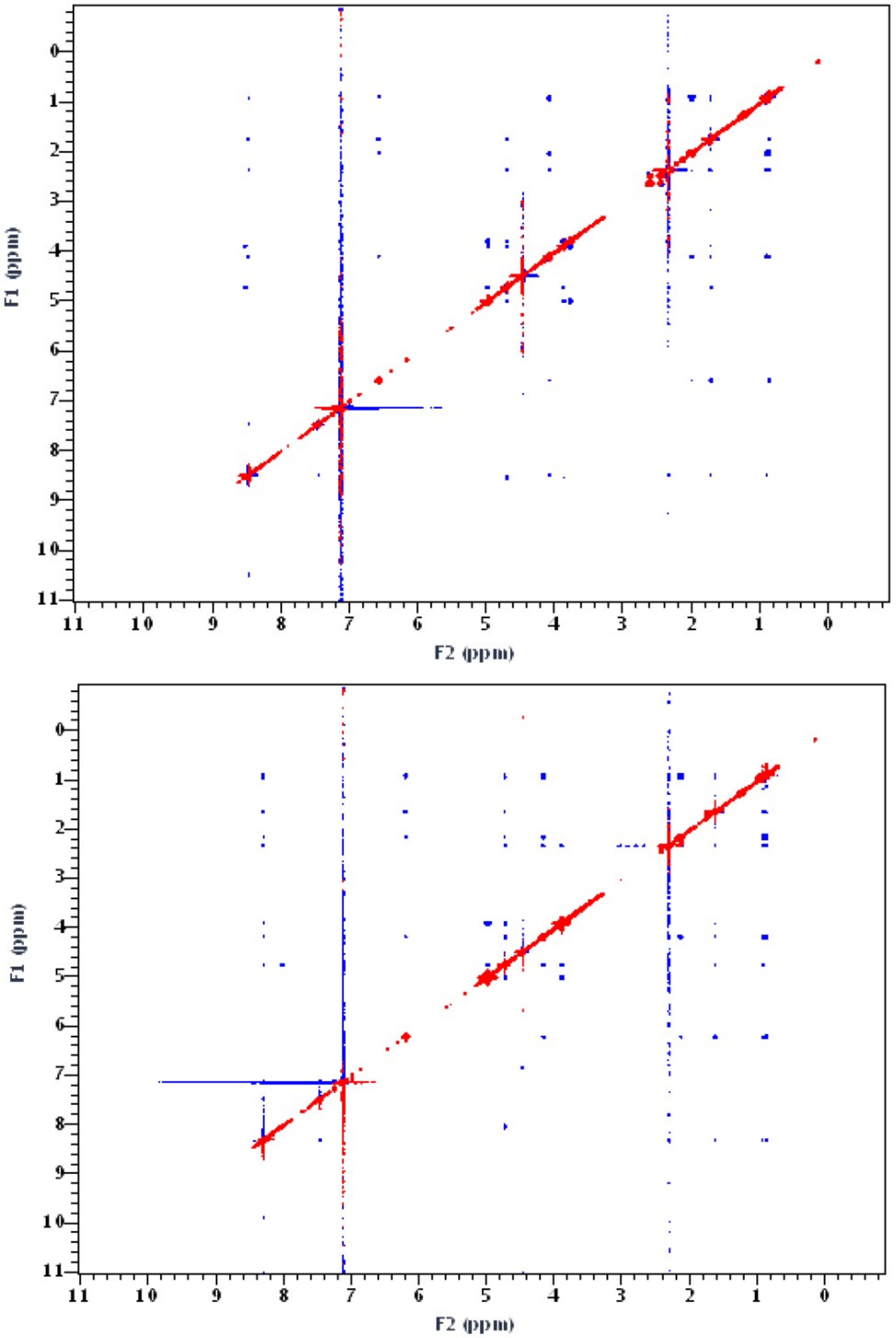

Figure S4: 2D ROESY (600 MHz, 25 °C, CD_2Cl_2/C_6D_6 3:7, mix 0.3 s) maps of the equimolar (5mM) mixtures (*R*)-**11g**/**8a**/DABCO (top) and (*S*)-**11g**/ **8a**/DABCO (bottom).

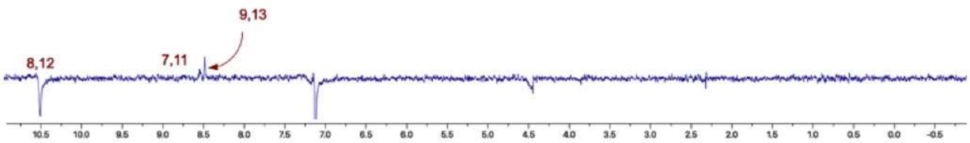

Figure S5: 1D ROESY (600 MHz, CD_2Cl_2/C_6D_6 (3:7), 25 °C, mix 0.5 s) spectrum corresponding to the selective perturbation of NH-8/NH-12 of (*R*)-**11g** (5mM) in the equimolar (*R*)-**11g**/DABCO/**8a**.

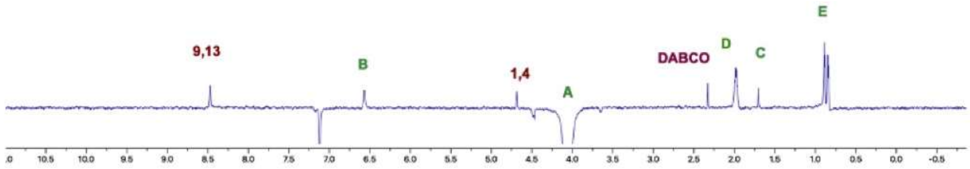

Figure S6: 1D ROESY (600 MHz, CD_2Cl_2/C_6D_6 (3:7), 25 °C, mix 0.5 s) spectrum corresponding to the selective perturbation of the proton H_A of (*R*)-**11g** (5mM) in the mixture (*R*)-**11g**/DABCO/**8a**.

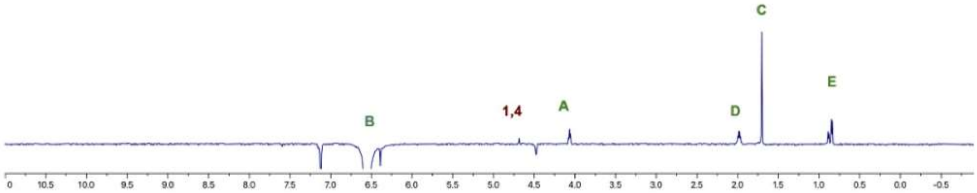

Figure S7: 1D ROESY (600 MHz, CD_2Cl_2/C_6D_6 (3:7), 25 °C, mix 0.5 s) spectrum corresponding to the selective perturbation of H_B of (*R*)-**11g** in the mixture (*R*)-**11g**/DABCO/**8a** (5mM)

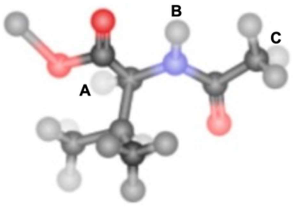

Figure S8: 3D representation of (*R*)-**11g**.

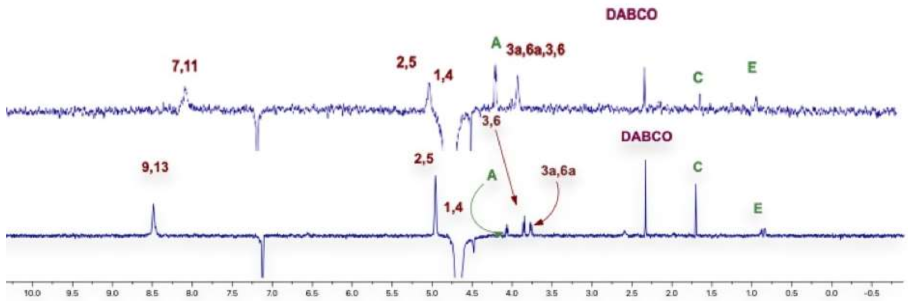

Figure S9: 1D ROESY (600 MHz, CD_2Cl_2/C_6D_6 (3:7), 25 °C, mix 0.5 s) spectra corresponding to the selective perturbation of H_1 and H_4 of **8a** in the equimolar mixtures (S)-**11g** (5mM)/DABCO/**8a** (top) and (R)-**11g** (5mM)/DABCO/**8a** (bottom).

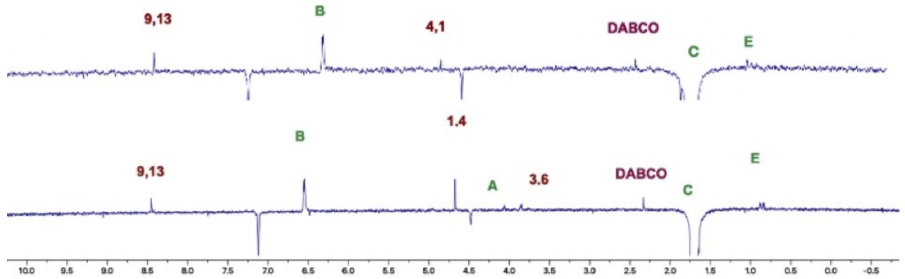

Figure S10: 1D ROESY (600 MHz, CD_2Cl_2/C_6D_6 (3:7), 25 °C, mix 0.5 s) spectra corresponding to the selective perturbation of H_C of **11g** in the equimolar mixtures (*S*)-**11g** (5mM)/DABCO/**8a** (top) and (*R*)-**11g** (5mM)/DABCO/**8a** (bottom).

Reference

1. De Coster, G.; Vandyck, K.; Van der Eychen, E.; Van der Eychen, J.; Elseviers, M.; Röper, H. D-isomannide in synthesis: asymmetric Diels-Alder reactions with novel homochiral bis-imine Cu^{2+}-catalyst. *Tetrahedron: Asymmetry* **2002**, *13*, 1673-1679, doi:10.1016/s0957-4166(02)00411-1
2. Wu, J.; Eduard, P.; Thiyagaraian, S.; Havaren, J.V.; Es, D.S.V.; Koning, C.E.; Lutz, M.; Guerra, C.F. Isohexide derivatives form renewable resources as chiral building blocks. *ChemSusChem* **2011**, *4*, 599-603, doi:10.1002/cssc.201100076
3. Chen, L.Y.; Guillarme, S.; Saluzzo, C. Dianhydrohexitols: new tools for organocatalysis. Application in enantioselective Friedel-Crafts alkylation of indoles with nitroalkenes. *Arkivoc* **2013**, 227-244, doi:10.3998/ark.5550190.0014.318
4. La Scala, J.J.; Yandek, G.; Sadler, J.M. The effect of methyl and methoxy substituents on dianilines for a thermosetting polyimide system. *High Perform. Polym*. **2020**, *32*, 801-822, doi:10.1177/0954008319899141.
5. Barros, T.G.; Pinheiro, S.; Williamson, J.S.; Tanuri, A.; Gomes Jr, M.; Pereira, H.S.; Brindeiro, R.M.; Neto, J.B.A.; Antunes, O.A.C.; Muri, E.M.F. Pseudo-peptides derived form isomannide: inhibitors of serine proteases. *Amino Acids* **2010**, *38*, 701-709, doi:10.1007/s00726-009-0273-4.
6. Archibald, T.G.; Baum. K. Synthesis of polynitro-2,6-dioxabicyclo[3.3.0.]octanes. *Synth. Commun*. **1989**, *19*, 1493-1498, doi:10.1080/00397918908051042
7. Naturale, G.; Lamblin, M.; Commandeur, C.; Felpin, F.X.; Dessolin. Direct C-H alkylation of naphthoquinones with amino acids through a rivisited Kochi-Anderson radical decarboxylation: trend and reactivity and application. *Eur. J. Org. Chem*. **2012**, *29*, 5774-5788, doi:10.1002/ejoc.201200722
8. Natanael, J.G.; Ancock, A.H.; Wille, U. Reaction of amino acids, di- and tripeptides with the environmental oxidant NO_3^{-}: a laser flash photolysis and computational study. *Chem. Asian J*. **2016**, *11*, 3188-3195, doi:10.1002/asia.201600994
